# Programmed catalysis within stimuli-responsive mechanically unlocked nanocavities in DNA origami tiles†

**DOI:** 10.1039/d0sc04108d

**Published:** 2020-10-27

**Authors:** Jianbang Wang, Zhixin Zhou, Zhenzhen Li, Itamar Willner

**Affiliations:** Institute of Chemistry, The Center for Nanoscience and Nanotechnology, The Hebrew University of Jerusalem Jerusalem 91904 Israel Itamar.willner@mail.huji.ac.il

## Abstract

The assembly of reversible stimuli-responsive locked DNA origami tiles being unlocked, in the presence of appropriate triggers, to form nanocavities in the origami rafts, is introduced. In the presence of ATP, K^+^-ion-stabilized G-quadruplexes or pH-responsive T-A·T triggers and appropriately engineered “helper units”, the origami rafts are unlocked to form nanocavities. By the application of appropriate counter-triggers, the nanocavities are relocked, thus establishing the switchable and reversible “mechanical” opening and closure mechanism of the nanocavities. The interconnection of the stimuli-responsive origami tiles into dimer structures enables the programmed triggered unlocking of each of the origami tiles, or both of the origami tiles, to yield dictated nanocavity-containing tiles. In addition, the functionalization of the opposite faces of the origami tiles with Mg^2+^-ion-dependent DNAzyme subunits leads, upon the triggered unlocking of the nanocavities, to the self-assembly of the active DNAzymes in the confined cavities. By the cyclic opening and closure of the cavities the reversible “ON”/“OFF” activation of the Mg^2+^-ion-dependent DNAzyme is demonstrated. Furthermore, upon the tethering of different Mg^2+^-ion-dependent subunits to the opposite faces of stimuli-responsive dimer origami tiles, the triggered programmed catalytic operation of different Mg^2+^-ion-dependent DNAzymes in the confined nanocavities, associated with the origami tiles, is demonstrated.

## Abstract 2

Programmed unlocking of nanocavities in origami dimer structures using different auxiliary triggers.

## Graphical abstract

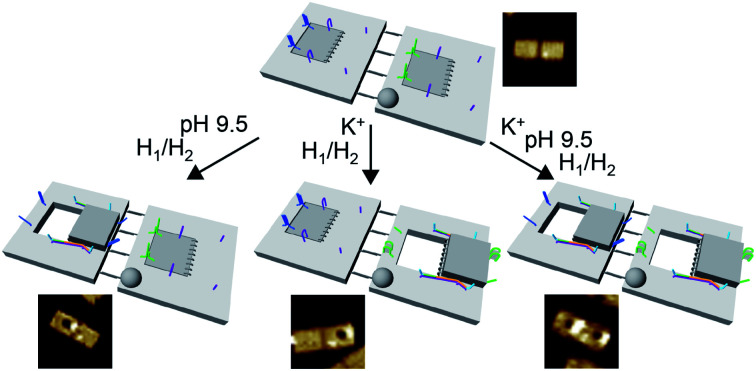

## Introduction

The assembly of two-dimensional (2D) and three-dimensional DNA origami nanostructures introduced an important direction in the area of DNA nanotechnology.^1^ Besides the design of ingenious DNA origami structures by folding of the long-chain M13 phage DNA with dictated “staple” units, the functionalization of DNA origami tiles with protruding nucleic acids or the modification of the tile edges with nucleic acid tethers led to functional modules for many different applications.^2,3^ The spatial and ordered binding of proteins,^4^ nanoparticles^5–7^ and polymers^8,9^ to the protruding tethers associated with origami tiles was used to operate enzyme cascades^10,11^ and to develop plasmonic antennae^12,13^ and plasmonic structures.^14–16^ In addition, the origami tiles were used as functional scaffolds for operating DNA machines such as walkers,^17–20^ “arms”^21^ or chiroplasmonic nanostructures.^14,22–24^ The edge-modification origami structures were used to assemble linear and bent origami dimer and trimer structures. The linking of origami units using stimuli-responsive bridges allowed the triggered reversible association/dissociation and the isomerization of the structures using pH,^25^ G-quadruplex/crown ether,^26^ and light^27^ as triggers. Different applications of origami structures were reported, and these included their use as stimuli-responsive drug carriers,^28,29^ sensors^30^ and logic gate operators.^31,32^ In addition, origami nanostructures were applied for the “bottom-up” fabrication of nanochannels on nanoholes for the programmed deposition of functional units into confined nano-environments. For example, nanocavities were introduced into origami tiles by the passive assembly of “stapled” origami rafts,^33,34^ and the cavities were used for the specific docking of antibodies^35^ and for the reconstitution of membrane proteins^36^ in confined environments. Recently, we introduced an active molecular process for the generation of nanoholes in origami tiles by designing locked “patches” as a part of the origami tiles.^37^ The respective locks consisted of metal-ion-dependent DNAzyme/substrate units or *trans*-azobenzene duplex-stabilized units. After “opening” the locks, the “patches” were “mechanically” opened over a set of “hinges”, using arms attached to the “patches” and “helper” strands that tied the patches to “foothold” sites, associated with the origami rafts, to retain the cavities in open configurations.^37^ The DNAzyme-triggered unlocking of the locks, by the cleavage of the DNAzyme/substrate units that were part of the locks, was however, a single cycle process, and the cavities retained permanent open structures. The photochemical unlocking of the locks using photoisomerizable *trans*/*cis* azobenzene units revealed, however, reversible light-induced opening and closure functions. These primary studies call for efforts to design other reversible locks for the triggered opening and closure of nanocavities in DNA origami rafts. Furthermore, the feasibility of engineering origami dimer structures (or structures of higher complexity), suggests that developing multi-triggered, reversible, unlocking mechanisms of nanocavities in dimer origami structures could lead to the programmed fabrication of nanocavities in complex origami structures.^38^ Design of such systems would allow the design of programmed multiplexed chemical transformations in the confined nanocavities. In the present study, we report the generation of nanocavities in origami tiles using the sequences of G-quadruplexes, ATP–aptamer complexes and T-A·T triplexes as locks. These locks allow the reversible opening and closure of the nanocavities. In addition, we demonstrate that the assembly of DNA-origami dimers consisting of rafts being unlocked by two different triggers allows the programmed generation of nanocavities in the dimer origami assemblies. Furthermore, by encoding “functional information” in the resulting nanocavities, trigger-guided programmed catalytic functions in the confined nano-cavities are realized. In contrast to our previous report^37^ where the unlocking of the “hole” was driven by the irreversible mechanical degradation of the locking units by metal-ion-dependent DNAzymes, we introduce here a set of reversible locking/unlocking modules consisting of K^+^-ion-stabilized G-quadruplex/crown ether, aptamer–ligand complexes and pH-responsive locking/unlocking units.

## Results and discussion

**Fig. 1:**
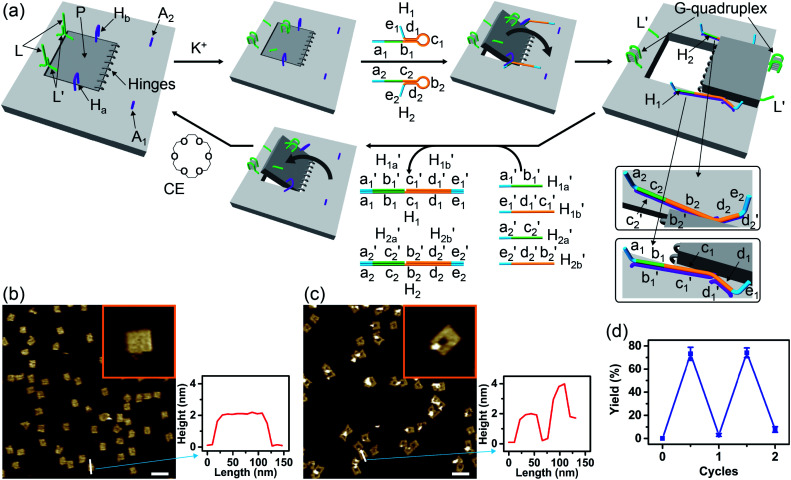
(a) Schematic K^+^-ion-stimulated “mechanical” unlocking and the generation of nanoholes in the origami raft through the formation of G-quadruplexes, and the reversible closure of the nanoholes in the presence of the counter-helper strands and the 18-crown-6-ether (CE) (only showing the key part of the tile). (b) AFM image and cross-sectional analysis of the locked intact origami tiles. Inset: enlarged single tile. Scale bar: 200 nm. (c) AFM image and the corresponding cross-sectional analysis of the K^+^-ion-induced unlocked nanocavities in the origami tiles. Scale bar: 200 nm. (d) Cyclic yields of the unlocked origami tiles generated reversibly in the presence of K^+^ ions/H_1_/H_2_ and 
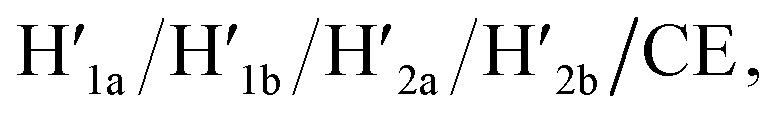
 respectively.

Fig. 1a depicts schematically the reversible opening and closure of nanocavities in a DNA origami raft using K^+^-ion-stabilized G-quadruplexes and 18-crown-6-ether (CE) as reversible triggers for the unlocking and relocking of the nanocavities generated in the origami tiles.^39^ For the detailed composition of the origami rafts and the respective composition of the K^+^-ion-stabilized G-quadruplex/CE locking environment see the ESI, Fig. S1.† The DNA “patch”, P, is a part of the origami tile. It is linked to the tile with eight DNA strands acting as “hinges”. The patch is locked to the origami tile with two duplex locks consisting of strands L/L′. The strand L includes the G-quadruplex sequence in a caged configuration. The patch is further linked to the tile through two “arms” H_a_ and H_b_. In addition, two anchor footholds A_1_ and A_2_ are linked as protruding tethers to the tile. In the presence of K^+^ ions, the locks are unlocked through the formation of K^+^-ion-stabilized G-quadruplexes, and in the presence of the helper hairpins H_1_ and H_2_, acting as “helper” strands, the patch is “mechanically” driven to open the nanocavity, by swinging the “patch” onto the origami tile through the hybridization of the hairpins H_1_ and H_2_ with the “arms” and fixing the open hairpin domains by hybridization with the anchoring footholds A_1_ and A_2_. (To further clarify the opening mechanism between the “arms” and the “helper” hairpins, we exemplify the stepwise opening of arm H_a_ by hairpin H_1_. A sequence domain of H_a_ hybridizes with the domain of the single strand tether [green] of H_1_ followed by strand displacement of the stem region to yield a free single strand that stretches the patch and hybridizes with the anchor strand A_1_.) Treatment of the open configuration of the nanocavities with the counter-helper strands 
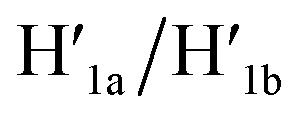
 and 
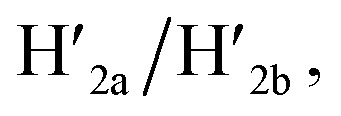
 and in the presence of 18-crown-6-ether leads to the displacement of the stretching strands in the form of the “waste” duplexes 
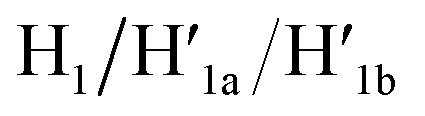
 and 
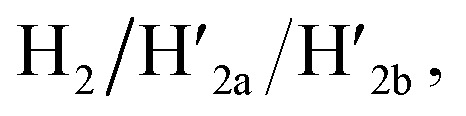
 and to the separation of the G-quadruplexes. This results in the dissociation of the patch from its firm fixation on the anchoring sites and in the closure of the cavity by the regeneration of the stable L/L′ locked structure. That is, the system acts as a “mechanical window” undergoing reversible opening in the presence of K^+^ ions and the hairpins H_1_ and H_2_ and reverse closure in the presence of the counter strands 
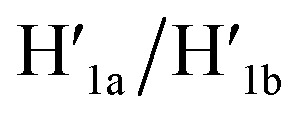
 and 
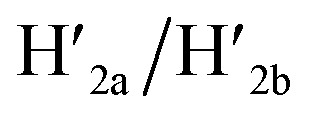
 and 18-crown-6-ether. Fig. 1b–c show the atomic force microscopy (AFM) images before and after the treatment of the origami tiles with K^+^ ions and the hairpins H_1_ and H_2_ and the respective cross-sectional analyses of the respective tile. The parent tiles show intact 100 × 80 nm areas with a characteristic height of 2 nm; Fig. 1b and the corresponding inset. After the treatment of the tiles with K^+^ ions, and the hairpins H_1_ and H_2_, the tiles turn to the configurations with cavities, *ca.* 20 nm in diameter. Each of the tiles includes a bright spot that corresponds to a swing-over patch (“window”) lying on the origami scaffold; Fig. 1c and the inset. Cross-sectional analysis of the resulting tile reveals the height of the origami plane (2 nm), followed by a vacant hole domain, *ca.* 20 nm, and a high domain, *ca.* 3.9 nm, that corresponds to the double thickness tile of the “window” lying on the base origami scaffold. Analysis of four 2 × 2 μm large-area images indicates a 75% yield of the cavity-containing tiles. By applying K^+^ ions and “helper” strands H_1_/H_2_ and the counter strands 
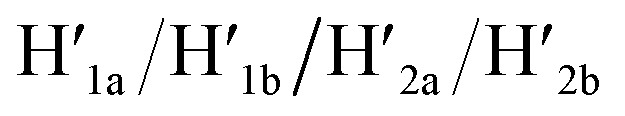
 and CE, respectively, the unlocking and relocking of the origami tiles and the corresponding yields of the cavity-containing tiles are presented (Fig. 1d, S2–S6 and Tables S1–S5†). It should be noted that control experiments indicated that the addition of K^+^ ions and the hairpins H_1_ and H_2_ is essential to yield the hole containing tiles. While in the absence of K^+^ ions no hole-containing tiles could be detected, in the absence of H_1_ and H_2_ a very low yield of hole-containing tiles (*ca.* 9%) could be identified, implying that the swing stretching and rigidification of the unlocked patches are essential to yielding the cavities (Fig. S7 and S8, Tables S6 and S7†). It should be noted that the AFM images show some structures appearing as “dimers”. These structures originate however from coincidental non-specific interactions between monomer origami tiles. Electrophoretic measurements following the cyclic opening and closure of the origami tiles, Fig. S9,† indicate pure origami monomer structures in the respective systems.

**Fig. 2:**
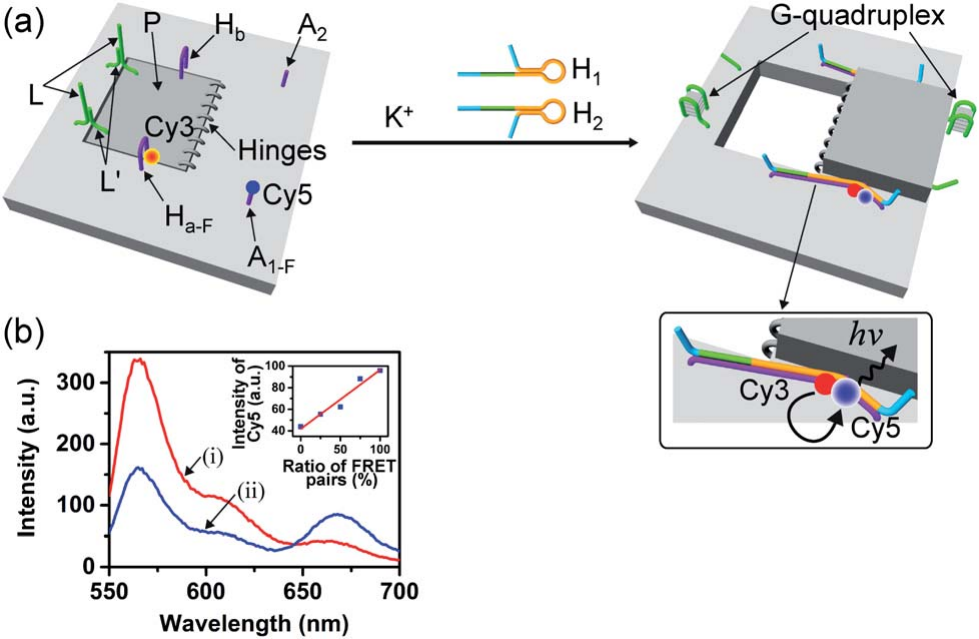
(a) Following the “mechanical” K^+^-ion-induced unlocking of the nanocavities in the origami tiles using the FRET mechanism. (b) Fluorescence spectra of the Cy3 and Cy5 fluorophore-labeled origami tiles using Cy3 as the donor fluorophore and Cy5 as the acceptor fluorophore: (i) in the locked configuration of the tile; (ii) in the presence of the unlocked, cavity-containing tiles. Inset: calibration curve, corresponding to the FRET signal generated in the presence of Cy3-modified H_a-F_ (24 nM) and Cy5-functionalized A_1-F_ (24 nM) in the presence of variable concentrations of the linker H_1-F_.

The G-quadruplex-driven unlocking of the “window”, its opening through stretching by means of the “helper” strands and its fixation on the anchoring foothold A_1_ and A_2_ was further supported by Förster Resonance Energy Transfer (FRET) experiments, Fig. 2. The arm H_a-F_ was internally modified with the Cy3 fluorophore and the foothold A_1-F_ was functionalized with the Cy5 fluorophore. The G-quadruplex-stimulated unlocking of the patch and the opening of the “window” by the “helper” strands H_1_ and H_2_ that hybridize with the arms H_a-F_ and H_b_, respectively, linking to the anchoring footholds, lead to an intimate spatial distance between Cy3 and Cy5, Fig. 2a, thus allowing energy transfer between Cy3 and Cy5. Fig. 2b shows the fluorescence spectra of the tile before unlocking the “window”, curve (i), and after unlocking and opening of the “window”, curve (ii), upon excitation of Cy3 at 532 nm. In the presence of the closed “window” configuration, an intense fluorescence of Cy3 at *λ* = 564 nm is observed, and a very low fluorescence at *λ* = 665 (the emission wavelength of Cy5) is detected. The opening of the “window” leading to the decrease of the fluorescence intensity of Cy3 at *λ* = 564 nm and to an intensified fluorescence of Cy5 at *λ* = 665 nm is observed. Thus, the opening of the “window” leads to a FRET signal between Cy3 and Cy5, consistent with the formation of a close spatial distribution of the donor–acceptor fluorophores. By extracting a calibration curve corresponding to the intensities of the fluorescence of Cy5 at different percentage rates of the FRET pairs, the inset in Fig. 2b, we estimate from the fluorescence intensity of Cy5 that *ca.* 75% FRET pairs are generated upon opening the “window”. This result is consistent with the yields of the open cavity-containing tiles, evaluated using the AFM images of the system.

**Fig. 3:**
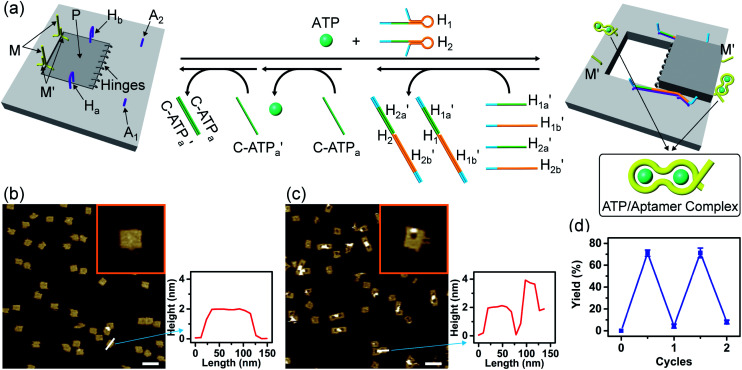
(a) Schematic ATP-driven “mechanical” unlocking of nanocavities in origami tiles and their reversible locking by means of counter agents separating the ATP–aptamer complexes. (b) AFM image and cross-sectional analysis of the locked ATP-responsive origami tiles. Inset: enlarged AFM image of the locked ATP-responsive tile. Scale bar: 200 nm. (c) AFM image of the unlocked, nanocavity-containing, ATP-responsive origami tiles, and the respective cross-sectional analysis. Scale bar: 200 nm. (d) Cyclic triggered yields of the unlocked origami tiles generated in the presence of ATP/H_1_/H_2_ and the counter agents 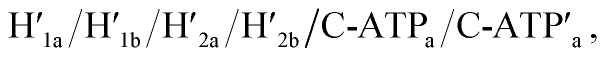 respectively.

Fig. 3 depicts the schematic reversible unlocking and locking of the nanoholes by the ATP–aptamer complexes. For the detailed composition of the origami tiles and the respective ATP aptamer locking/unlocking environment, see the ESI, Fig. S10.† The patch domain is an integrated part of the origami tile and linked to the origami scaffold with eight strands, acting as “hinges”, and two duplex locks M/M′. The strands M in the locks include the ATP aptamer sequence in a duplex caged configuration. As before, the patch is modified at opposite positions with the “arms” H_a_ and H_b_, and the origami scaffold is functionalized with the protruding foothold anchoring tethers, A_1_ and A_2_. In the presence of ATP and the hairpins H_1_ and H_2_, the two locks are unlocked by forming the ATP–aptamer complexes, and the nanoholes are formed by the H_1_ and H_2_-stimulated binding to the arms H_a_/H_b_, swinging the patches across the “hinges” and stretching the “windows” on the origami rafts with their fixation to the footholds A_1_ and A_2_. Treatment of the “open window” tiles with the counter-helper strands, 
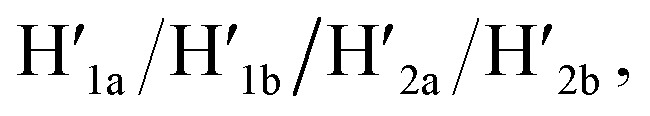
 and the counter-ATP aptamer strand, C-ATP_a_, results in the displacement of the “helper” strands H_1_/H_2_ from the footholds A_1_/A_2_ and the separation of the ATP–aptamer complexes. This yields the flexible “window” structures and after washing off the free ATP and the subsequent release of the ATP aptamer through the displacement of C-ATP_a_ by 
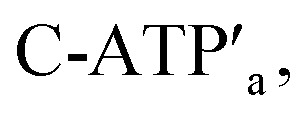
 the recovered ATP aptamer strands allow the relocking of the cavities by their hybridization with the strands M′. Fig. 3b shows the AFM image of the parent locked tiles (height 2 nm). After treatment of the locked structures with ATP and hairpins H_1_/H_2_ the unlocking of the “windows” proceeds, accompanied by the formation of the nanoholes (*ca.* 20 nm diameter) and the fixation of the “windows” on the origami scaffolds by stretching the “windows” and their fixation on the footholds A_1_/A_2_, Fig. 2c. This is evident from the cross-sectional analysis of the tiles that reveals the characteristic height of the origami tile, 2 nm, followed by the cavity and a double-height structure of the “window”, resting on the base origami tile (height 1.9 nm); Fig. 3c. Statistical analysis of four 2 × 2 μm large-scale AFM images indicates a yield of open-hole tiles that corresponds to *ca.* 70%. By the cyclic treatment of the tiles with ATP and the stretching of hairpin H_1_ and H_2_, followed by the treatment of the cavity-containing tiles with the counter-stretching strands 
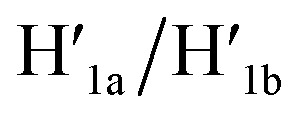
 and 
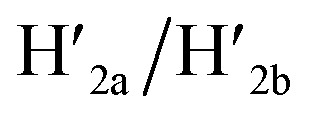
 and C-ATP_a_ and the subsequent treatment of the system with the 
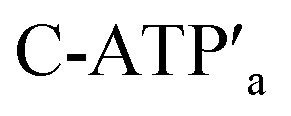
 strands, the reversible opening and closure of the “windows” are demonstrated (Fig. 3d, Fig. S11–S15 and Tables S8–S12†). As before, the stretching of the “windows” by H_1_ and H_2_ and their fixation on the footholds A_1_ and A_2_, upon their unlocking with ATP, are essential to generating the high-yield cavity-functionalized tiles. In the absence of the stretching strands, the yield of identified hole-modified tiles is only *ca.* 9% (Fig. S16 and S17, Tables S13 and S14†).

**Fig. 4:**
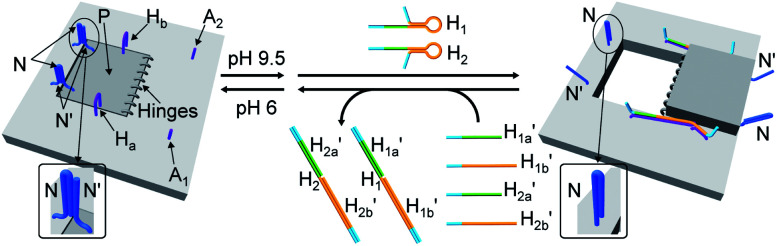
Schematic pH-driven opening of the T-A·T triplex locked origami raft in the presence of the “helper” hairpins H_1_ and H_2_ to yield the nanocavity-containing tiles (at pH = 9.5) and their reversible closure at pH = 6.0 in the presence of the counter strands 
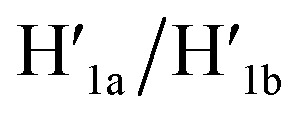
 and 
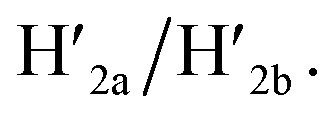

The third module to lock the patch in the integrated tile configuration included the T-A·T pH-responsive locks,^40^ as described in Fig. 4. For the detailed composition of the origami tiles and the pH responsive lock see the ESI, Fig. S18.† The patch is integrated in the origami tile using the eight “hinges” and applying two locks consisting of the T-A·T triplexes, at pH = 6, composed of the duplexes N and the single strands N′. The patch includes at two opposite sides the “arms” H_a_ and H_b_, and the protruding anchoring tethers A_1_ and A_2_ are engineered as functional components in the tiles. At pH = 9.5 the triplex locks are separated to yield the unlocked patches, and in the presence of the “helper” hairpins H_1_ and H_2_ that hybridize with the “arms”, H_a_/H_b_, the swinging of the “windows” over the “hinges” and the rigidified positioning of the “windows” on the origami rafts through anchoring the stretching strands on the footholds A_1_ and A_2_ proceed to yield the nanocavities in the origami tiles. As before, treatment of the “open-window” origami structures with the counter-helper strands, 
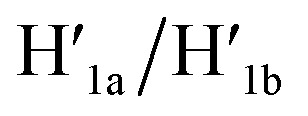
 and 
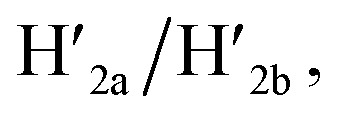
 the stretching strands are displaced from the anchoring footholds and “arms” in the form of “waste” duplexes 
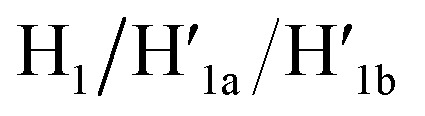
 and 
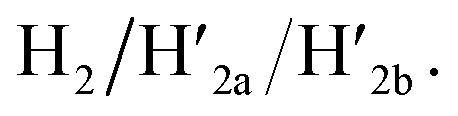
 This results in the flexible “window” domains that regenerate, at pH = 6.0, the integrated closed “window” using firm T-A·T triplexes and N/N′ locks. The experimental results that follow the reversible pH-induced unlocking of “windows” in the origami rafts and their recovery to the locked origami configuration are presented in Fig. S19, ESI.† Fig. S19a† shows the integrated N/N′ locked rafts and Fig. S19b† shows the cavity-containing origami rafts generated at pH = 9.5 in the presence of hairpins H_1_ and H_2_. The yield of the cavity-containing structures is *ca.* 75%, and the cross-sectional analysis reveals the formation of holes with a diameter of *ca.* 20 nm. The pH-stimulated opening and closure of the cavities is reversible and the cyclic treatment of the closed raft at pH = 9.5, in the presence of H_1_ and H_2_, yields the hole-containing rafts while treatment of the cavity-containing tiles at pH = 6.0, with the counter strands 
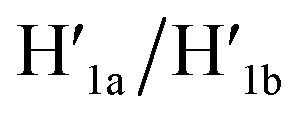
 and 
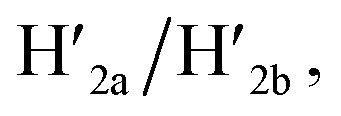
 regenerates the intact closed raft configuration (Fig. S19c, S20–S24 and Tables S15–S19†). As before, the stretching of the “windows” by H_1_ and H_2_ and their fixation on the footholds A_1_ and A_2_, upon their unlocking at pH = 9.5, are essential to generating the high-yield cavity-containing tiles. In the absence of the stretching hairpin strands, the yield of identified hole-modified tiles is *ca.* 9% (Fig. S25 and S26, Tables S20 and S21†). It should be noted that the reversible operation of the opening and closure of the holes in Fig. 1, 3 and 4 is demonstrated for two cycles. In principle, the cycling can be extended. However, the repeated addition of "helper" hairpins and counter helper strands, and the need to purify the tiles from crown ether and ATP and the change of the pH in the respective systems diluted the analysed samples and perturbed the reversible feature of the systems. We find that after three reversible cycles, the switching efficiency of the reversible cycles decrease by 50% to 60%.

**Fig. 5:**
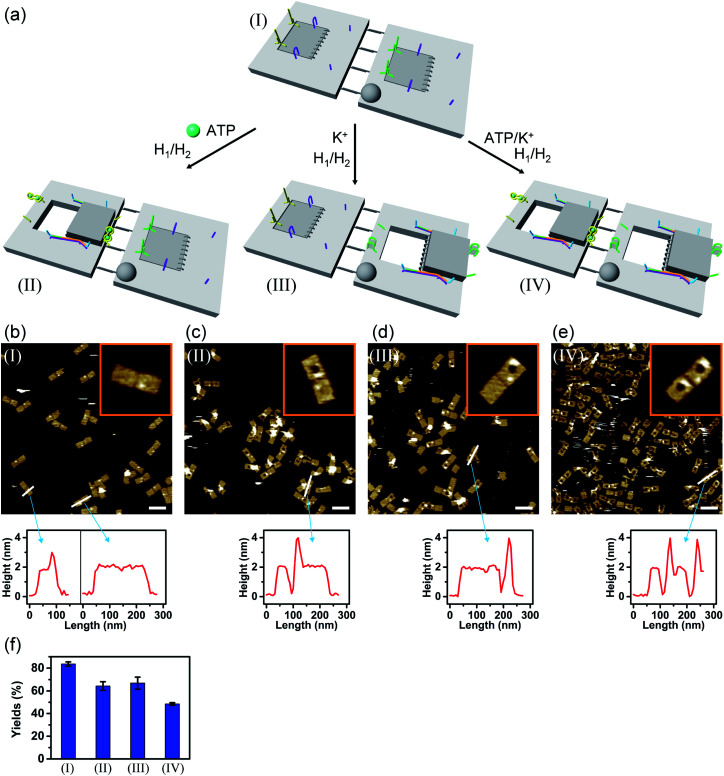
(a) Programmed triggered unlocking of nanocavities in an origami dimer-tile structure using ATP and K^+^ ions as unlocking triggers: treatment of dimer I with ATP and H_1_/H_2_ unlocks the ATP-responsive origami tile leading to the nanocavity-containing dimer in structure II. Subjecting dimer I to K^+^ ions and H_1_/H_2_ leads to the unlocking of the G-quadruplex-responsive tile and to the formation of the nanocavity-containing dimer in structure III. Treatment of origami dimer I with the two triggers ATP and K^+^ ions and H_1_/H_2_ yields the double nanocavity-containing origami dimer, structure IV. (b) AFM image corresponding to dimer I and the respective cross-sectional analysis. (c), (d) and (e) AFM images corresponding to the origami dimer structures and the respective cross-section analysis of the origami dimer structures, II, III and IV. (b–e), Scale bars: 200 nm. (f) Yields of origami structures corresponding to closed origami dimer I and to the nanocavity-containing origami structures II, III and IV generated by the respective triggers.

In the next step, the dimerization of origami tiles and the programmed unlocking of “holes” in the dimer structures were examined; Fig. 5. In this experiment, the origami tiles were locked by the ATP–aptamer stimuli- and G-quadruplex-responsive locks, and the tiles were interlinked through twelve duplex bridges (the schemes show only four of these bridges). The G-quadruplex-responsive tile was labeled with a four-hairpin marker for identification. Treatment of the integrated locked dimer origami structure, configuration I, with ATP and the “helper” strands H_1_/H_2_ leads to unlocking of the ATP-responsive tile and to the selective formation of the cavity in the respective tile, configuration II. Subjecting the integrated dimer, configuration I, to K^+^ ions and the strands H_1_/H_2_ leads to unlocking of the G-quadruplex-responsive units and to the formation of the cavity in the “marker”-labeled tile, configuration III. Treatment of the origami dimer, configuration I, with K^+^ ions and ATP and the strands H_1_/H_2_ results in the unlocking of the two origami locking components and in the formation of the cavities in the two tiles, configuration IV. Fig. 5b–e show the AFM images corresponding to the programmed unlocking of the respective dimer origami structures. In Fig. 5b the AFM image shows the integrated dimer origami structures, configuration I. The cross-sectional analysis of each of the tiles shows, in one tile, the characteristic height of the origami raft, 2 nm, and in the second tile a “spike” of *ca.* 1 nm, corresponding to the “marker” on the height of the base raft origami structure, is observed (see also the inset). In Fig. 5c, the image of the dimers generated upon treatment of the integrated origami dimer, configuration I, with ATP and H_1_/H_2_ is presented. Clearly, the cavities are generated only in the ATP-responsive origami tiles. The “marker”-functionalized tiles remain as integrated “locked” structures, configuration II (see the inset and cross-sectional analysis of the tiles). Fig. 5d shows the AFM image of the K^+^ ions and H_1_/H_2_ treated dimers. The marker-functionalized, G-quadruplex-responsive tiles are unlocked, while the ATP-responsive tiles retain the locked integrated structures, configuration III (see the inset, Fig. 5d). That is, the selective unlocking of the G-quadruplex-responsive tiles and the formation of the nanocavities in these tiles are observed. Fig. 5e depicts the AFM image of the nanohole-modified dimer tiles formed upon the treatment of the origami dimers in configuration I with the two triggers, ATP and K^+^ ions, and the hairpins H_1_/H_2_. The cavities are formed in the two tiles, configuration IV. This is supported by the observation of the two holes in the two tiles (*cf.*Fig. 5e, inset, and appropriate cross-sectional analysis). The yields of unlocked configurations II, III and IV, upon the analysis of *N* = 4 large-area domains, corresponded to 60%, 65% and 45%, respectively (Fig. 5f, S27–S30 and Tables S22–S25†).

**Fig. 6:**
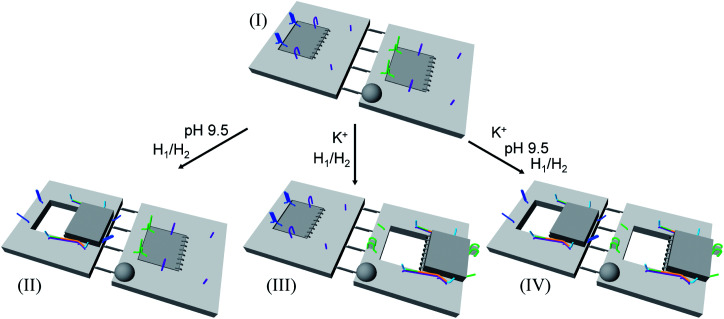
Schematic programmed triggered formation of nanocavities in origami-dimer structures using pH and K^+^ ions as unlocking triggers: subjecting the dimer in configuration I to pH = 9.5 and H_1_/H_2_ yields the nanocavity-modified dimer, configuration II, where the cavity is generated in the origami tile without the hairpin-marker. Treatment of the dimer, configuration I, with K^+^ ions and H_1_/H_2_ leads to the nanocavity-functionalized dimer, configuration III, where the cavity is generated in the hairpin-marked origami tile. Subjecting the origami dimer, configuration I, to K^+^ ions and H_1_/H_2_ at pH = 9.5 leads to the origami dimer that contains nanocavities in the two tiles.

In addition, the multiplexed triggered programmed unlocking of the T-A·T pH-responsive and the K^+^-ion-stabilized G-quadruplex-responsive origami dimer tiles was demonstrated; Fig. 6. Subjecting the dimer origami structure, configuration I, to pH = 9.5 dissociates the T-A·T triplexes and pH-responsive locks, and in the presence of the “helper” hairpins H_1_/H_2_ the respective cavity is unlocked giving rise to the dimer in configuration II. Treatment of the dimer, configuration I, with K^+^ ions unlocks, in the presence of H_1_ and H_2_, the marked tile, resulting in the generation of the cavity-containing dimer in configuration III. The interaction of the dimer in configuration I with K^+^ ions at pH = 9.5, results in the unlocking of the patches in the two tiles and the formation of nanocavities in the two tiles. The experimental results demonstrating the programmed unlocking of the cavities in the dimer origami composed of the pH-responsive raft and the K^+^-ion/CE responsive raft are presented in Fig. S31, ESI. Fig. S31a–d† confirm the triggered formation of the respective cavity-functionalized dimers. The dimer in configuration I is characterized by tiles of height 2 nm, where one of the tiles reveals a spike (*ca.* 0.9 nm) corresponding to the hairpin-marker on one of the tiles (inset Fig. S31a,† and cross-sectional analysis). Treatment of the dimer at pH = 9.5 shows the formation of the cavity in the non-marked tile, while the G-quadruplex-responsive tile stays locked, consistent with configuration II (Fig. S31b,† inset, and respective cross-sectional analysis). Treatment of the dimer, configuration I, with K^+^ ions shows the formation of the cavity in the tile with the hairpin-marker, while the pH-responsive tile stays locked, consistent with configuration III (Fig. S31c,† inset, and respective cross-sectional analysis). Treatment of the dimer, configuration I, with K^+^ ions, at pH = 9.5 leads to the generation of two nanocavities in the origami dimer (Fig. S31d† and cross-sectional profile). The yields of the triggered cavity-containing dimer origami structures II, III and IV correspond to 60%, 60% and 45%, respectively (Fig. S31e, S32–S35 and Tables S26–S29†) (the yields and error bars are obtained by analysing four large AFM scanning domains).

**Fig. 7:**
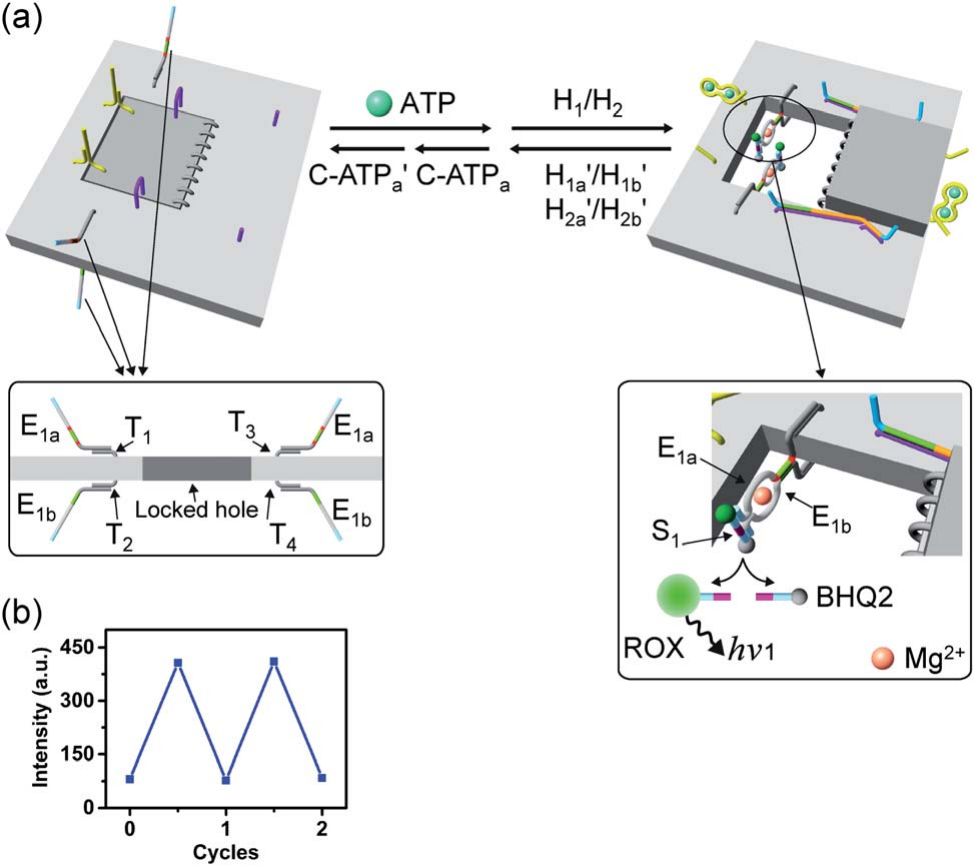
(a) The ATP-triggered opening of a nanocavity in the origami raft and the activation of Mg^2+^-ion-dependent DNAzyme in the resulting confined nanohole generated in the origami tile. (b) Reversible “ON” and “OFF” operation of the Mg^2+^-ion-dependent DNAzyme in the confined nanocavity upon the triggered ATP-stimulated opening of the nanocavity in the presence of ATP/H_1_/H_2_ and the closure of the nanocavity in the presence of counter agents, separating the ATP/aptamer complexes.

A major goal involves, however, the identification of possible functional applications of the “mechanically” generated cavities in the origami rafts and, particularly, the design of programmed functionalities in the triggered formed cavities. The operation of catalytic transformations in confined nano-environments has attracted growing interest recently.^41,42^ The concentration of catalyst subunits or catalytic units in confined nanostructures or nano-environments provides a general means to assemble effective catalytic modules or to operate biocatalytic cascades. Thus, the triggered opening and closure of the nanocavities could provide a means to switch catalytic transformations within the cavities. This is exemplified in Fig. 7a with the functionalization of the locked ATP-responsive origami raft with four toehold tethers T_1_, T_2_, T_3_ and T_4_. The tethers T_1_ and T_3_ protrude from the upper plane of the origami raft, whereas the tethers T_2_ and T_4_ protrude from the counter plane of the origami raft. The strands E_1a_ and E_1b_ are hybridized with the tethers T_1_:T_3_ and T_2_:T_4_, respectively. The ATP-stimulated unlocking of the cavities in the rafts, in the presence of ATP and the “helper” strands H_1_/H_2_, results in the assembly of the Mg^2+^-ion-dependent DNAzyme in the cavity. As the substrate S_1_ is modified with the ROX-fluorophore/BHQ2-quencher, the cleavage of the substrate leads to the triggered fluorescence of ROX, *λ* = 608 nm. By subjecting the open-cavity catalytic origami system to the counter-helper strands 
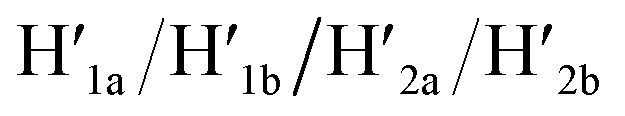
 and the counter-ATP–aptamer strand, C-ATP_a_, and centrifugation, and subsequently to the strand displacer 
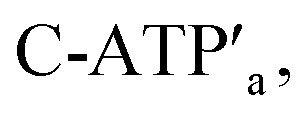
 the locking of the cavities proceeds, resulting in the switched-off catalytic state of the system. The re-treatment of the origami tiles with ATP, the “helper units” H_1_/H_2_, and the substrate S_1_ resulted in the re-opening of the cavities and the re-activation of the catalytic activity of the DNAzyme in the cavity (details in Fig. S36†). Fig. 7b shows the switchable catalytic activities of the DNAzyme in the cavity (details in Fig. S37†). Using a similar concept, the K^+^-ion-stabilized G-quadruplex-responsive tiles were functionalized with E_2a_ and E_2b_ that yielded, in the presence of K^+^ ions and H_1_/H_2_, the in-cavity assembly of a second Mg^2+^-dependent DNAzyme that selectively cleaves the substrate S_2_ modified with the fluorophore Cy5 and the quencher BHQ2, leading to the fluorescence of Cy5, *λ* = 665 nm, Fig. S38 and S39.† In addition, the functionalization of the T-A·T pH-responsive origami rafts with E_3a_ and E_3b_ led, at pH = 9.5 and in the presence of the “helper” strands H_1_/H_2_, to the unlocking of the tiles and to the in-cavity assembly of a third Mg^2+^-ion-dependent DNAzyme that cleaves the substrate S_3_ modified with the FAM fluorophore and the BHQ1-quencher, Fig. S40 and S41.† The cleavage of the substrate S_3_ led to the switched-on fluorescence of FAM, *λ* = 518 nm.

**Fig. 8:**
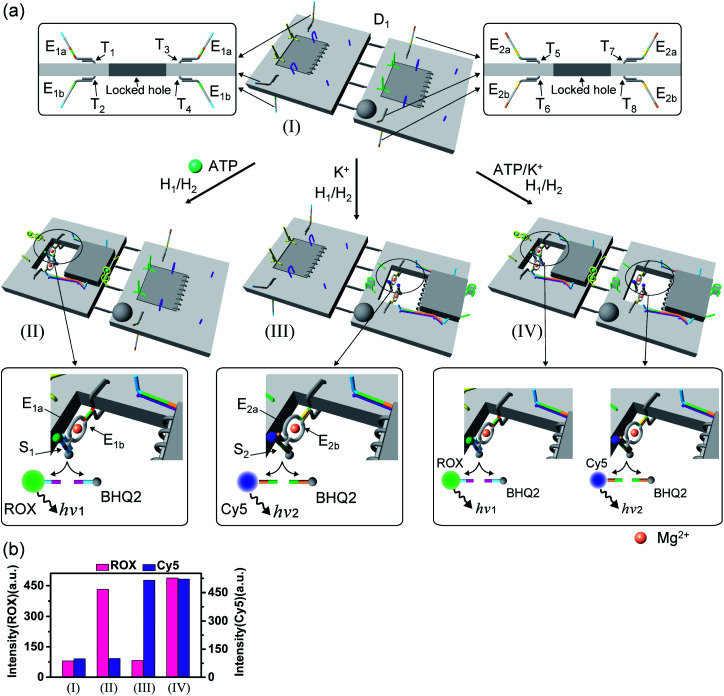
(a) Programmed triggered catalytic transformations within ATP-/K^+^-ion-responsive origami dimer nanostructures: treatment of the locked dimer D_1_ in configuration I with ATP and H_1_/H_2_ leads to the unlocking of the ATP-responsive origami tile and to the formation of dimer in configuration II. The self-organization of E_1a_/E_1b_ tethers into the Mg^2+^-ion-dependent DNAzyme leads to the cleavage of substrate S_1_ resulting in the fluorescence of ROX. Subjecting the dimer D_1_, configuration I, to K^+^ ions and H_1_/H_2_ results in the selective formation of nanocavities in the K^+^-ion-responsive origami tiles, configuration III, where the assembly of the tethers E_2a_/E_2b_ in the unlocked cavity leads to the formation of a second Mg^2+^-ion-dependent DNAzyme that cleaves substrate S_2_ resulting in the fluorescence of Cy5. Treatment of the locked dimer D_1_ in configuration I with ATP and K^+^ ions and H_1_/H_2_ leads to the unlocking of both tiles and to the activation of both Mg^2+^-ion-dependent DNAzymes in the two confined nanocavities, configuration IV, that lead to the cleavage of the substrates S_1_ and S_2_ and to the fluorescence of ROX and Cy5. (b) The fluorescence intensities of the ATP-/K^+^-ion-responsive dimer upon the programmed triggered unlocking of the dimer D_1_, configuration I, in the presence of the OR/AND ATP/K^+^-ion triggers, resulting in the guided DNAzyme-catalyzed cleavage of S_1_ or S_2_ in the confined nanocavities.

The engineering of three different DNAzyme functionalities in the nanocavities allowed, then, the programmed triggered operation of different catalytic transformations within the “mechanically” engineered confined cavities in origami dimer rafts. Fig. 8 depicts the triggered programmed catalytic functions driven in the ATP-/G-quadruplex-responsive origami dimer structure. The ATP-responsive tiles, functionalized with the E_1a_/E_1b_ DNAzyme subunits, were linked to the G-quadruplex-responsive tiles, modified with the E_2a_/E_2b_ DNAzyme subunits, using twelve-duplex linkers to yield the dimer D_1_ in the closed configurations, state I. In the presence of ATP and H_1_/H_2_, the ATP-responsive tiles are opened, resulting in the switching “ON” of the Mg^2+^-ion-dependent DNAzyme that cleaves the ROX/BHQ2-modified substrate S_1_, state II. Under these conditions, the cleavage of S_1_ is activated, reflected by the switched “ON” luminescence of ROX (*λ* = 608 nm) and the switched “OFF” catalytic functions of the G-quadruplex-responsive tile. Treatment of the dimer tile D_1_ in state I with K^+^ ions and H_1_/H_2_ leads to the selective unlocking of the G-quadruplex-responsive tile and to the selective activation of the Mg^2+^-ion-dependent DNAzyme that cleaves substrate S_2_, modified with the Cy5/BHQ2-functionalities, resulting in the fluorescence of Cy5, *λ* = 665 nm, state III. Finally, treatment of the dimer in state I with ATP and K^+^ ions as triggers, and H_1_/H_2_, unlocks the two origami rafts leading to the functional cavities that induce the activation of the respective E_1a_/E_1b_ Mg^2+^-ion-dependent DNAzyme and the E_2a_/E_2b_ Mg^2+^-ion-dependent DNAzyme in the respective cavities, leading to the cleavage of S_1_ and S_2_ and the formation of the luminescence of ROX and Cy5, respectively, state IV. Fig. 8b depicts the programmed luminescence properties of the systems in the presence of the respective triggers. In the absence of the triggers, the closed-cavity configuration of the dimer origami raft D_1_ shows only the background luminescence of the substrates S_1_ and S_2_ (details in Fig. S42†).

**Fig. 9:**
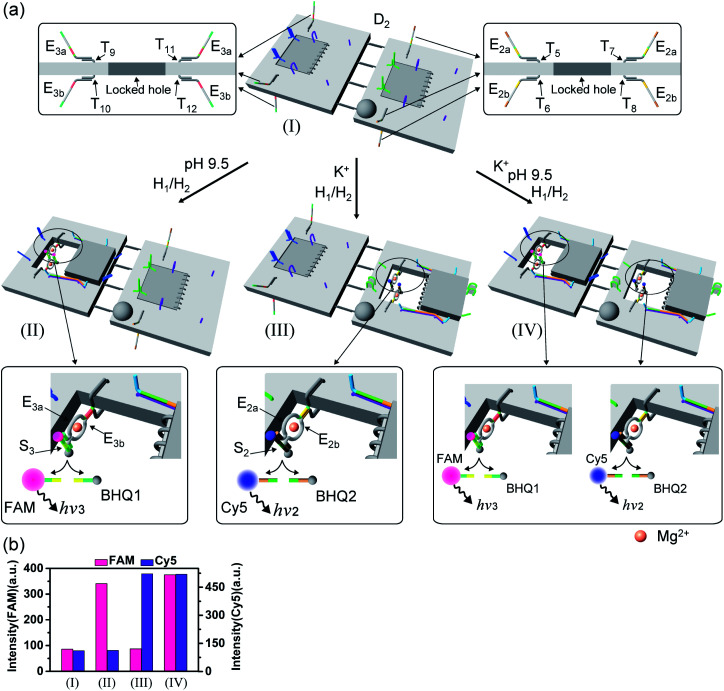
(a) Programmed triggered DNAzyme-stimulated transformations within pH-/K^+^-ion-responsive origami dimer nanostructures: treatment of the origami dimer tile D_2_, state I, at pH = 9.5, in the presence of the “helper” strands, H_1_, H_2_, leads to the unlocking of the T-A·T locks and to the formation of the nanocavity-containing dimer, state II. The assembly of the E_3a_/E_3b_ subunits into the Mg^2+^-ion-dependent DNAzyme in the confined nanocavity leads to the hydrolytic cleavage of substrate S_3_ and to the switched “ON” fluorescence of FAM (*λ* = 518 nm). Subjecting the dimer D_2_, state I, to K^+^ ions and H_1_/H_2_ results in the dictated unlocking of the K^+^-ion-responsive tile and to the formation of the nanocavity-containing tile in state III. Under these conditions the E_2a_/E_2b_ tethers are assembled in the nanocavity with the catalytically active Mg^2+^-ion-dependent DNAzyme fluorescence of Cy5 (*λ* = 665 nm). Treatment of the dimer origami D_2_, state I, with K^+^ ions at pH = 9.5, in the presence of the “helper” strands H_1_/H_2_ leads to the formation of the nanocavities in the two origami rafts and to the activation of the two Mg^2+^-ion-dependent DNAzymes in the nanocavities, respectively, state IV. This leads to the concomitant cleavage of the two substrates S_3_ and S_2_ and to the switched-ON fluorescence at *λ* = 518 nm and *λ* = 665 nm. (b) Fluorescence intensities of the fluorophores FAM and Cy5 generated by the respective DNAzymes in the origami dimer structures in states I, II, III and IV.

Fig. 9 depicts the triggered programmed catalytic functions driven in the pH-/K^+^-ion-stabilized G-quadruplex-responsive origami dimer structures. The dimer was composed of the pH-responsive tile and the G-quadruplex-responsive tile, functionalized with the E_3a_/E_3b_ DNAzyme subunits and the E_2a_/E_2b_ DNAzyme subunits, respectively, linked by twelve-duplex linkers to yield the closed configuration, state I. At pH = 9.5, the pH-responsive tiles are opened in the presence of hairpins H_1_/H_2_, resulting in the switching “ON” of the Mg^2+^-ion-dependent DNAzyme E_3a_/E_3b_ that cleaves the substrate S_3_ modified with FAM/BHQ1, state II. Under these conditions, the cleavage of S_3_ is activated, reflected by the switched “ON” luminescence of FAM (*λ* = 518 nm) and the switched “OFF” catalytic functions of the K^+^-ion-responsive tile. Treatment of the dimer tile D_2_, in state I, with K^+^ ions and H_1_/H_2_ leads to the selective activation of the catalytic functions of the K^+^-ion-responsive tile. The K^+^-ion-triggered unlocking of the tile leads to the selective activation of the Mg^2+^-ion-dependent DNAzyme that cleaves the Cy5/BHQ2-modified substrate S_2_, resulting in the fluorescence of Cy5, *λ* = 665 nm, state III. Finally, treatment of the dimer D_2_ in state I with pH = 9.5 and K^+^ ions as triggers, and H_1_/H_2_, unlocks the two origami rafts leading to the functional nanocavities that induce the activation of the respective E_3a_/E_3b_ Mg^2+^-ion-dependent DNAzyme and the E_2a_/E_2b_ Mg^2+^-ion-dependent DNAzyme in the respective cavities, leading to the cleavage of S_3_ and S_2_ and the formation of the luminescence of FAM and Cy5, respectively, state IV. Fig. 9b depicts the programmed luminescence properties of the systems in the presence of the respective triggers. In the absence of the triggers, the closed-cavity configuration of the dimer origami raft D_2_ shows only the background luminescence of the substrates S_3_ and S_2_ (details in Fig. S43†).

## Conclusions

The present study has introduced versatile means to yield “mechanically” reversibly triggered nanocavities in open and closed configurations in origami rafts. The K^+^-ion-stabilized G-quadruplexes, the assembly of ligand–aptamer complexes, and the pH-stimulated dissociation of T-A·T triplexes were used as functional units to “mechanically” unlock the origami structures to yield nanocavities, *ca.* 20 nm in diameter, in the origami rafts. By applying counter triggers, *e.g.*, crown-ether separating the K^+^-ion-stabilized G-quadruplexes, strands separating the ligand–aptamer complexes, or pH-conditions favoring T-A·T triplex structures, the nanocavities could be re-locked. By the conjugation of origami tiles into dimer structures, the programmed “mechanical” formation of nanocavities in the presence of one or two triggers was demonstrated. This concept may be extended to yield supramolecular origami-structures of enhanced complexities, *e.g.*, trimers or tetramers, revealing programmable triggered “mechanical” generation of nanocavities. The mechanically generated nanocavities provided confined nano-environments for the reversible activation of catalytic transformations in the confined volumes. Specifically, by the tethering of DNAzyme subunits on the upper and counter faces of the origami scaffolds, the triggered unlocking of the nanocavities allowed the self-assembly of the active DNAzyme in the confined nano-environment. By the reversible “mechanically” triggered opening and closure of the nanocavities, the switchable “ON”/“OFF” operation of the DNAzymes was demonstrated. In addition, the multi-triggered programmed opening of nanocavities in origami scaffolds enabled the guided, dictated activation of different DNAzymes in the respective confined nano-environments. These results call for practical applications of nanocavity containing origami scaffolds, particularly in the area of “smart” nanomedicine. As the origami raft reveals intracellular permeation,^43^ the tethering of ribozyme subunits on counter faces of the origami tiles and the design of biomarker-responsive locks are envisaged to yield “smart” functional therapeutic carriers. That is, the biomarker-induced unlocking of the cavities could assemble the active ribozyme in the nanocavity for gene manipulation.^44^

## Conflicts of interest

There are no conflicts to declare.

## Supplementary Material

SC-012-D0SC04108D-s001
