# Supplementary material for: Programmed catalysis within stimuli-responsive mechanically unlocked nanocavities in DNA origami tiles
Source: Chem Sci. 2020 Oct 27;12(1):341–51. doi: 10.1039/d0sc04108d (PMC8179671; doi:10.1039/d0sc04108d)
Supplement: SC-012-D0SC04108D-s001 [file SC-012-D0SC04108D-s001.pdf]

Electronic Supplementary Material (ESI) for Chemical Science.
This journal is © The Royal Society of Chemistry 2020

Supporting Information

Programmed Catalysis Within Stimuli-Responsive Mechanically Unlocked Nanocavities in DNA Origami Tiles

*Jianbang Wang, Zhixin Zhou, Zhenzhen Li and Itamar Willner**

Institute of Chemistry, The Center for Nanoscience and Nanotechnology,
The Hebrew University of Jerusalem, Jerusalem 91904, Israel

Experimental Section

Preparations of DNA origami tiles and hairpin strands. The DNA origami tiles were assembled in TAE buffer (20 mM Tris; 20 mM acetic acid; 1 mM EDTA; pH 8.0) with 12.5 mM Mg^{2+} using 10 nM single-stranded M13mp18 phage DNA (New England Biolabs) and 100 nM short staple strands (unmodified staple strands and functional staple strands were custom-ordered, Integrated DNA Technologies). The mixture was heated to 95 °C in a thermal cycler and then allowed to cool down to 20 °C at a rate of 0.1 °C/10 s. The DNA origami tiles were purified using agarose electrophoresis (1%, 80 V, 2 h, at 0 °C) to remove the excess staple strands and were then extracted from the gel bands using Freeze 'N Squeeze spin columns. For the origami dimers, two tiles were mixed at 1:1 molar ratio and kept at 25 °C for 12 h. The hairpin strands (5 μM) were annealed from 90 °C to 10 °C at a rate of 3 °C /min in TAE buffer with 6 mM Mg^{2+} and 5 mM Na^{+}.

Formation of nanocavities in DNA origami tiles. To unlock the purified K^{+}-ion-responsive tiles (2 nM, 80 μL, in TAE buffer with 6 mM Mg^{2+} and 5 mM Na^{+}), K^{+} ions (1 M, 10 μL) were applied in the presence of the hairpin strands H_1 and H_2 (100 nM, 10 μL) and the sample was allowed to react for 10 hours at 25 °C. For the reverse process to re-lock the cavities, the anti-helper strands ($H_{1a}'/H_{1b}'/H_{2a}'/H_{2b}'$) (500 nM, 10 μL) were added to the sample to remove the strands H_1 and H_2 (25 °C for 2 h) and crown-ether (CE, 55 mM, 900 μL) was used to separate the G-quadruplex by removing the K^{+} ions. The sample was centrifuged four times to remove the excess helpers/anti-helpers and CE (100 k NMWL, 3000 × g, 10 min). For the control measurements, only the K^{+} ions or the helper hairpin strands H_1/H_2 were added into the origami solutions.

For the FRET test, the strand $H_{a\text{-}F}$, internally modified with Cy3, and the anchor strand $A_{1\text{-}F}$ modified with Cy5 were used for preparation of the K^{+}-ion-responsive origami tile. The fluorescence features of the locked configuration of the origami tiles, 24 nM, were measured at λ_{ex} = 532 nm. Subsequently, the origami tiles were unlocked by K^{+} ions and the added hairpins H_1 and H_2. The fluorescence spectrum of the origami tiles in the open cavity-containing configuration was measured (λ_{ex} = 532 nm). For deriving the calibration curve, mixtures of the strands $H_{a\text{-}F}$ (24 nM) and $A_{1\text{-}F}$ (24 nM) were subjected to variable concentrations of $H_{1\text{-}F}$ (0, 6, 12, 18, and 24 nM) and their fluorescence spectra of the resulting hybrids were measured (λ_{ex} = 532 nm).

To unlock the ATP-responsive origami tiles (2 nM, 80 μL, in TAE buffer with 6 mM Mg^{2+} and 5 mM Na^{+}), the sample was treated with ATP (50 mM, 10 μL) and the helper strands H_1 and H_2 (100 nM, 10 μL) allowed to react for 10 hours at 25 °C. For the reverse re-locking process, the anti-helper strands ($H_{1a}'/H_{1b}'/H_{2a}'/H_{2b}'$) (500 nM, 10 μL) were added into the sample to remove the strands H_1 and H_2 (25 °C for 2 h) and the counter-ATP aptamer strand C-ATP$_a$ (200 nM, 10 μL) was added to remove ATP from the ATP-aptamer complex followed by centrifuging to remove the free ATP from the sample (100 k NMWL, 3000 × g, 10 min, four times). Then the anti-C-ATP$_a$ strand, C-ATP$_a$' (1 μM, 10 μL) was applied to remove C-ATP$_a$ and to form the M/M' locking duplex units of the "window". For the control measurements, only the ATP or the helper

hairpin strands H_1/H_2 were used.

To unlock the pH-responsive origami tiles (2 nM, 80 μL, in TAE buffer with 6 mM Mg^{2+} and 5 mM Na^+), the sample was subjected to the helper strands H_1 and H_2 (50 nM, 20 μL) at pH = 9.5 and allowed to react for 10 hours at 25 °C. For the reverse re-locking process, the anti-helper strands ($H_{1a}'/H_{1b}'/H_{2a}'/H_{2b}'$) (500 nM, 10 μL) were added into the sample to remove the strands H_1 and H_2 (25 °C for 2 h) at pH 6. For the control measurements, the origami tiles were treated at pH = 9.5 or the helper strands H_1/H_2.

Programmed Formation of nanocavities in the ATP-/K^+-ion-responsive origami dimers. The dimer mixture composed of ATP-responsive (without marker) and K^+-ion-responsive (with four-hairpin labeled marker) tiles (state I, 2 nM, 80 μL, in TAE buffer with 6 mM Mg^{2+} and 5 mM Na^+) were prepared. The ATP (50 mM, 2.5 μL) and helper hairpin strands, H_1/H_2 (100 nM, 2.5 μL), were used to unlock the unmarked tile of the dimer (state I, 2 nM, 20 μL), state II. To unlock state I of the dimer mixture to state III, K^+ ions (1 M, 2.5 μL) and the helper hairpin strands, H_1/H_2 (100 nM, 2.5 μL), were added to the tiles mixture in state I (2 nM, 20 μL). For unlocking the nanocavities in both tiles of the dimer (state I, 2 nM, 20 μL) into state IV, ATP (50 mM, 3 μL), K^+ ions (1 M, 3 μL) and the helper strands H_1/H2 (150 nM, 4 μL) were added into the sample to yield the two-cavity-containing dimers in state IV.

Programmed generation of the nanocavities in the pH-/K^+-ion-responsive origami dimers. For the generation of the nanocavities in the unmarked pH-responsive tile, the dimers in state I (2 nM in 20 μL of TAE buffer that included 6 mM Mg^{2+} ions and 5 mM Na^+ ions) were treated with helper hairpin strands, H_1/H_2 (50 nM, 5 μL) at pH = 9.5 to unlock the unmarked pH-responsive tile to yield state II. To generate the nanocavities in the marked G-qudruplex-responsive tile, the mixture in state I (2 nM, 20 μL) was treated with K^+ ions (1 M, 2.5 μL) and the helper strands, H_1/H_2 (100 nM, 2.5 μL) to unlock the cavities and yield state III. For opening the nanocavities in the two tiles (state I, 2 nM, 20 μL) was treated with K^+ ions (1 M, 2.5 μL) and the helper strands H_1 and H_2 solution (200 nM, 2.5 μL) at pH = 9.5. The mixture was allowed to react for 10 hours at 25 °C to yield state IV.

Switchable catalysis in the nanocavities of the single DNA origami tiles. For switching the catalytic process in ATP-responsive tiles, the ATP-responsive origami tiles functionalized with the E_{1a} and E_{1b} were prepared (30 nM, 200 μL). To induce the opening of the nanocavities and to activate the Mg^{2+}-ion-dependent DNAzyme in the nanocavity, the tiles were treated with the ATP (60 mM, 20 μL) and the helper hairpins H_1/H_2 (1.5 μM, 20 μL) for 10 hours at 25 °C. To a sample, 49 μL of 15 nM, of the open-cavity origami tiles was added with the ROX/BHQ2-modified substrate S_1 (100 μM, 1 μL) and the catalytic activity of the DNAzyme was probed by following the fluorescence of the ROX modified fragmented product (λ_{ex} = 550 nm). To reclose the open cavity tiles, the system was treated with the blocker strands B_{1a}/B_{1b} (3 μM, 10 μL) and the system was allowed to anneal from 30 °C to 10 °C for 2 hours. Subsequently, the anti-helper strands, $H_{1a}'/H_{1b}'/H_{2a}'/H_{2b}'$ (15 μM, 10 μL) and C-ATP_a (6 μM, 10 μL) were added to the system that was allowed to react for 2 hours. The resulting tiles were

four times centrifuged to remove the free ATP from the sample (100 k NMWL, 3000 × g, 10 min), and the strand C-ATP$_a$' (12 μM, 10 μL) and the anti-blocker strands B_{1a}'/B_{1b}' (10 μL, 6 μM) were added to the system to relock the cavities to yield the initial state tiles. The catalytic functions of the system were checked as described above. The re-opening and reclosure of the cavities were performed by repeating the cycle and probing the catalytic activities of the system in the open/closed nanocavity states.

For switching the catalytic process in K^+-ion-responsive tiles, the origami tiles functionalized with the E_{2a} and E_{2b} were prepared (30 nM, 200 μL). The tiles were treated with the K^+ ions (1.2 M, 20 μL) and the helper hairpins H_1/H_2 (1.5 μM, 20 μL) for 10 hours at 25 °C. To a sample, 49 μL of 15 nM, of the open-cavity origami tiles was added with the Cy5/BHQ2-modified substrate S_2 (100 μM, 1 μL) and the catalytic activity of the DNAzyme was probed by following the fluorescence of the Cy5 modified fragmented product (λ_{ex} = 630 nm). To reclose the open cavity tiles, the system was treated with the blocker strands B_{2a}/B_{2b} (1 μM, 30 μL) and the system was allowed to anneal from 30 °C to 10 °C for 2 hours. Subsequently, the anti-helper strands, $H_{1a}'/H_{1b}'/H_{2a}'/H_{2b}'$ (5 μM, 30 μL) and crown-ether (CE, 57 mM, 2100 μL) were added to the system that was allowed to react for 2 hours at 25 °C to relock the cavities. The resulting tiles were washed and, centrifuged four times, to remove the excess strands and CE from the sample (100 k NMWL, 3000 × g, 10 min). The sample was treated with the anti-blocker strands B_{2a}'/B_{2b}' (30 μL, 2 μM) to regenerate the initial state. The catalytic functions of the system were examined as described above. The re-opening and reclosure of the cavities were performed by repeating the cycle and probing the catalytic activities of the system in the open/closed nanocavity states.

For switching the catalytic process by pH-responsive tiles, the origami tiles functionalized with the E_{3a} and E_{3b} were prepared (30 nM, 200 μL). To induce the opening of the nanocavities and to activate the Mg^{2+}-ion-dependent DNAzyme in the nanocavity, the tiles were treated with the helper hairpins H_1/H_2 (3 μM, 10 μL) for 10 hours at pH = 9.5. To a sample, 49 μL of 15 nM, of the open-cavity origami tiles was added the FAM/BHQ1-modified substrate S_3 (100 μM, 1 μL) and the catalytic activity of the DNAzyme was probed by following the fluorescence of the FAM modified fragmented product (λ_{ex} = 495 nm). To reclose the open cavity tiles, the system was treated with the blocker strands B_{3a}/B_{3b} (3 μM, 10 μL) and the system was allowed to anneal from 30 °C to 10 °C for 2 hours. Subsequently, the anti-helper strands, $H_{1a}'/H_{1b}'/H_{2a}'/H_{2b}'$ (15 μM, 10 μL) were added to the system that was allowed to react for 2 hours at 25 °C to relock the cavities at pH 6. The sample was subjected to the anti-blocker strands B_{3a}'/B_{3b}' (10 μL, 6 μM) to yield the initial state. The catalytic functions of the system were checked as described above. The re-opening and reclosure of the cavities were performed by repeating the cycle and probing the catalytic activities of the system in the open/closed nanocavity states.

Programmed catalytic activities in the nanocavities within the ATP-/K^+-ion-responsive origami dimers D_1. To induce the programmed transition of the E_{1a}/E_{1b} and E_{2a}/E_{2b}-functionalized ATP-/K^+-ion-responsive dimer D_1 into the catalytically active dimer in state II, the dimer in state I (100 μL, 30 nM in a TAE buffer that contained

Mg^{2+}, 6 mM, and Na^+, 5 mM) was subjected to ATP (10 μL, 60 mM) and the hairpin strands H_1/H_2 (10 μL, 1.5 μM), for a time interval of 10 hours, to unlock the nanocavities in the ATP-responsive tile, and assemble the Mg^{2+}-ion-dependent DNAzyme in the cavities. For the programmed opening of the K^+-ion-responsive tile (marked) in state I into state III, the dimer in state I (100 μL, 30 nM) was subjected to K^+ ions (10 μL, 1.2 M) and the hairpin strands H_1/H_2 (10 μL, 1.5 μM), for a time interval of 10 hours, to unlock the nanocavities in the K^+-ion-responsive tile, and assemble the Mg^{2+}-ion-dependent DNAzyme in the cavities. For the programmed unlocking of the nanocavities in the two tiles of state I (100 μL, 30 nM) into state IV, the respective steps detailed for unlocking the nanocavities in the ATP-responsive tile and the K^+-ion-responsive tile were applied. For the activation of the catalytic transformations in the different dimer configurations the origami mixtures were subjected to a mixture of the ROX/BHQ2-modified substrate S_1 (100 μM, 1 μL) and the Cy5/BHQ2-modified substrate S_2 (100 μM, 1 μL). The system were allowed to react for a time-interval of 6 hours at 25 °C. The fluorescence spectra of the resulting fluorophore-labeled fragments generated by the respective DNAzymes, associated with the different dimers were recorded (ROX, λ_{ex} = 550 nm; Cy5, λ_{ex} = 630 nm).

Programmed catalytic activities in the nanocavities within the pH-/K^+-ion-responsive origami dimers D_2. To induce the programmed transition of the E_{3a}/E_{3b} and E_{2a}/E_{2b}-functionalized pH-/K^+-ion-responsive dimer D_2 into the catalytically active dimer in state II, the dimer in state I (100 μL, 30 nM in a TAE buffer that contained Mg^{2+}, 6 Mm, and Na^+, 5 mM) was subjected to the hairpin strands H_1/H_2 (10 μL, 1.5 μM) at pH = 9.5, for a time interval of 10 hours, to unlock the nanocavities in the pH-responsive tile, and assemble the Mg^{2+}-ion-dependent DNAzyme in the cavities. For the programmed opening of the K^+-ion-responsive tile (marked) in state I into state III, the dimer in state I (100 μL, 30 nM) was subjected to K^+ ions (10 μL, 1.2 M) and the hairpin strands H_1/H_2 (10 μL, 1.5 μM), for a time interval of 10 hours, to unlock the nanocavities in the K^+-ion-responsive tile, and assemble the Mg^{2+}-ion-dependent DNAzyme in the cavities. For the programmed unlocking of the nanocavities in the two tiles of state I into state IV, the respective steps detailed for unlocking the nanocavities in the pH-responsive tile and the K^+-ion-responsive tile were applied. For the activation of the catalytic transformations in the different dimer configurations the origami mixtures were subjected to a mixture of the FAM/BHQ1-modified substrate S_3 (100 μM, 1 μL) and the Cy5/BHQ2-modified substrate S_2 (100 μM, 1 μL). The system were allowed to react for a time-interval of 6 hours at 25 °C. The fluorescence spectra of the resulting fluorophore-labeled fragments generated by the respective DNAzymes, associated with the different dimers were recorded (FAM, λ_{ex} = 495 nm; Cy5, λ_{ex} = 630 nm).

AFM imaging. For the AFM measurements, 2 μL of the respective origami samples were deposited on the freshly peeled mica. After adsorbing for 5 min, the samples were imaged in an aqueous buffer solution under tapping mode using SNL-10 probes (Bruker, Multimode Nanoscope VIII).

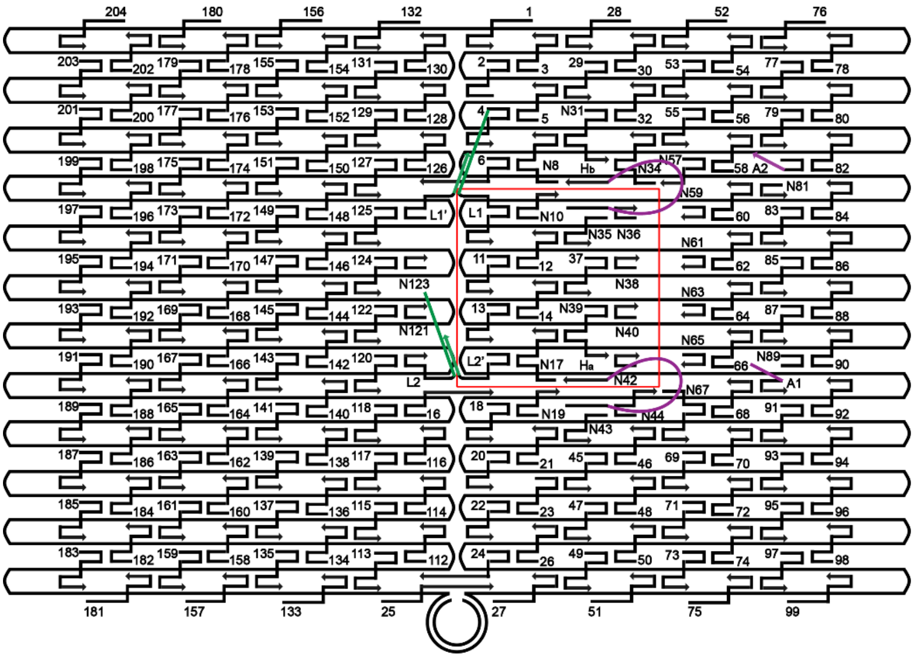

Figure S1. Schematic of the designed origami tile responsible to K^+-ion-stabilized G-quadruplexes and crown ether. The lock strands L1/L1' and L2/L2' shows in green, the handles (Ha, Hb) are purple loops and the anchoring tethers (A1, A2) are short purple lines. The red square shows the designed "nano-cavity" patch in the origami tile.

The "nano-cavity" is designed in the origami tile through opening an inner patch and binding to the nearby part of the origami tile. The patch is linked to the origami raft through the M13 strand (as hinges for its opening process) on its right side and the locks (green) on its left side. The lock strands L1 and L2 contain the G-rich sequences and form the G-quadruplexes in the presence of K^+ ions. The top and bottom sides of the designed "nano-cavity" have Ha and Hb handles (purple). One end of the handle is linked to the staple strand of the origami tile and the other end of the handle is linked to the staple of the "nano-cavity". A1 and A2 anchoring footholds are extending from the staple strands on the right side of the origami tile. The handles can hybridize with the helper strands to stabilize the path (in the form of Ha/H1/A1 and Hb/H2/A2).

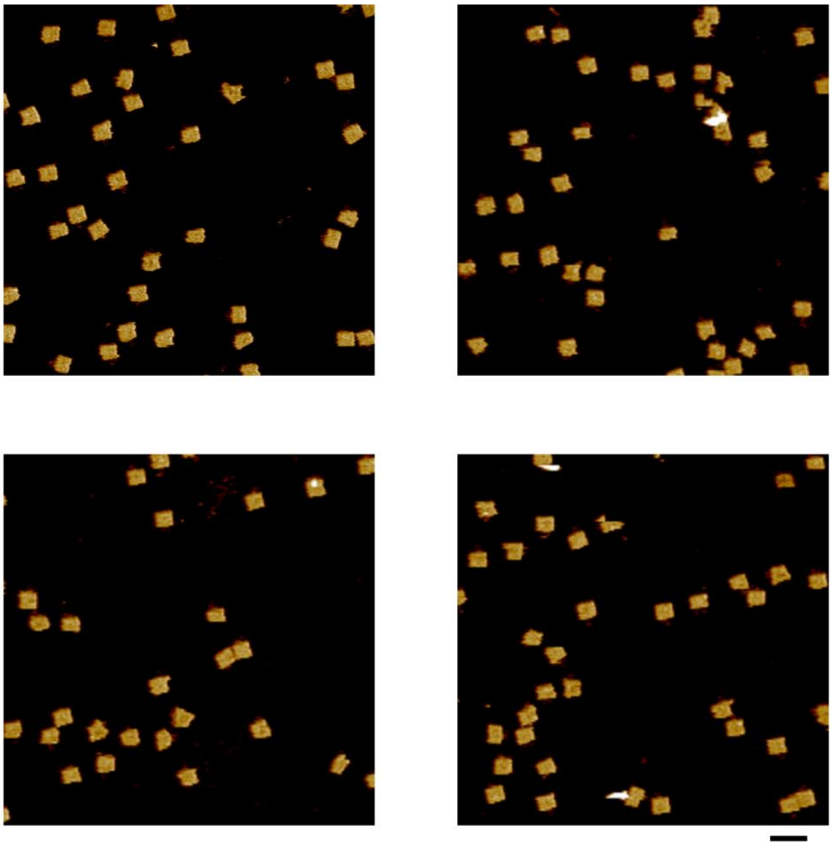

Figure S2. Four AFM images of the initial locked origami tiles before the treatment with K^+ ions and the helper hairpins H_1/H_2. Scale bar: 200 nm.

Table S1. Statistical analysis of the yields of the unlocked and locked origami tiles before the treatment with K^+ ions and the helper hairpins H_1/H_2.

**Statistical analysis**		**Unlocked**	**Locked**	**Incomplete structure**
**1**	Count	0	35	1
	Yield (%)	0	97.2	
**2**	Count	0	33	1
	Yield (%)	0	97.1	
**3**	Count	0	34	3
	Yield (%)	0	91.9	
**4**	Count	0	25	0
	Yield (%)	0	100	

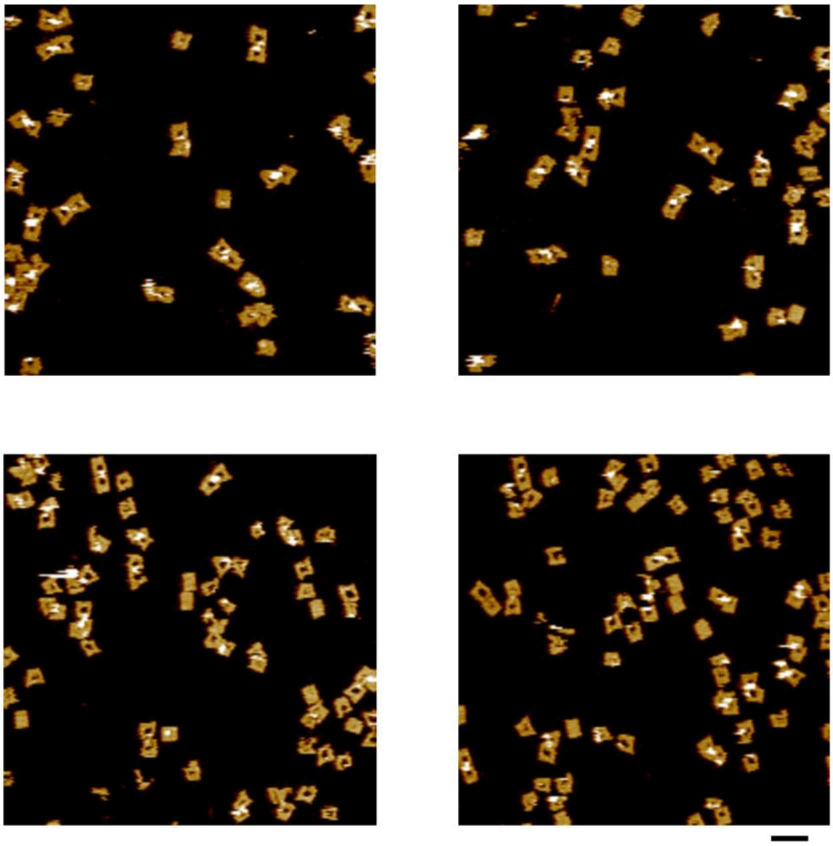

Figure S3. Four AFM images of the unlocked origami tiles upon the treatment of the tiles with K^+ ions and the helper hairpins H_1/H_2 (first cycle). Scale bar: 200 nm.

Table S2. Statistical analysis of the yields of the unlocked and locked origami tiles in the unlocked state after the treatment with K^+ ions and the helper hairpins H_1/H_2.

**Statistical analysis**		**Unlocked**	**Locked**	**Incomplete structure**
**1**	Count	27	5	2
	Yield (%)	79.4	14.7	
**2**	Count	26	6	2
	Yield (%)	76.5	17.6	
**3**	Count	42	15	5
	Yield (%)	67.7	24.2	
**4**	Count	36	12	4
	Yield (%)	69.2	23.1	

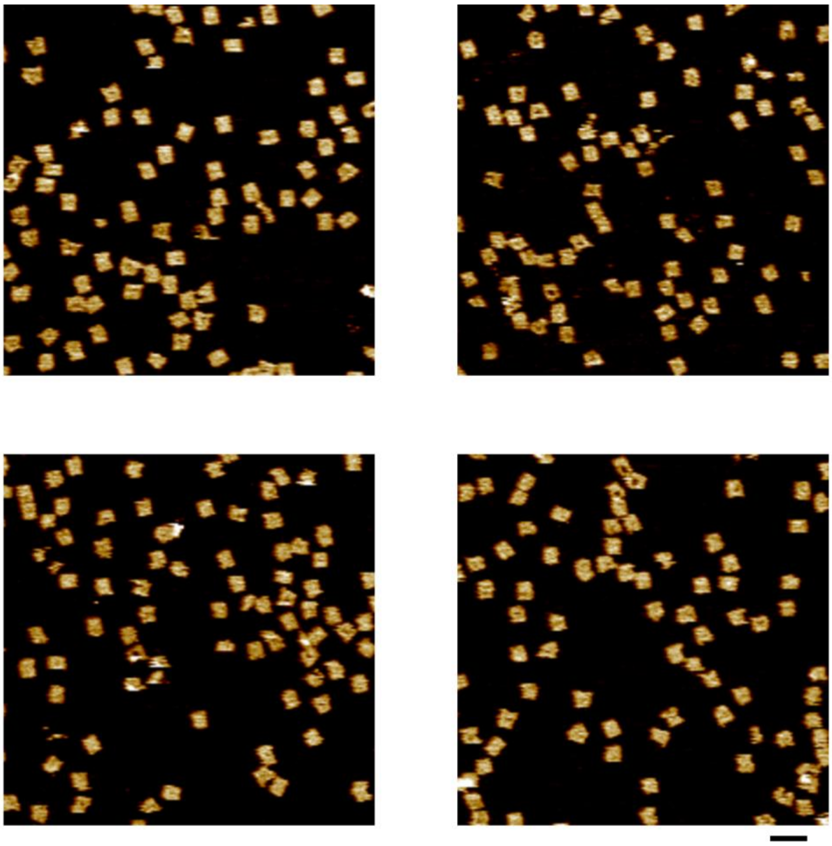

Figure S4. Four AFM images of the regenerated and locked origami tiles upon the treatment of the G-quadruplex unlocked tiles with anti-helper strands (H_{1a}'/H_{1b}' and H_{2a}'/H_{2b}') and crown ether (CE) (first cycle). Scale bar: 200 nm.

Table S3. Statistical analysis of the yields of the unlocked and locked origami tiles upon the treatment of the G-quadruplex unlocked tiles with the anti-helper strands (H_{1a}'/H_{1b}' and H_{2a}'/H_{2b}') and crown ether (CE).

**Statistical analysis**		**Unlocked**	**Locked**	**Incomplete structure**
**1**	Count	3	67	3
	Yield (%)	4.1	91.8	
**2**	Count	1	62	6
	Yield (%)	1.4	89.9	
**3**	Count	3	66	3
	Yield (%)	4.2	91.7	
**4**	Count	2	65	8
	Yield (%)	2.7	86.7	

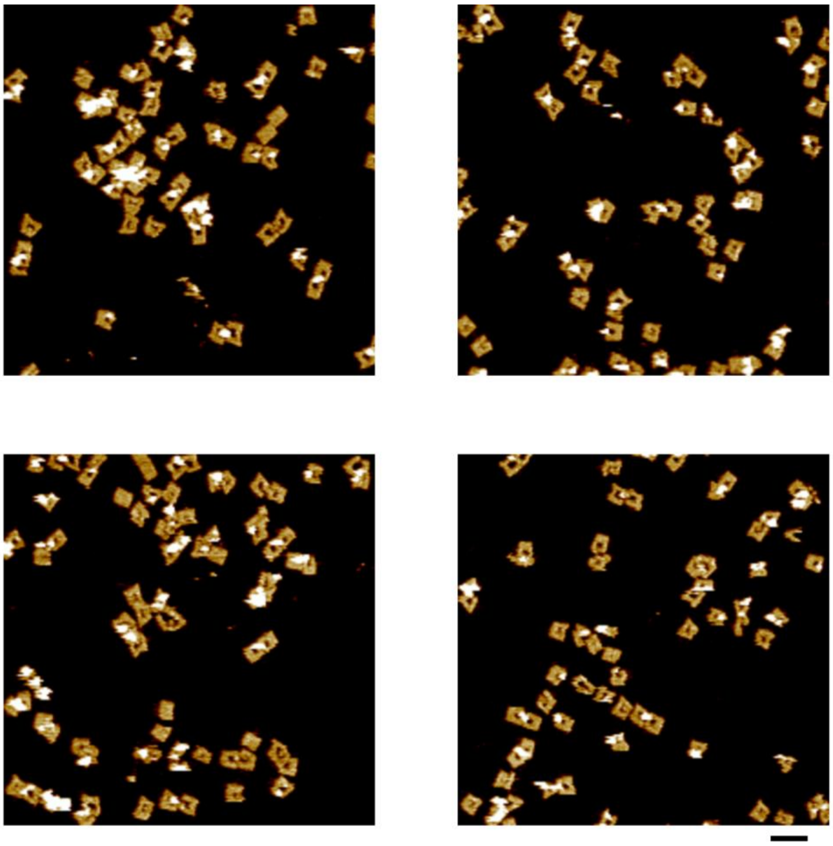

Figure S5. Four AFM images of the G-quadruplex unlocked origami tiles upon the treatment of the tiles with K^+ ions and the helper hairpins H_1/H_2 (second cycle). Scale bar: 200 nm.

Table S4. Statistical analysis of the yields of the unlocked and locked origami tiles upon the treatment of the locked tiles with K^+ ions and the helper hairpins H_1/H_2 (second cycle).

**Statistical analysis**		**Unlocked**	**Locked**	**Incomplete structure**
**1**	Count	37	9	2
	Yield (%)	77.1	18.8	
**2**	Count	39	9	2
	Yield (%)	78.0	18.0	
**3**	Count	34	8	5
	Yield (%)	72.3	17.0	
**4**	Count	38	12	5
	Yield (%)	69.1	21.8	

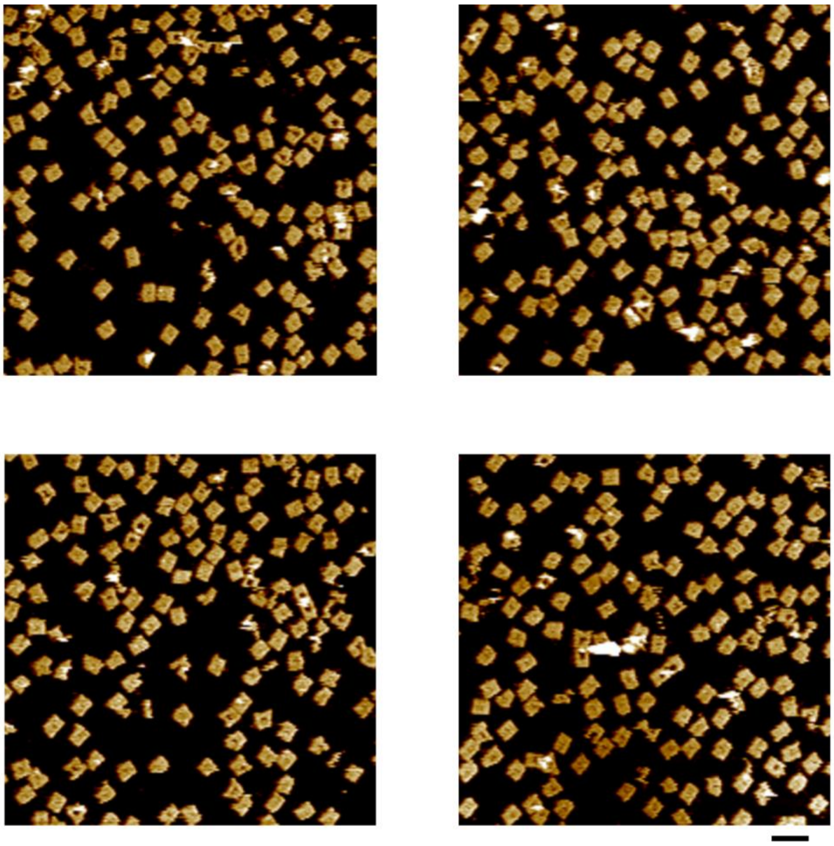

Figure S6. Four AFM images of the regenerated and locked origami tiles upon the treatment of the G-quadruplex unlocked tiles with anti-helper strands (H_{1a}'/H_{1b}' and H_{2a}'/H_{2b}') and CE (second cycle). Scale bar: 200 nm.

Table S5. Statistical analysis of the yields of the unlocked and locked origami tiles upon the treatment of the G-quadruplex unlocked tiles with anti-helper strands (H_{1a}'/H_{1b}' and H_{2a}'/H_{2b}') and CE (second cycle).

**Statistical analysis**		**Unlocked**	**Locked**	**Incomplete structure**
**1**	Count	14	99	14
	Yield (%)	11.0	78.0	
**2**	Count	7	114	11
	Yield (%)	5.3	86.4	
**3**	Count	10	110	10
	Yield (%)	7.7	84.6	
**4**	Count	10	110	14
	Yield (%)	7.5	82.1	

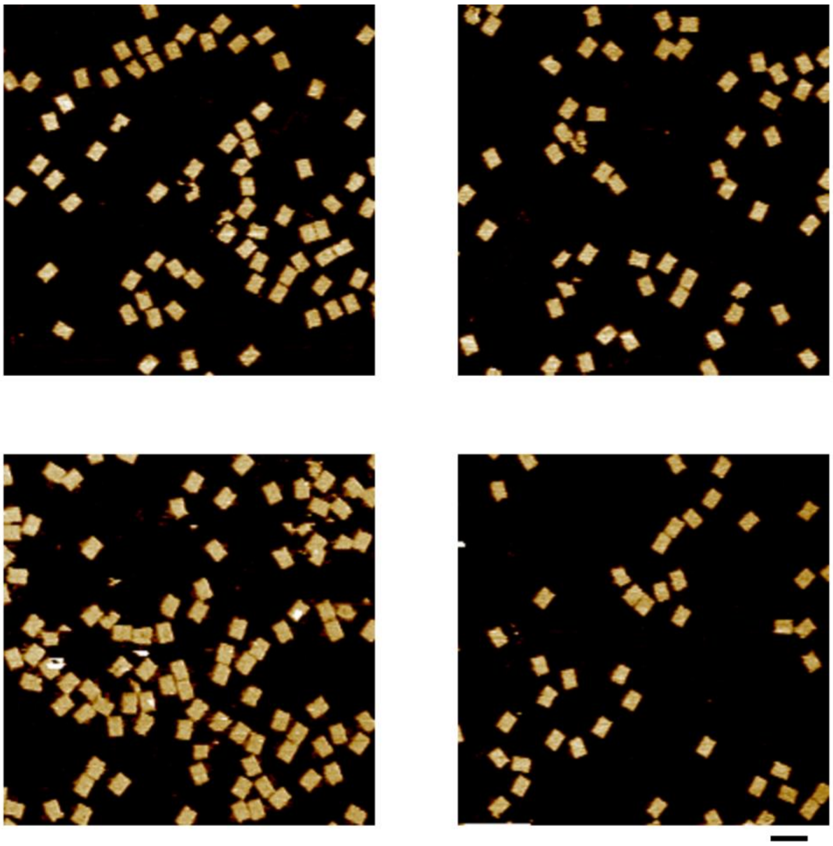

Figure S7. AFM images of the control experiment testing the unlocking of the G-quadruplex-responsive origami tiles in the presence of the helper hairpins H_1/H_2 and in the absence of K^+ ions. No tiles with nanoholes are observed, implying that the K^+ ions are essential to unlock the origami tiles to yield the nano-cavities. Scale bar: 200 nm.

Table S6. Statistical analysis of the yields of the unlocked and locked G-quadruplex-responsive origami tiles in the presence of the helper hairpins H_1/H_2 and in the absence of K^+ ions.

**Statistical analysis**		**Unlocked**	**Locked**	**Incomplete structure**
**1**	Count	0	69	3
	Yield (%)	0	95.8	
**2**	Count	0	48	4
	Yield (%)	0	92.3	
**3**	Count	0	41	0
	Yield (%)	0	100	
**4**	Count	0	85	4
	Yield (%)	0	95.5	

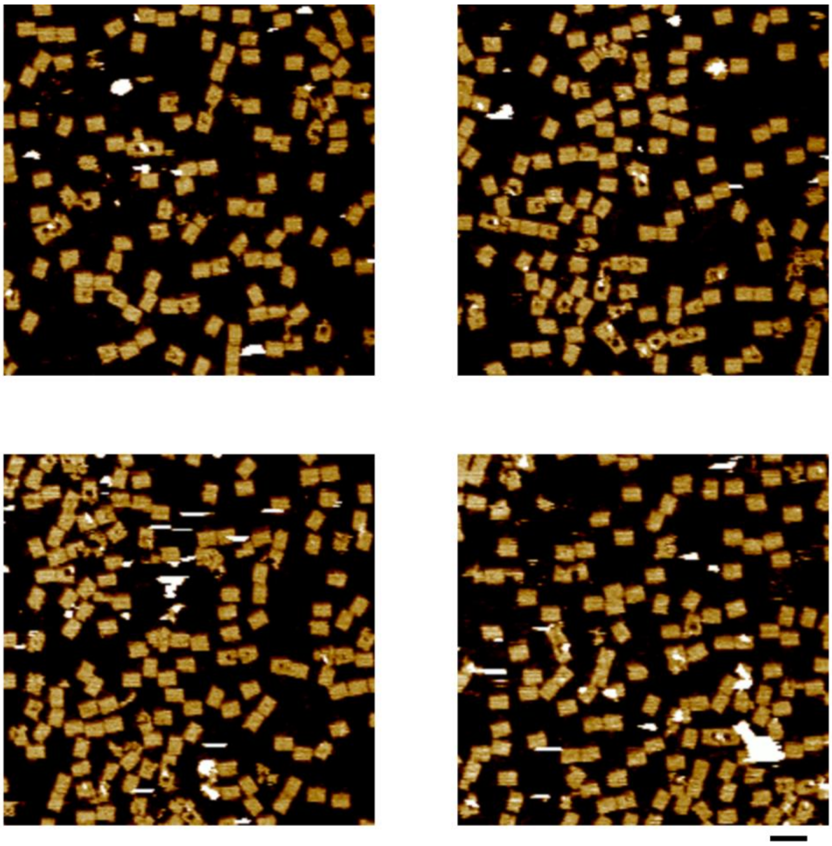

Figure S8. AFM images of the control experiment testing the K^+-ion-driven unlocking of the G-quadruplex-responsive origami tiles in the absence of the helper hairpins H_1/H_2. Only few origami tiles are with nanoholes. The results imply that the unlocked “window” exists in a flexible configuration that retains the nanohole closed without H_1/H_2 that stretch the “window” to a rigid open configuration by the hairpins/handles/anchor site units. Scale bar: 200 nm.

Table S7. Statistical analysis of the yields of the unlocked and locked origami tiles in the presence of K^+ ions and in the absence of the helper hairpins H_1/H_2.

**Statistical analysis**		**Unlocked**	**Locked**	**Incomplete structure**
**1**	Count	9	107	6
	Yield (%)	7.4	87.7	
**2**	Count	9	95	5
	Yield (%)	8.3	87.2	
**3**	Count	12	105	13
	Yield (%)	9.2	80.8	
**4**	Count	12	103	7
	Yield (%)	9.8	84.4	

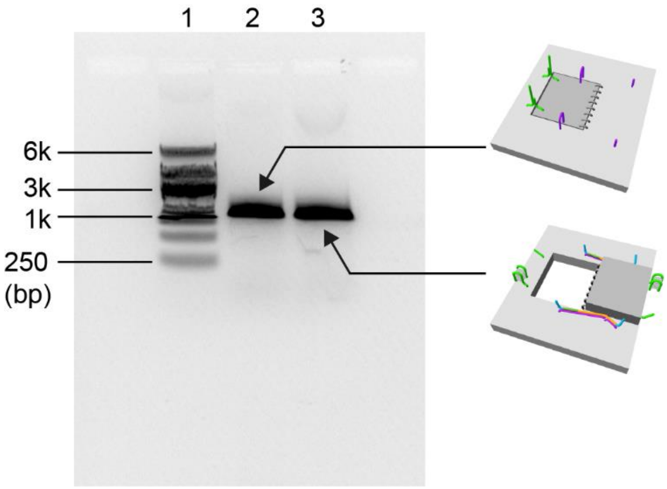

Figure S9. Electrophoretic Gel image of the locked and unlocked K^+-ion stabilized G-quadruplexes/crown ether-responsive origami tiles (lane 2 and 3, respectively). Lane 1 is 1kb reference ladder.

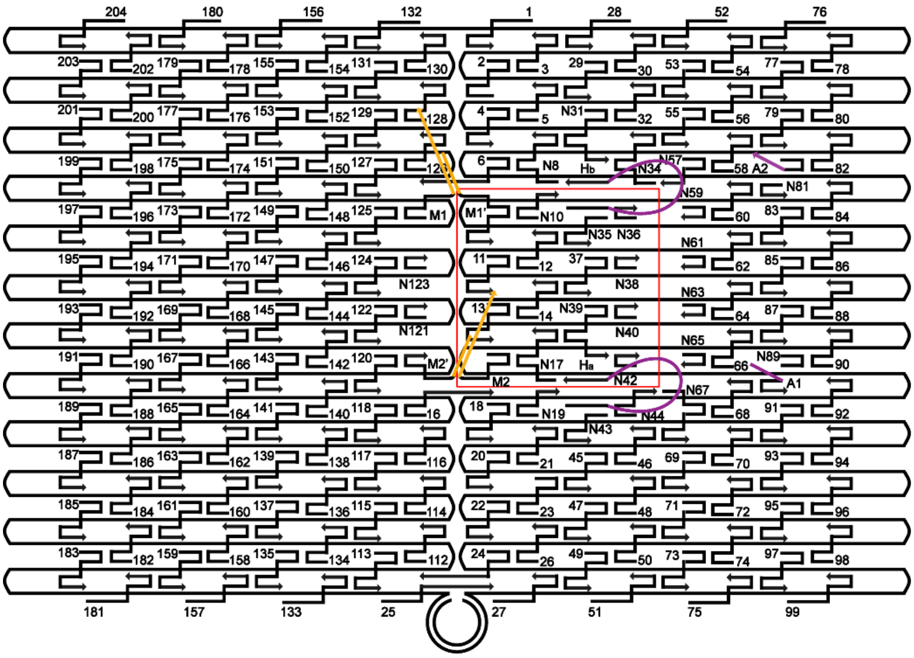

Figure S10. Schematic of the designed origami tile responsible to ATP molecules. The lock strands M1/M1’ and M2/M2’ shows in yellow, the handles (Ha, Hb) are purple loops and the anchoring tethers (A1, A2) are short purple lines. The red square shows the designed “nano-cavity” patch in the origami tile. The lock strands M1 and M2 contain the ATP aptamer sequences and can form the aptamer/ATP complex in the presence of ATP.

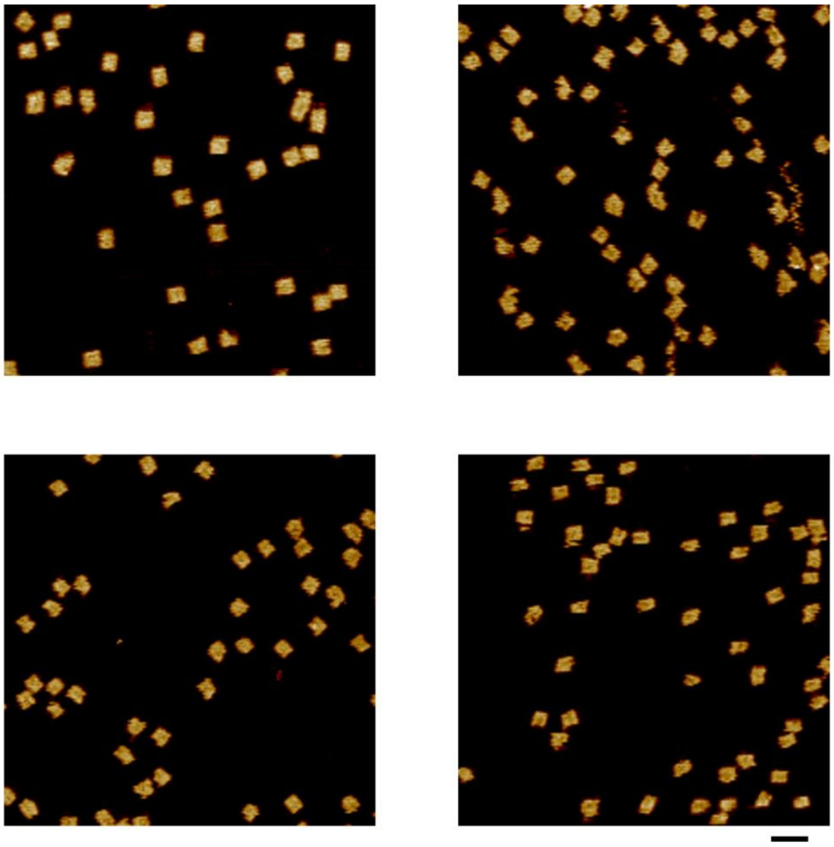

Figure S11. Four AFM images of the initial locked ATP-responsive origami tiles before the treatment of the tiles with ATP and the helper hairpins H_1/H_2. Scale bar: 200 nm.

Table S8. Statistical analysis of the yields of the unlocked and locked ATP-responsive origami tiles before the treatment of the tiles with ATP and the helper hairpins H_1/H_2.

**Statistical analysis**		**Unlocked**	**Locked**	**Incomplete structure**
**1**	Count	0	31	2
	Yield (%)	0	93.9	
**2**	Count	0	43	7
	Yield (%)	0	86.0	
**3**	Count	0	46	3
	Yield (%)	0	93.9	
**4**	Count	0	38	1
	Yield (%)	0	97.4	

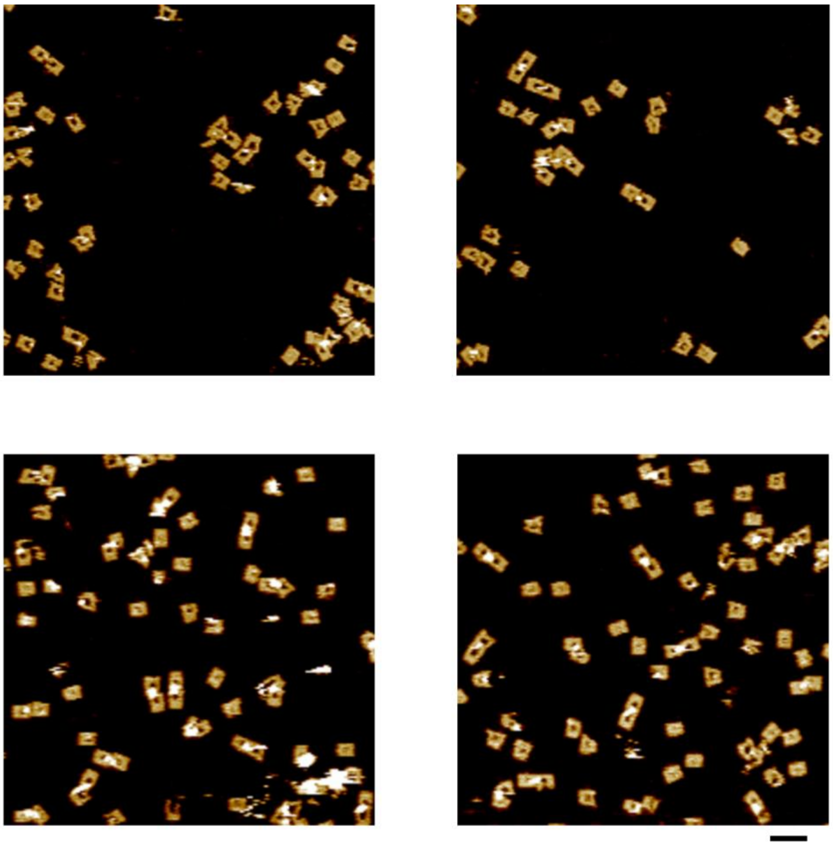

Figure S12. Four AFM images of the unlocked ATP-responsive origami tiles upon the treatment of the tiles with ATP and the helper hairpins H_1/H_2 (first cycle). Scale bar: 200 nm.

Table S9. Statistical analysis of the yields of the unlocked and locked ATP-responsive origami tiles in the unlocked state after the treatment with ATP and the helper hairpins H_1/H_2 (first cycle).

**Statistical analysis**		**Unlocked**	**Locked**	**Incomplete structure**
**1**	Count	32	7	4
	Yield (%)	74.4	16.3	
**2**	Count	23	8	2
	Yield (%)	69.7	24.2	
**3**	Count	40	14	5
	Yield (%)	67.8	23.7	
**4**	Count	47	14	4
	Yield (%)	72.3	21.5	

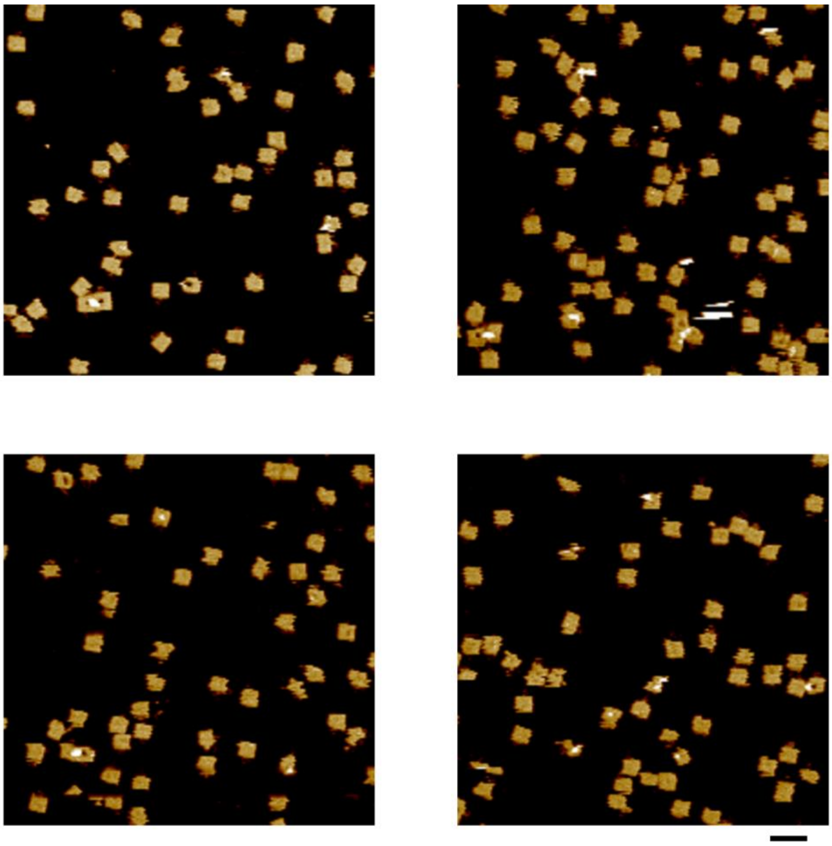

Figure S13. Four AFM images of the regenerated and locked ATP-responsive origami tiles upon the treatment of the unlocked cavity-containing tiles with the anti-helper strands (H_{1a}'/H_{1b}' and H_{2a}'/H_{2b}'), C-ATP$_a$ and C-ATP$_a$' (first cycle). Scale bar: 200 nm.

Table S10. Statistical analysis of the yields of the unlocked and locked ATP-responsive origami tiles upon the treatment of the unlocked tiles with anti-helper strands (H_{1a}'/H_{1b}' and H_{2a}'/H_{2b}'), C-ATP_a and C-ATP_a' (first cycle).

**Statistical analysis**		**Unlocked**	**Locked**	**Incomplete structure**
**1**	Count	3	42	2
	Yield (%)	6.4	89.4	
**2**	Count	3	54	5
	Yield (%)	4.8	87.1	
**3**	Count	1	43	8
	Yield (%)	1.9	82.7	
**4**	Count	2	40	6
	Yield (%)	4.2	83.3	

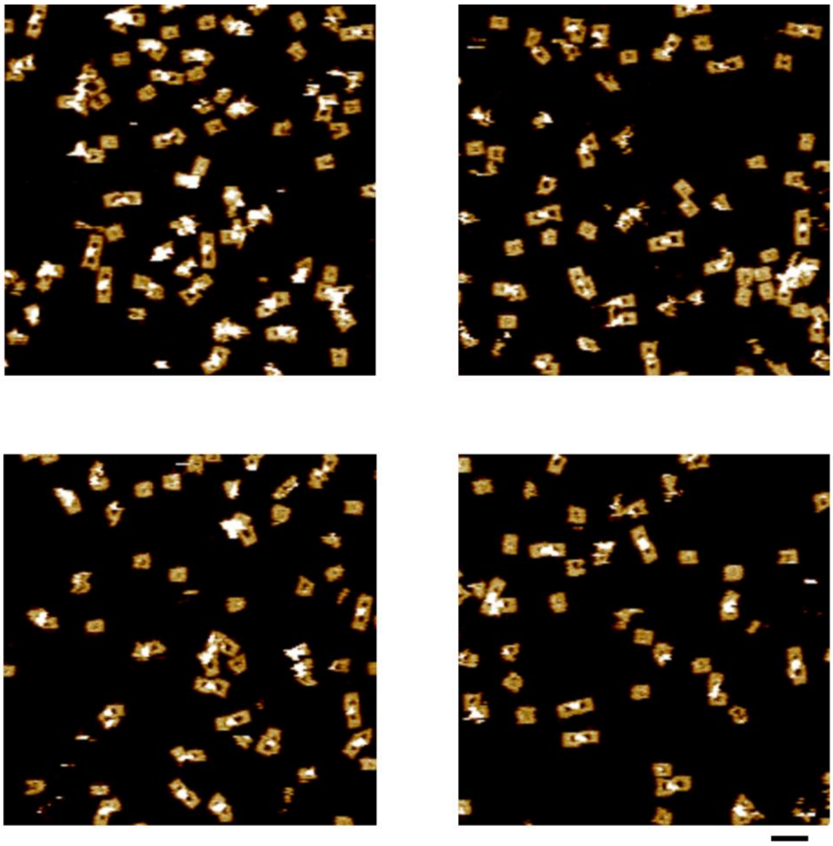

Figure S14. Four AFM images of the unlocked ATP-responsive origami tiles upon the treatment of the locked ATP-responsive tiles with ATP and the helper hairpins H_1/H_2 (second cycle). Scale bar: 200 nm.

Table S11. Statistical analysis of the yields of the unlocked and locked ATP-responsive origami tiles upon the treatment of the locked ATP-responsive tiles with ATP and the helper hairpins H_1/H_2 (second cycle).

**Statistical analysis**		**Unlocked**	**Locked**	**Incomplete structure**
**1**	Count	48	19	7
	Yield (%)	64.9	25.7	
**2**	Count	45	14	4
	Yield (%)	71.4	22.2	
**3**	Count	37	10	2
	Yield (%)	75.5	20.4	
**4**	Count	38	9	5
	Yield (%)	73.1	17.3	

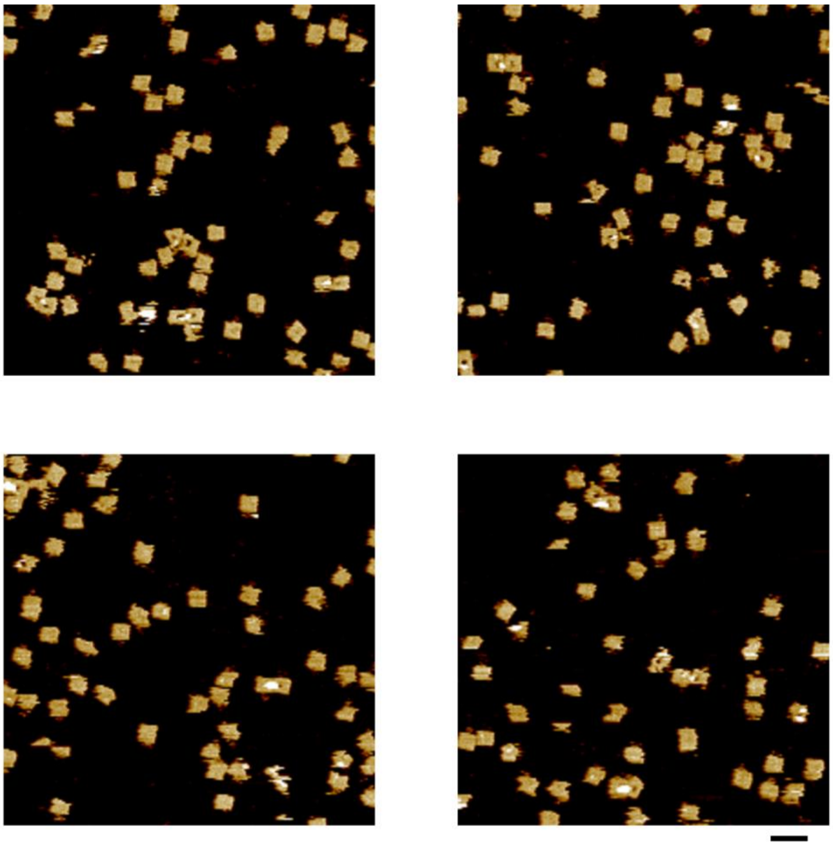

Figure S15. Four AFM images of the regenerated and locked ATP-responsive origami tiles upon the treatment of the unlocked cavity-containing ATP-responsive tiles with anti-helper strands (H_{1a}'/H_{1b}' and H_{2a}'/H_{2b}'), C-ATP_a and C-ATP_a' (second cycle). Scale bar: 200 nm.

Table S12. Statistical analysis of the yields of the unlocked and locked origami tiles upon the treatment of the unlocked cavity-containing ATP-responsive origami tiles with anti-helper strands (H_{1a}'/H_{1b}' and H_{2a}'/H_{2b}'), C-ATP_a and C-ATP_a' (second cycle).

**Statistical analysis**		**Unlocked**	**Locked**	**Incomplete structure**
**1**	Count	5	38	8
	Yield (%)	9.8	74.5	
**2**	Count	4	30	9
	Yield (%)	9.3	69.8	
**3**	Count	3	33	9
	Yield (%)	6.7	73.3	
**4**	Count	3	33	10
	Yield (%)	6.5	71.7	

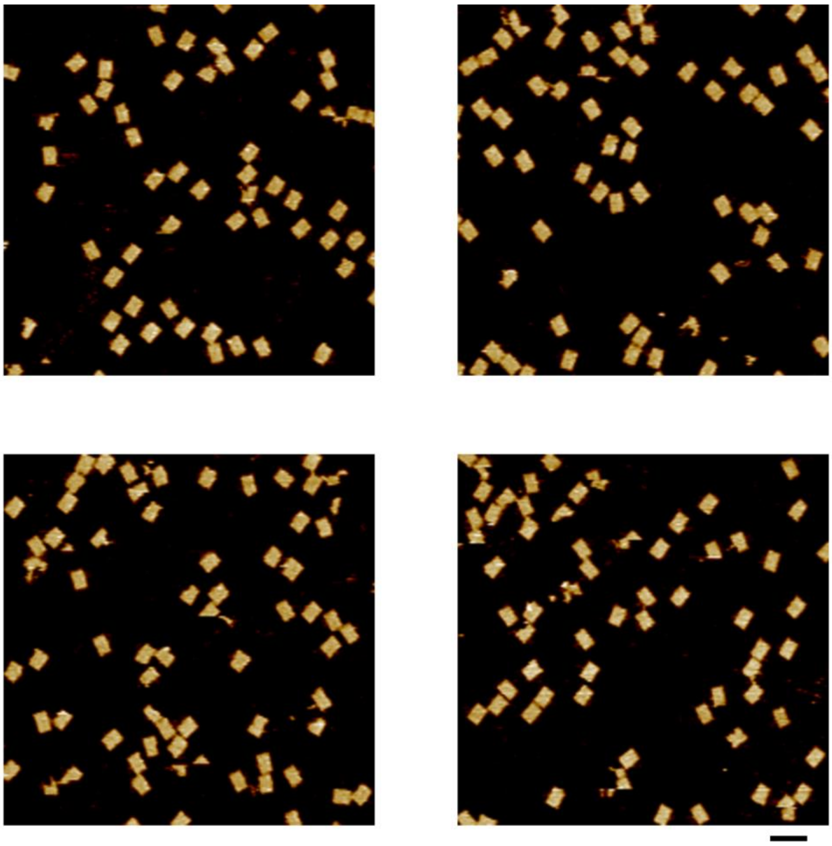

Figure S16. AFM images of the control experiment testing the unlocking of the ATP-responsive origami tiles in the presence of the helper hairpins H_1/H_2 and in the absence of ATP. No tiles with nanoholes are observed, implying that the ATP is essential to unlock the origami tiles to yield the nanoholes. Scale bar: 200 nm.

Table S13. Statistical analysis of the yields of the unlocked and locked ATP-responsive origami tiles in the presence of the helper hairpins H_1/H_2 and in the absence of ATP.

**Statistical analysis**		**Unlocked**	**Locked**	**Incomplete structure**
**1**	Count	0	48	3
	Yield (%)	0	94.1	
**2**	Count	0	54	4
	Yield (%)	0	93.1	
**3**	Count	0	54	6
	Yield (%)	0	90.0	
**4**	Count	0	52	6
	Yield (%)	0	89.7	

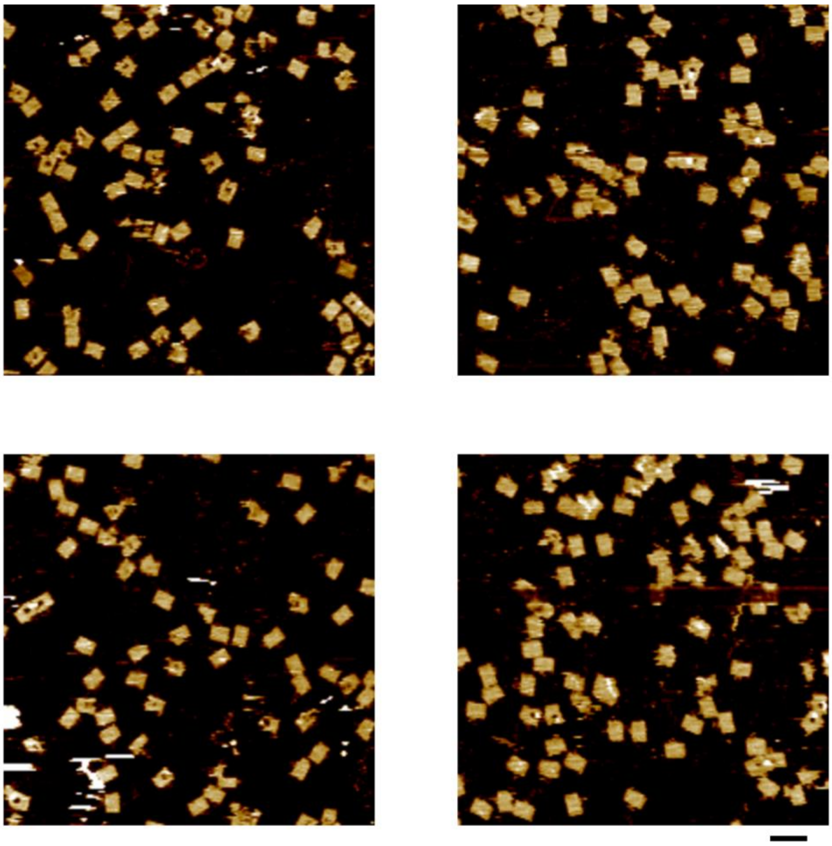

Figure S17. AFM images of the control experiment testing the ATP-driven unlocking of the origami tiles in the absence of the helper hairpins H_1/H_2. Only few origami tiles are with nanoholes. The results imply that the unlocked "window" exists in a flexible configuration that retains the nanohole closed without H_1/H_2 that stretch the "window" to a rigid configuration by the hairpins/handles/anchor site units. Scale bar: 200 nm.

Table S14. Statistical analysis of the yields of the unlocked and locked ATP-responsive origami tiles in the presence of ATP and in the absence of the helper hairpins H_1/H_2.

**Statistical analysis**		**Unlocked**	**Locked**	**Incomplete structure**
**1**	Count	6	52	7
	Yield (%)	9.2	80.0	
**2**	Count	6	60	3
	Yield (%)	8.7	87.0	
**3**	Count	7	55	7
	Yield (%)	10.1	79.7	
**4**	Count	5	56	5
	Yield (%)	7.6	84.8	

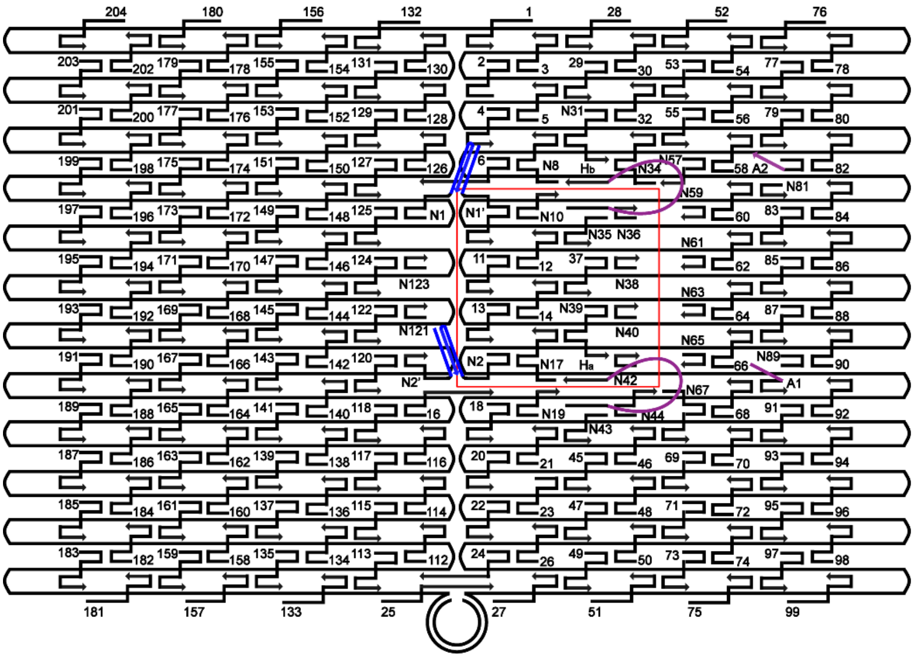

Figure S18. Schematic of the designed origami tile responsible to pH. The lock strands N1/N1’ and N2/N2’ shows in blue, the handles (Ha, Hb) are purple loops and the anchoring tethers (A1, A2) are short purple lines. The red square shows the designed “nano-cavity” patch in the origami tile. The lock strands can form the triplex structures at pH 6 and separate at pH 9.5.

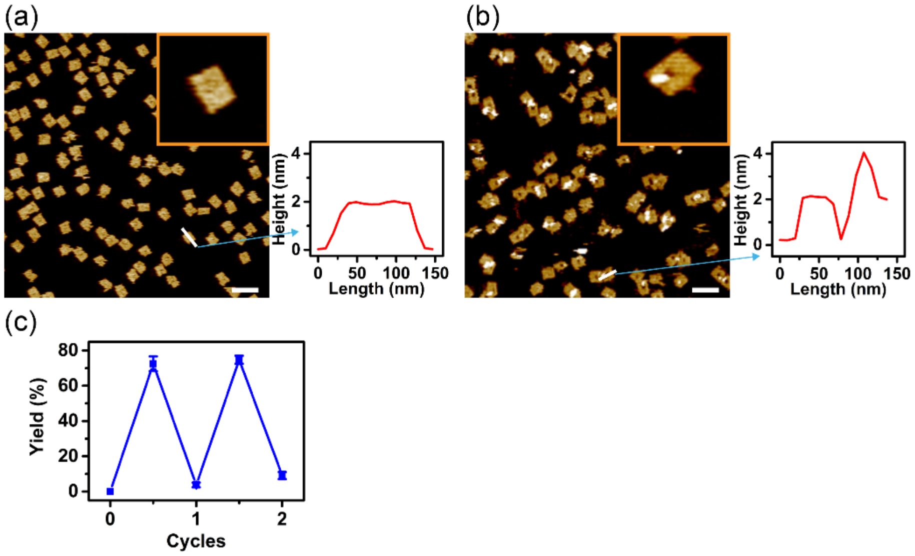

Figure S19. (a) AFM image of the T-A·T triplexes locked origami tiles and the corresponding cross-section analysis. Inset: Enlarged image of the T-A·T triplexes locked origami tile. Scale bar: 200 nm. (b) AFM image of the pH-driven unlocked nanocavity-containing origami tiles. Inset: Enlarged AFM image of the nanocavity containing origami raft. Scale bar: 200 nm. (c) Cyclic pH-triggered yields of the unlocked origami tiles generated at pH = 6.0 and pH = 9.5, respectively.

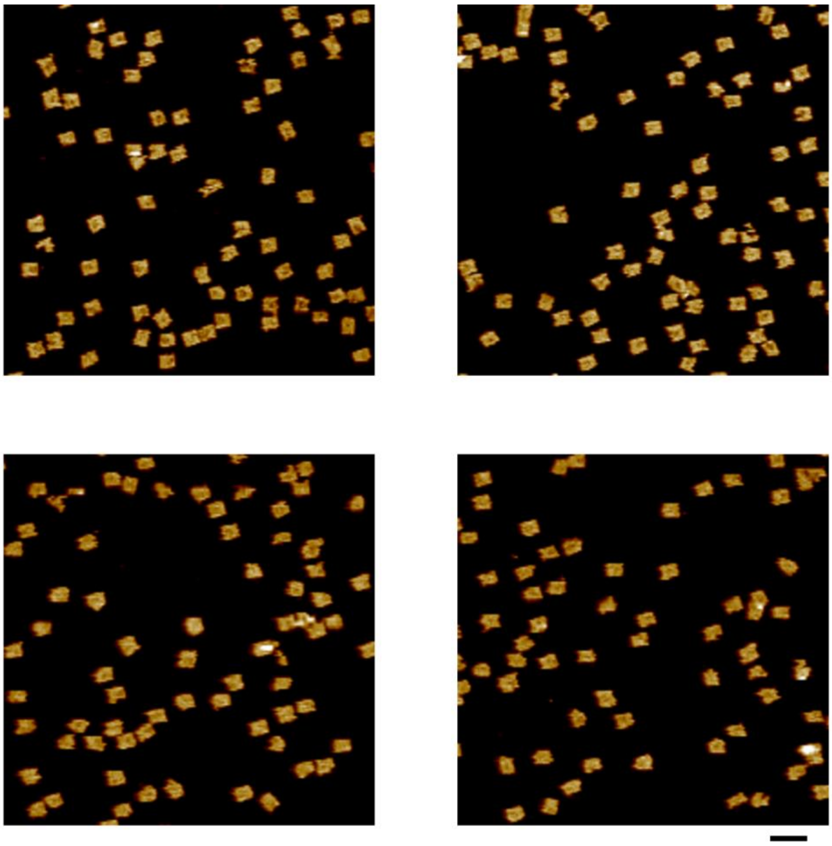

Figure S20. Four AFM images of the initial locked pH-responsive origami tiles before the treatment of the tiles at pH = 9.5 with the helper hairpins H_1/H_2. Scale bar: 200 nm.

Table S15. Statistical analysis of the yields of the unlocked and locked pH-responsive origami tiles before the treatment of the tiles at pH = 9.5 with the helper hairpins H_1/H_2.

**Statistical analysis**		**Unlocked**	**Locked**	**Incomplete structure**
**1**	Count	0	66	3
	Yield (%)	0	95.7	
**2**	Count	0	64	5
	Yield (%)	0	92.8	
**3**	Count	0	58	3
	Yield (%)	0	95.1	
**4**	Count	0	66	3
	Yield (%)	0	95.7	

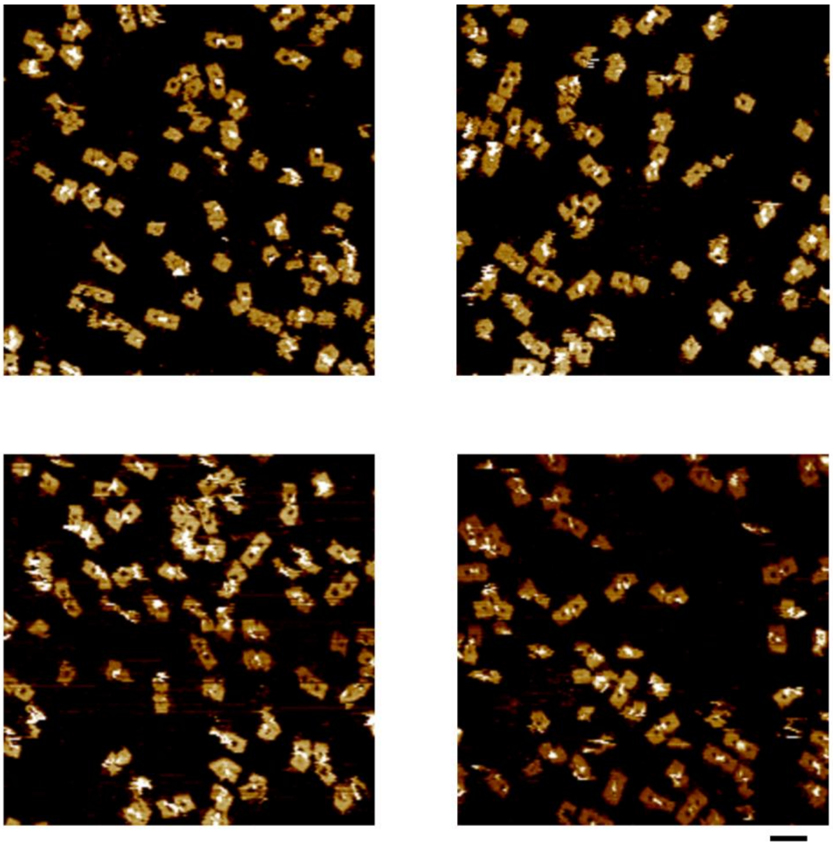

Figure S21. Four AFM images of the unlocked pH-responsive origami tiles upon the treatment of the tiles at pH = 9.5 with the helper hairpins H_1/H_2 (first cycle). Scale bar: 200 nm.

Table S16. Statistical analysis of the yields of the unlocked and locked pH-responsive origami tiles in the unlocked state after the treatment at pH = 9.5 with the helper hairpins H_1/H_2 (first cycle).

**Statistical analysis**		**Unlocked**	**Locked**	**Incomplete structure**
**1**	Count	40	11	9
	Yield (%)	66.7	18.3	
**2**	Count	49	10	6
	Yield (%)	75.4	15.4	
**3**	Count	53	12	5
	Yield (%)	75.7	17.1	
**4**	Count	52	13	7
	Yield (%)	72.2	18.1	

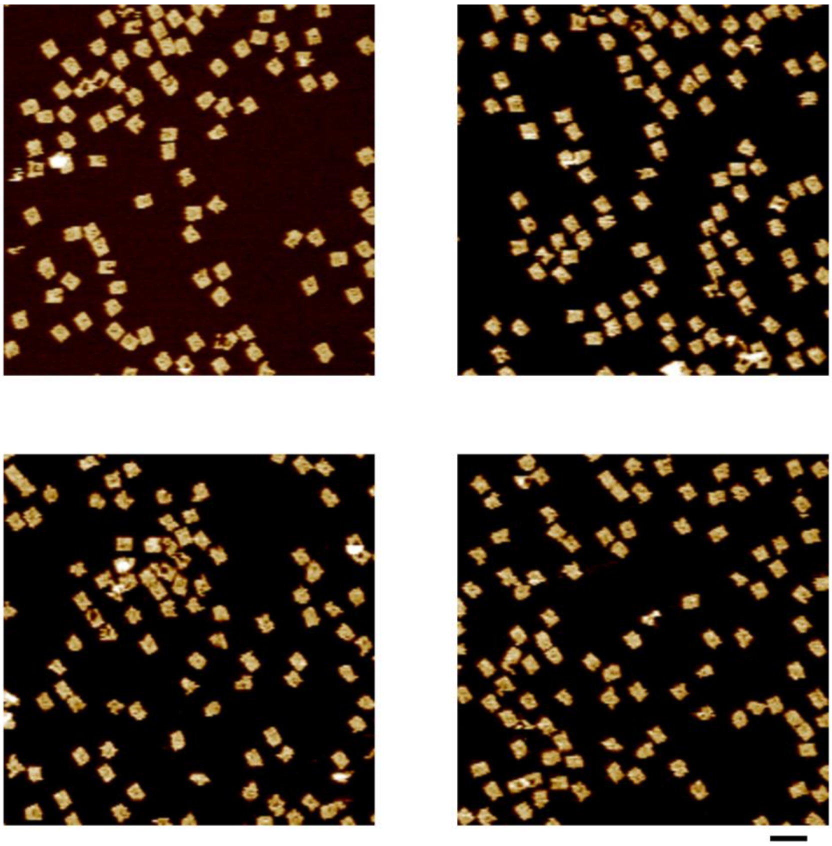

Figure S22. Four AFM images of the regenerated and locked origami tiles upon the treatment of the unlocked pH-responsive tiles with anti-helper strands (H_{1a}'/H_{1b}' and H_{2a}'/H_{2b}') at pH = 6 (first cycle). Scale bar: 200 nm.

Table S17. Statistical analysis of the yields of the unlocked and locked origami tiles upon the treatment of the unlocked pH-responsive tiles with anti-helper strands (H_{1a}'/H_{1b}' and H_{2a}'/H_{2b}') at pH = 6 (first cycle).

**Statistical analysis**		**Unlocked**	**Locked**	**Incomplete structure**
**1**	Count	3	82	5
	Yield (%)	3.3	91.1	
**2**	Count	5	91	7
	Yield (%)	4.9	88.3	
**3**	Count	2	92	7
	Yield (%)	2.0	91.1	
**4**	Count	4	76	7
	Yield (%)	4.6	87.4	

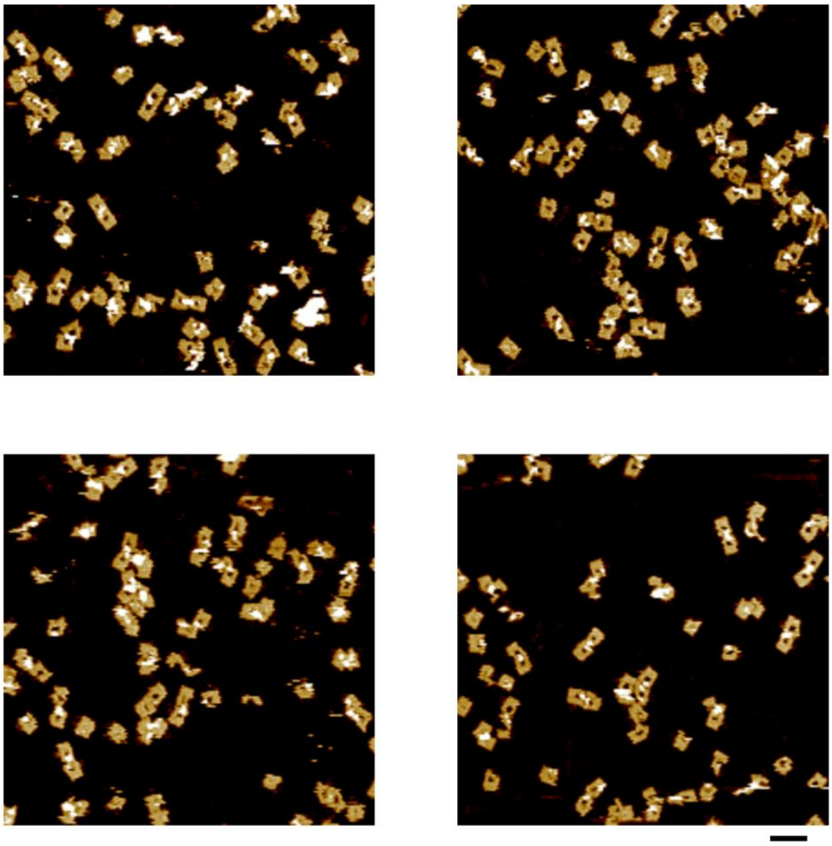

Figure S23. Four AFM images of the unlocked origami tiles upon the treatment of the pH-responsive tiles at pH = 9.5 with the helper hairpins H_1/H_2 (second cycle). Scale bar: 200 nm.

Table S18. Statistical analysis of the yields of the unlocked and locked origami tiles upon the treatment of the pH-responsive tiles at pH = 9.5 with the helper hairpins H_1/H_2 (second cycle).

**Statistical analysis**		**Unlocked**	**Locked**	**Incomplete structure**
**1**	Count	40	8	4
	Yield (%)	76.9	15.4	
**2**	Count	44	10	4
	Yield (%)	75.9	17.2	
**3**	Count	38	9	6
	Yield (%)	71.7	17.0	
**4**	Count	38	10	3
	Yield (%)	74.5	19.6	

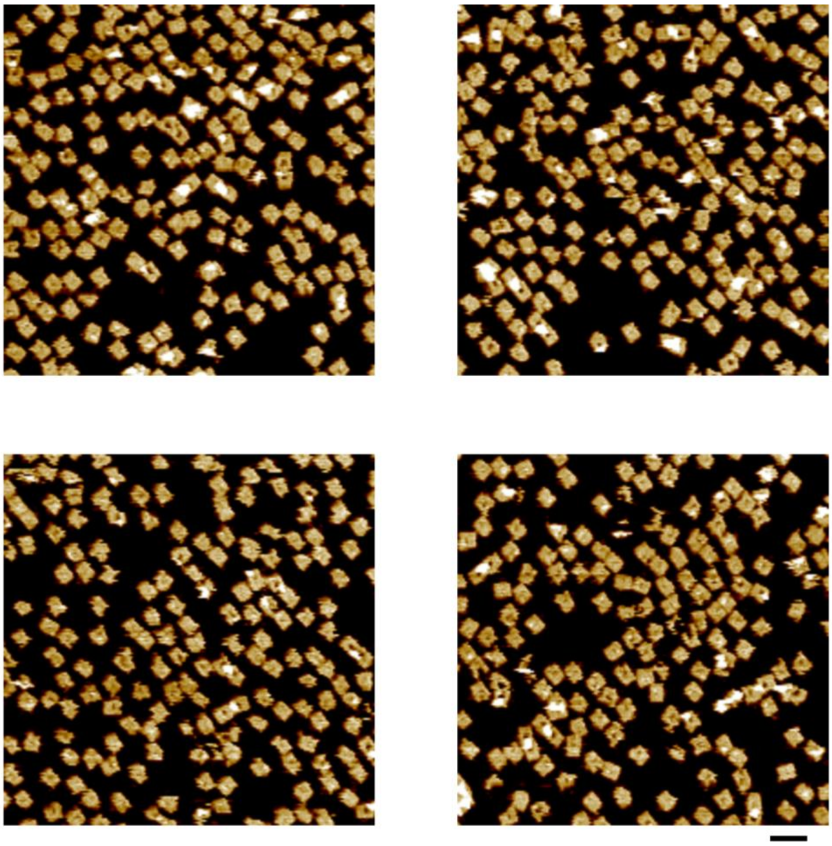

Figure S24. Four AFM images of the regenerated and locked pH-responsive origami tiles, upon the treatment of the unlocked tiles with anti-helper strands (H_{1a}'/H_{1b}' and H_{2a}'/H_{2b}') at pH = 6 (second cycle). Scale bar: 200 nm.

Table S19. Statistical analysis of the yields of the unlocked and locked origami tiles upon the treatment of the unlocked pH-responsive tiles with anti-helper strands (H_{1a}'/H_{1b}' and H_{2a}'/H_{2b}') at pH = 6 (second cycle).

**Statistical analysis**		**Unlocked**	**Locked**	**Incomplete structure**
**1**	Count	12	136	21
	Yield (%)	7.1	80.5	
**2**	Count	19	120	20
	Yield (%)	11.9	75.5	
**3**	Count	14	119	21
	Yield (%)	9.1	77.3	
**4**	Count	13	125	20
	Yield (%)	8.2	79.1	

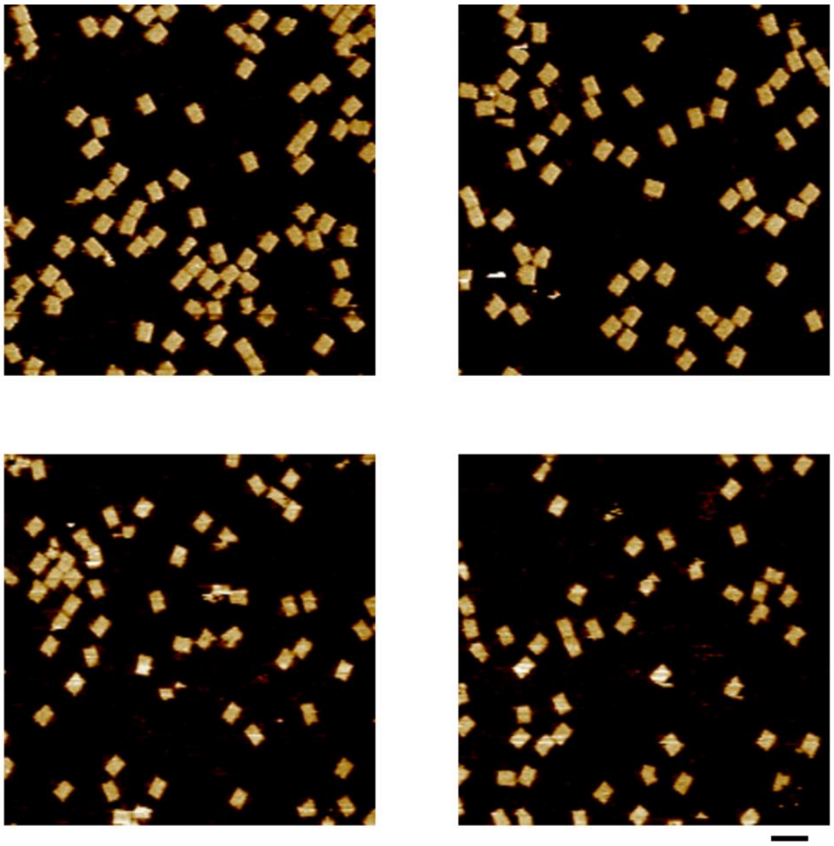

Figure S25. AFM images of the control experiment testing the unlocking of the pH-responsive origami tiles in the presence of the helper hairpins H_1/H_2 at pH = 6. No tiles with nanoholes are observed, implying that the pH = 9.5 is essential to unlock the origami tiles to yield the nanoholes. Scale bar: 200 nm.

Table S20. Statistical analysis of the yields of the unlocked and locked pH-responsive origami tiles in the presence of the helper hairpins H_1/H_2 at pH = 6.

**Statistical analysis**		**Unlocked**	**Locked**	**Incomplete structure**
**1**	Count	0	46	2
	Yield (%)	0	95.8	
**2**	Count	0	50	5
	Yield (%)	0	90.9	
**3**	Count	0	77	5
	Yield (%)	0	93.9	
**4**	Count	0	62	1
	Yield (%)	0	98.4	

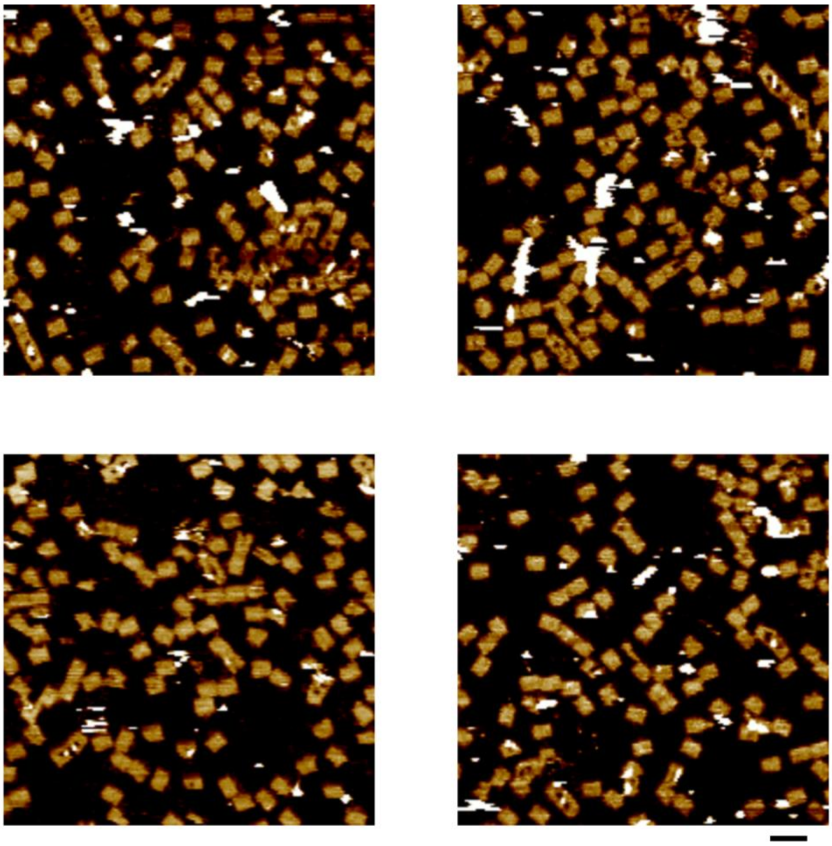

Figure S26. AFM images of the control experiment testing the pH-driven unlocking of the pH-responsive origami tiles in the absence of the helper hairpins H_1/H_2. Only few origami tiles are with nanoholes. The results imply that the unlocked “window” exists in a flexible configuration that retains the nanohole closed without H_1/H_2 that stretch the “window” to a rigid configuration by the hairpins/handles/anchor site units. Scale bar: 200 nm.

Table S21. Statistical analysis of the yields of the unlocked and locked pH-responsive origami tiles at pH = 9.5 and in the absence of the helper hairpins H_1/H_2.

**Statistical analysis**		**Unlocked**	**Locked**	**Incomplete structure**
**1**	Count	11	98	4
	Yield (%)	9.7	86.7	
**2**	Count	11	99	10
	Yield (%)	9.2	82.5	
**3**	Count	10	93	8
	Yield (%)	9.0	83.8	
**4**	Count	9	92	6
	Yield (%)	8.4	86.0	

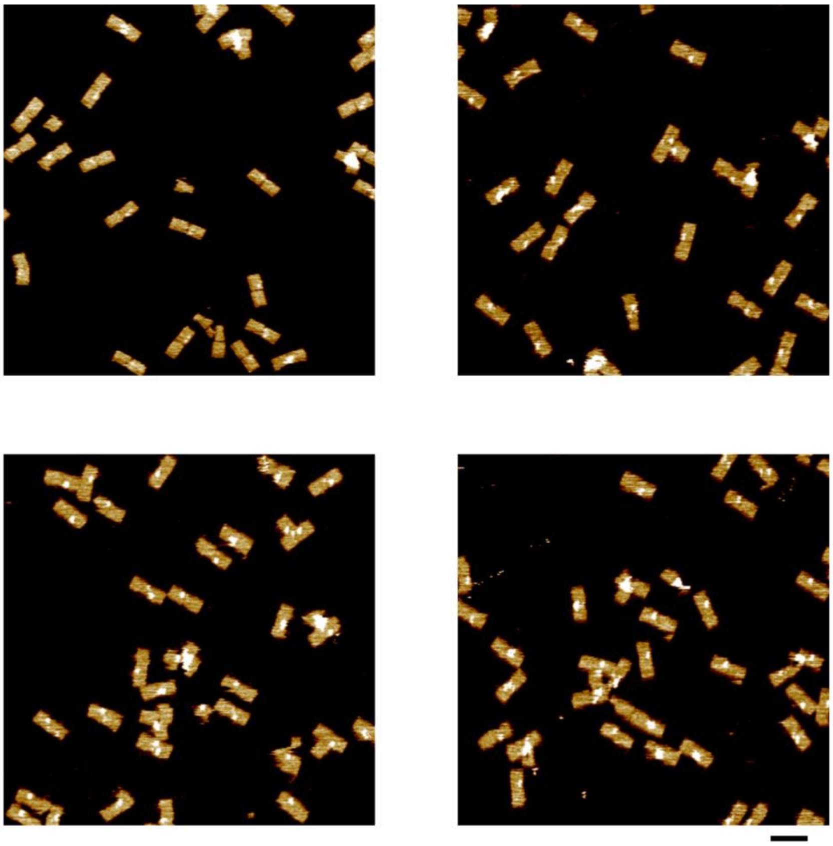

Figure S27. Four AFM images of the initial locked ATP-/K^+-ion-responsive origami dimers (state I) before the treatment with K^+ ions or ATP and the helper hairpins H_1/H_2. Scale bar: 200 nm.

Table S22. Statistical analysis of the yields of the unlocked and locked ATP-/K^+-ion-responsive origami dimers (state I) before the treatment with K^+ ions or ATP and the helper hairpins H_1/H_2.

**Statistical analysis**		**Dimer**				**Monomer**				**Incomplete structures**
		**O/O**	**O/C**	**C/O**	**C/C**	**L-O**	**L-C**	**R-O**	**R-C**	
**1**	Count	0	0	0	20	0	2	0	2	4
	Yield (%)	0	0	0	83.3					
**2**	Count	0	0	0	19	0	2	0	2	4
	Yield (%)	0	0	0	82.6					
**3**	Count	0	0	0	23	0	3	0	3	4
	Yield (%)	0	0	0	82.1					
**4**	Count	0	0	0	25	0	2	0	2	4
	Yield (%)	0	0	0	86.2					

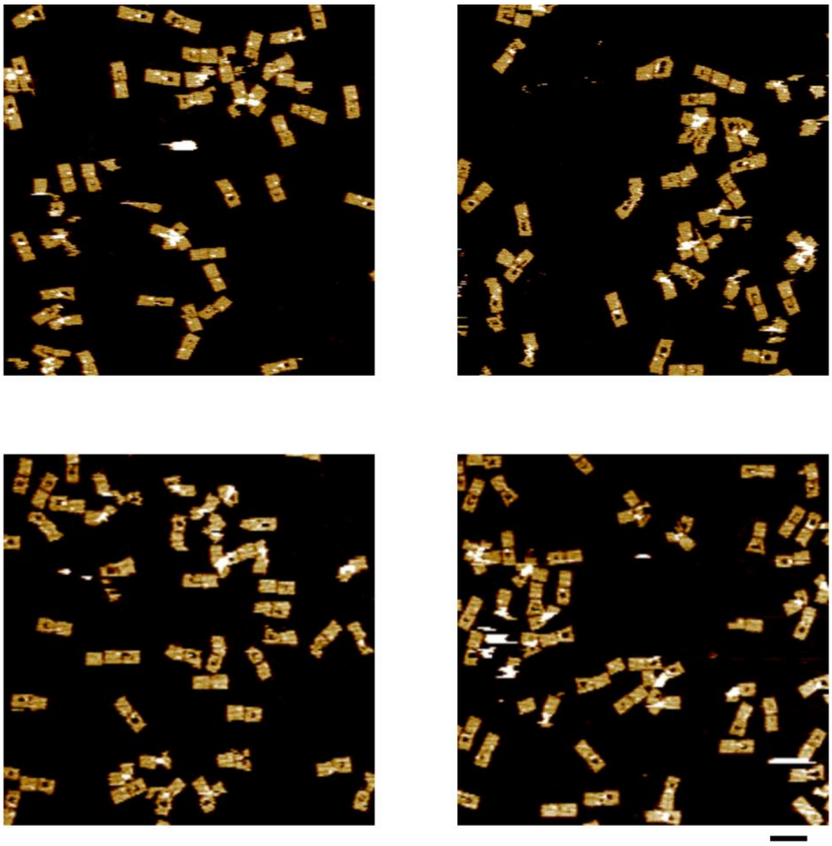

Figure S28. Four AFM images of the ATP-/K^+-ion-responsive origami dimers with unlocked nanocavity on the unmarked tile (state II) upon the treatment with ATP and the helper hairpins H_1/H_2. Scale bar: 200 nm.

Table S23. Statistical analysis of the yields of the unlocked and locked ATP-/K^+-ion-responsive origami dimers (state II) upon the treatment with ATP and the helper hairpins H_1/H_2.

**Statistical analysis**		**Dimer**				**Monomer**				**Incomplete structures**
		**O/O**	**O/C**	**C/O**	**C/C**	**L-O**	**L-C**	**R-O**	**R-C**	
**1**	Count	0	22	0	10	1	1	0	3	5
	Yield (%)	0	59.5	0	27.0					
**2**	Count	0	19	0	2	1	1	0	2	12
	Yield (%)	0	65.5	0	6.9					
**3**	Count	0	28	0	6	2	4	0	6	8
	Yield (%)	0	63.6	0	13.6					
**4**	Count	0	26	0	6	1	0	0	1	10
	Yield (%)	0	68.4	0	15.8					

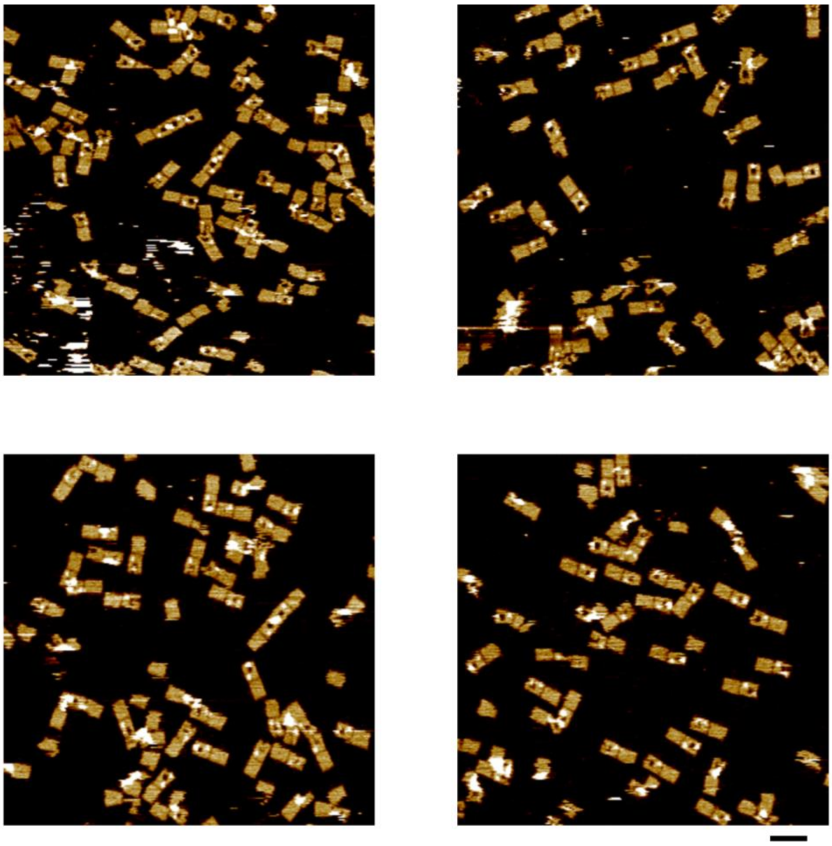

Figure S29. Four AFM images of the ATP-/K^+-ion-responsive origami dimers with unlocked nanocavity on the marked tile (state III) upon the treatment with K^+ ions and the helper hairpins H_1/H_2. Scale bar: 200 nm.

Table S24. Statistical analysis of the yields of the unlocked and locked ATP-/K^+-ion-responsive origami dimers (state III) upon the treatment with K^+ ions and the helper hairpins H_1/H_2.

**Statistical analysis**		**Dimer**				**Monomer**				**Incomplete structures**
		**O/O**	**O/C**	**C/O**	**C/C**	**L-O**	**L-C**	**R-O**	**R-C**	
**1**	Count	0	0	35	8	1	5	4	2	2
	Yield (%)	0	0	70.0	16.0					
**2**	Count	0	0	20	6	1	3	3	0	9
	Yield (%)	0	0	58.8	17.6					
**3**	Count	0	0	25	3	1	3	2	2	8
	Yield (%)	0	0	69.4	8.3					
**4**	Count	0	1	28	5	0	6	2	3	5
	Yield (%)	0	2.4	66.7	11.9					

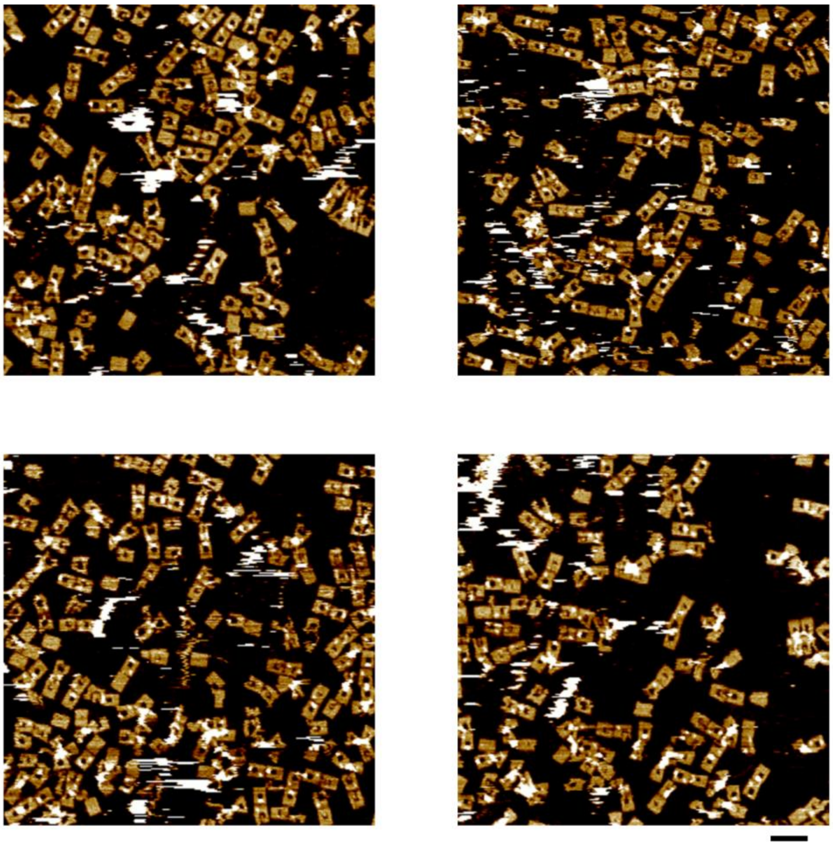

Figure S30. Four AFM images of the ATP-/K^+-ion-responsive origami dimers with unlocked nanocavities on both tiles (state IV) upon the treatment with K^+ ions/ATP and the helper hairpins H_1/H_2. Scale bar: 200 nm.

Table S25. Statistical analysis of the yields of the unlocked and locked ATP-/K^+-ion-responsive origami dimers (state IV) upon the treatment with K^+ ions/ATP and the helper hairpins H_1/H_2.

**Statistical analysis**		**Dimer**				**Monomer**				**Incomplete structures**
		**O/O**	**O/C**	**C/O**	**C/C**	**L-O**	**L-C**	**R-O**	**R-C**	
**1**	Count	35	3	3	3	12	9	7	2	30
	Yield (%)	47.3	4.1	4.1	4.1					
**2**	Count	29	4	5	2	8	5	4	3	20
	Yield (%)	48.3	6.7	8.3	3.3					
**3**	Count	42	5	7	2	8	6	5	4	33
	Yield (%)	50.0	6.0	8.3	2.4					
**4**	Count	37	4	4	4	13	7	4	2	28
	Yield (%)	48.7	5.3	5.3	5.3					

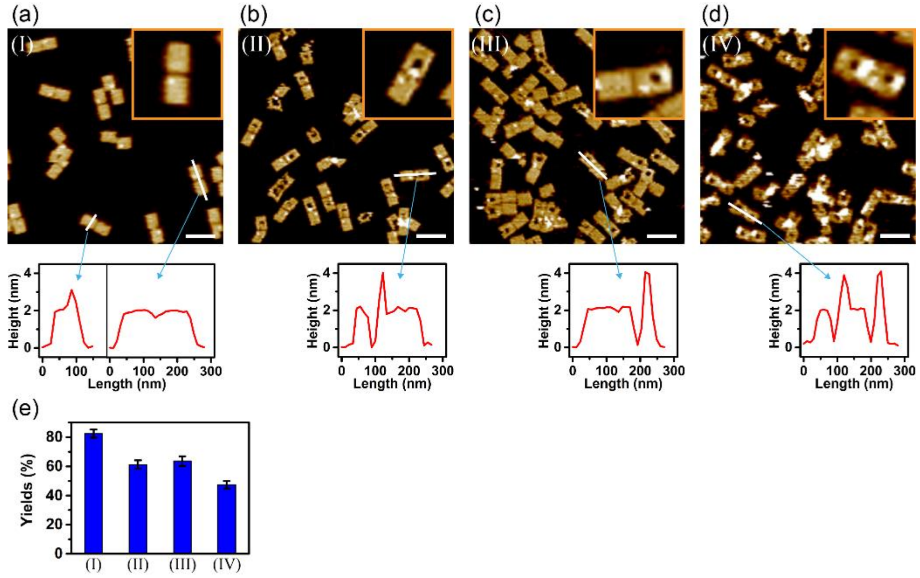

Figure S31. The AFM images and respective cross-section analyses of the dimers are presented in: (a) The locked dimer, configuration I, (b) Configuration II, (c) Configuration III, (d) Configuration IV. (a)-(d), Scale bars: 200 nm. (e) Yields of origami structures corresponding to the closed dimer I and to the nanocavity-containing origami structures II, III and IV generated by the respective triggers.

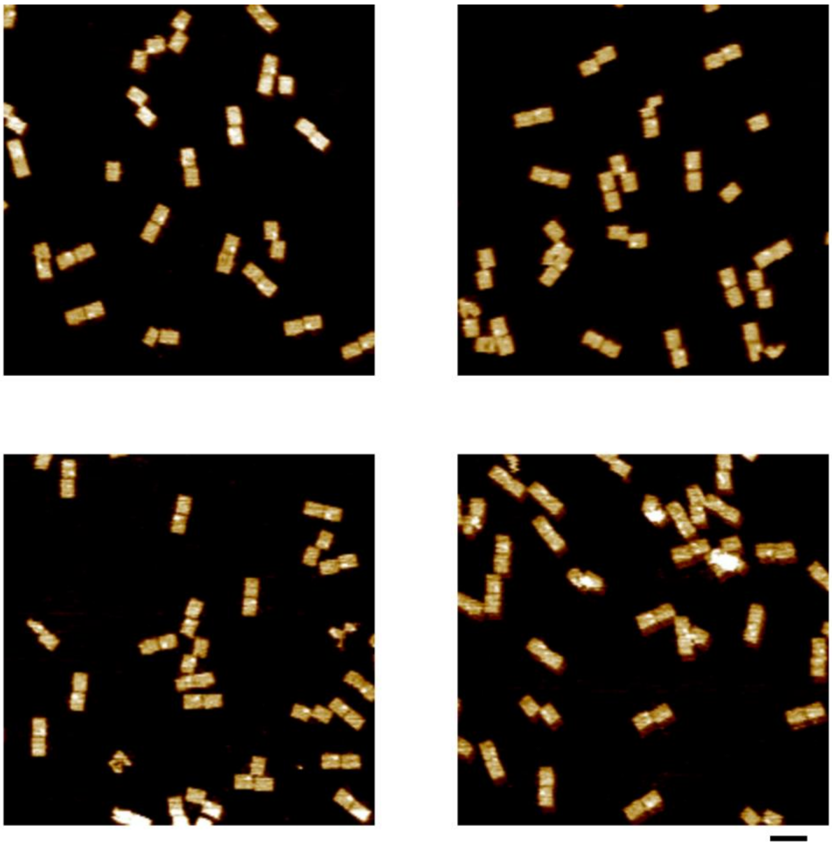

Figure S32. Four AFM images of the pH-/K^+-ion-responsive origami dimers (state I) before the treatment with K^+ ions or pH 9.5 buffer and the helper hairpins H_1/H_2. Scale bar: 200 nm.

Table S26. Statistical analysis of the yields of the unlocked and locked pH-/K^+-ion-responsive origami dimers (state I) before the treatment with K^+ ions or pH 9.5 buffer and the helper hairpins H_1/H_2.

**Statistical analysis**		**Dimer**				**Monomer**				**Incomplete structures**
		**O/O**	**O/C**	**C/O**	**C/C**	**L-O**	**L-C**	**R-O**	**R-C**	
**1**	Count	0	0	0	18	0	3	0	3	2
	Yield (%)	0	0	0	81.8					
**2**	Count	0	0	0	19	0	3	0	2	1
	Yield (%)	0	0	0	86.4					
**3**	Count	0	0	0	16	0	4	0	4	0
	Yield (%)	0	0	0	80.0					
**4**	Count	0	0	0	22	0	4	0	5	1
	Yield (%)	0	0	0	81.5					

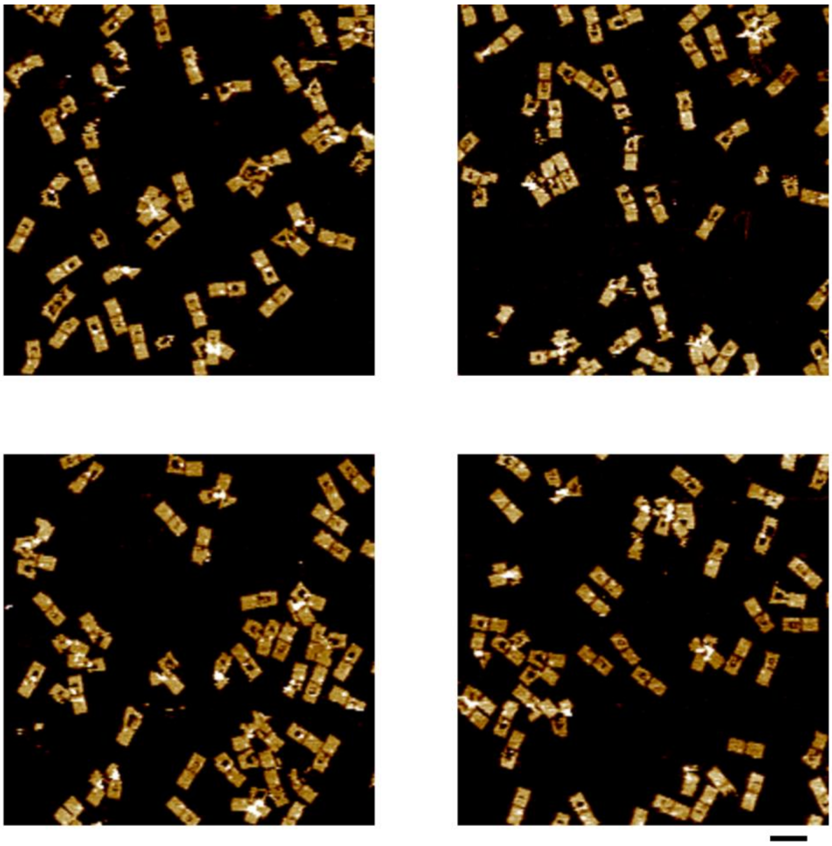

Figure S33. Four AFM images of the pH-/K^+-ion-responsive origami dimers with unlocked nanocavity on the unmarked tile (state II) upon the treatment of the dimers at pH 9.5 buffer and the helper hairpins H_1/H_2. Scale bar: 200 nm.

Table S27. Statistical analysis of the yields of the unlocked and locked pH-/K^+-ion-responsive origami dimers (state II) upon the treatment in pH 9.5 buffer and the helper hairpins H_1/H_2.

**Statistical analysis**		**Dimer**				**Monomer**				**Incomplete structures**
		**O/O**	**O/C**	**C/O**	**C/C**	**L-O**	**L-C**	**R-O**	**R-C**	
**1**	Count	0	25	0	5	5	4	0	5	6
	Yield (%)	0	62.5	0	12.5					
**2**	Count	0	22	0	5	5	4	0	5	6
	Yield (%)	0	59.5	0	13.5					
**3**	Count	0	21	0	4	4	4	0	7	7
	Yield (%)	0	58.3	0	11.1					
**4**	Count	0	22	0	5	4	3	0	4	3
	Yield (%)	0	64.7	0	14.7					

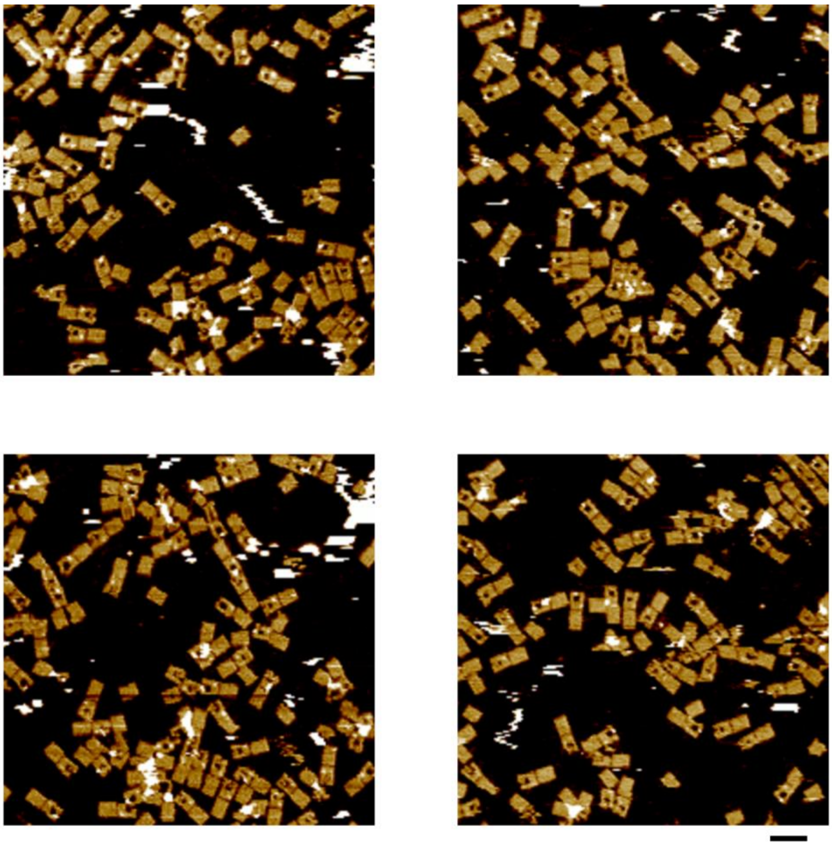

Figure S34. Four AFM images of the pH-/K^+-ion-responsive origami dimers with unlocked nanocavity on the marked tile (state III) upon the treatment with K^+ ions and the helper hairpins H_1/H_2. Scale bar: 200 nm.

Table S28. Statistical analysis of the yields of the unlocked and locked pH-/K^+-ion-responsive origami dimers (state III) upon the treatment with K^+ ions and the helper hairpins H_1/H_2.

**Statistical analysis**		**Dimer**				**Monomer**				**Incomplete structures**
		**O/O**	**O/C**	**C/O**	**C/C**	**L-O**	**L-C**	**R-O**	**R-C**	
**1**	Count	0	1	31	12	0	8	1	5	4
	Yield (%)	0	1.9	58.5	22.6					
**2**	Count	0	0	36	9	1	9	3	4	5
	Yield (%)	0	0	64.3	16.1					
**3**	Count	0	1	38	5	0	12	9	6	11
	Yield (%)	0	1.6	60.3	7.9					
**4**	Count	0	0	36	4	1	9	5	4	7
	Yield (%)	0	0	67.9	7.5					

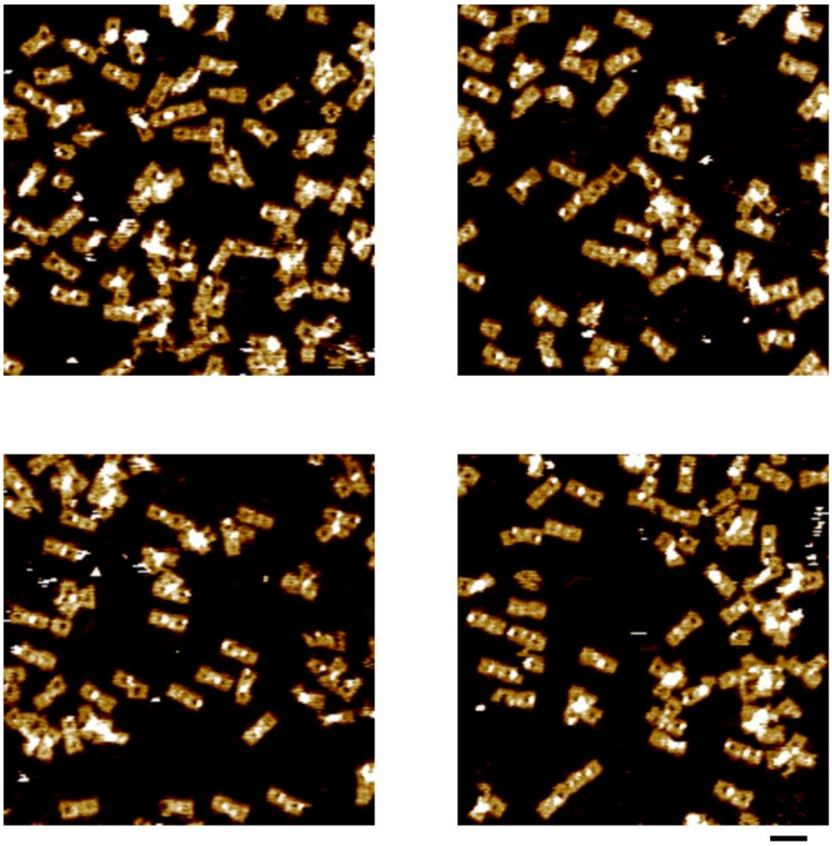

Figure S35. Four AFM images of the pH-/K^+-ion-responsive origami dimers with unlocked nanocavities on both tiles (state IV) upon the treatment with K^+ ions and the helper hairpins H_1/H_2 at pH 9.5. Scale bar: 200 nm.

Table S29. Statistical analysis of the yields of the unlocked and locked pH-/K^+-ion-responsive origami dimers (state IV) upon the treatment with K^+ ions and the helper hairpins H_1/H_2 at pH 9.5.

**Statistical analysis**		**Dimer**				**Monomer**				**Incomplete structures**
		**O/O**	**O/C**	**C/O**	**C/C**	**L-O**	**L-C**	**R-O**	**R-C**	
**1**	Count	14	4	3	2	2	2	3	1	8
	Yield (%)	45.2	12.9	9.7	6.5					
**2**	Count	23	3	3	6	5	3	5	3	6
	Yield (%)	50.0	6.5	6.5	13.0					
**3**	Count	17	3	4	3	3	3	3	3	4
	Yield (%)	48.6	8.6	11.4	8.6					
**4**	Count	16	3	2	3	4	4	3	2	7
	Yield (%)	47.1	8.8	5.9	8.8					

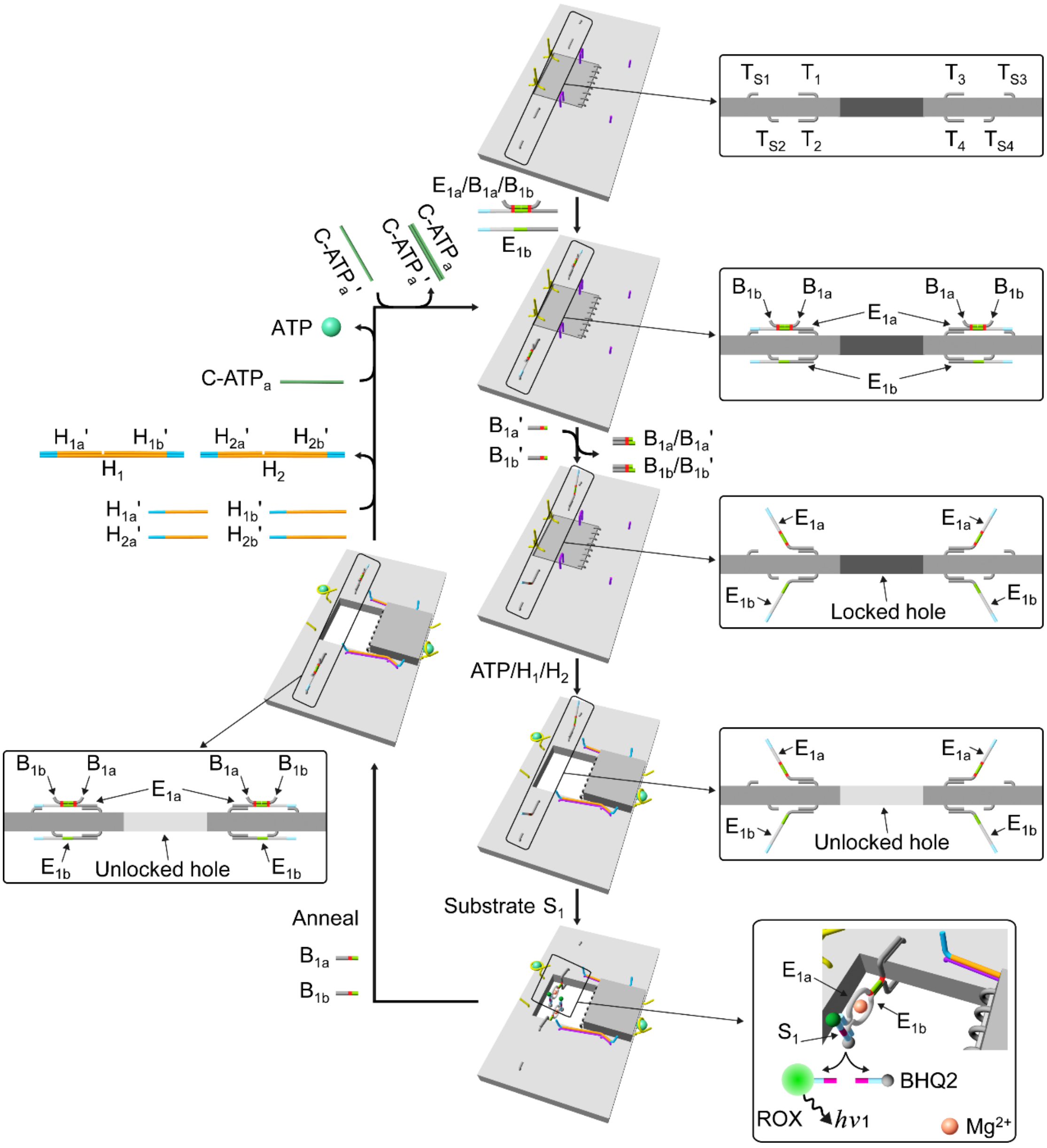

Figure S36. Schematic of engineering of the origami tile (only showing the core part of the tile) and the switchable catalysis of the Mg^{2+}-ion-dependent DNAzyme in the nanocavity. The tile includes the ATP-driven unlocking apparatus (discussed in Fig. 3 and text). Protruding tethers T_1/T_3 and T_2/T_4 are designed on the opposite sides of the origami tile. The duplex $E_{1a}/B_{1a}/B_{1b}$ and the strand E_{1b} are hybridized with the tethers T_1/T_3 and T_2/T_4, respectively. Prior to the unlocking of the tile, $E_{1a}/B_{1a}/B_{1b}$ are unblocked by the strand displacement process, using appropriate anti-blockers (B_{1a}'/B_{1b}'). The deblocked strand E_{1a} and strand E_{1b} correspond to the Mg^{2+}-ion-dependent DNAzyme subunits. The ATP induced unlocking of the origami tile, in the presence of the helper hairpins H_1 and H_2 leads to the formation of the nanocavity. The strands E_{1a} and E_{1b} bind together and form the active Mg^{2+}-ion-dependent DNAzyme that cleave the ROX/BHQ2-modified substrate S_1 to produce the ROX-modified fragment in the confined nanocavity. Treatment of the catalytic system with the blockers B_{1a} and B_{1b} separates the Mg^{2+}-ion-dependent DNAzyme subunits, and the

subsequent treatment of the tiles with C-ATP$_a$ and C-ATP$_a$', in the presence of the respective counter-helper units (H$_{1a}$'/H$_{1b}$'/H$_{2a}$'/H$_{2b}$'), leads to the closure of the nanocavity. The scheme represents the mechanistic path for the cyclic switching of the activity of the Mg^{2+}-ion-dependent DNAzyme in the confined nanocavity, associated with the origami tile. The reversible switching of the fluorescence of ROX provides the readout signal for the “ON”/“OFF” switching of the catalytic functions of the system.

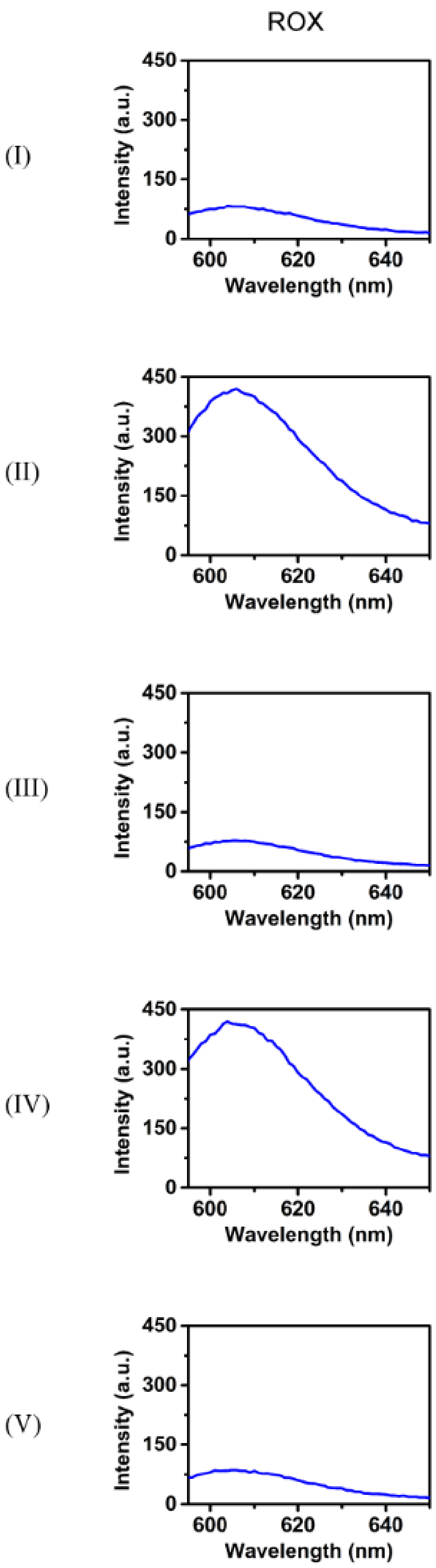

Figure S37. Fluorescence spectra corresponding to the cyclic activation and deactivation of the Mg^{2+}-ion-dependent DNAzyme in the ATP-responsive origami tiles. Panel I corresponds to the fluorescence response of the locked ATP-responsive origami tile. Panel II corresponds to the activated DNAzyme in the nanocavities as a result of the ATP, H_1/H_2-stimulated unlocking of the cavities. Panel III to Panel V represent the

fluorescence spectra of the system upon the cyclic closure-opening and reclosure of the nanocavities, The closed ATP-responsive origami tiles are generated by treatment of the open ATP-responsive origami tiles with the counter strands H_{1a}' and H_{1b}', H_{2a}' and H_{2b}', C-ATP$_a$ and C-ATP$_a$'. The re-opening of the ATP-responsive tiles involved the treatment of the closed state with ATP and the hairpins H_1/H_2.

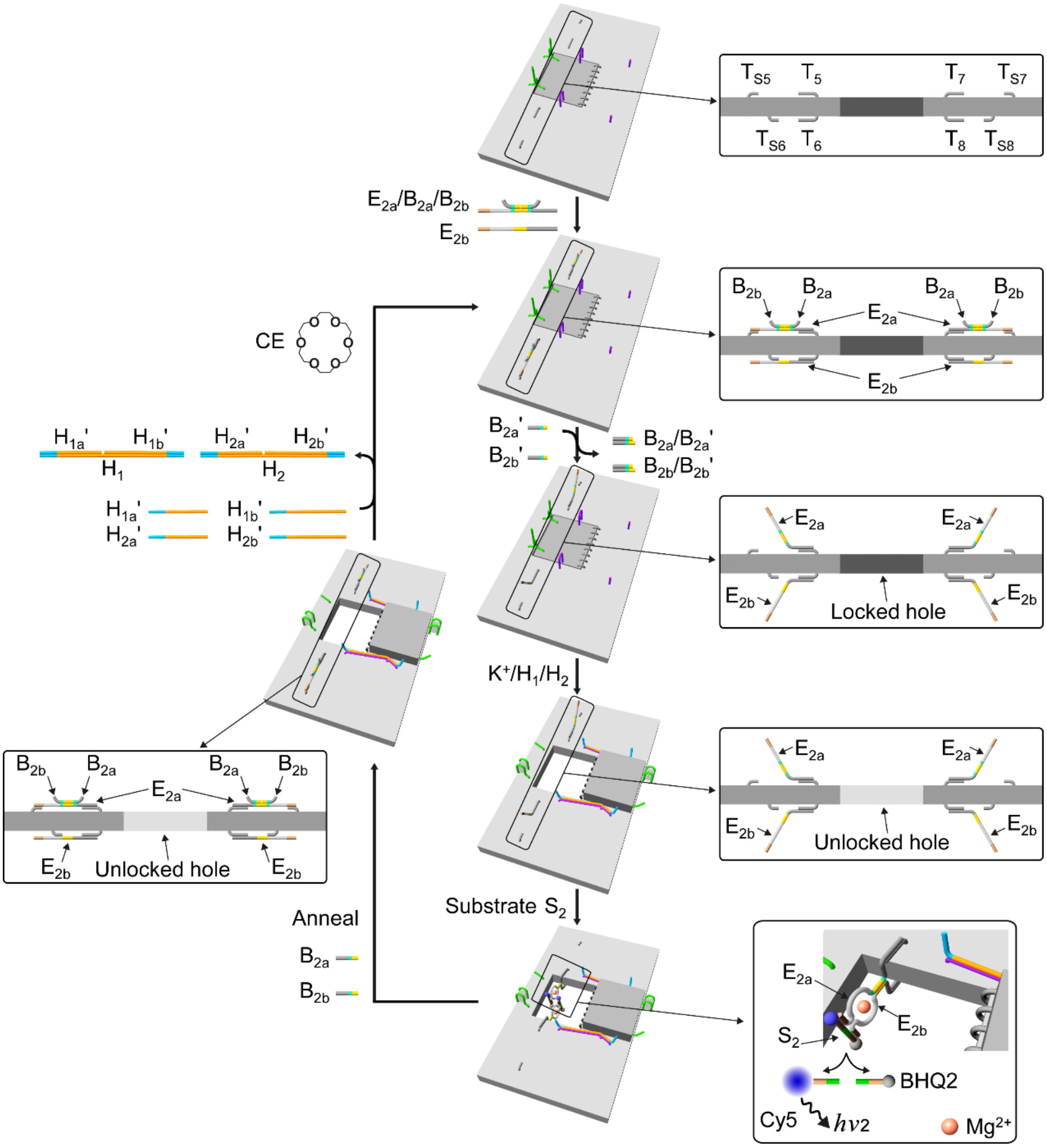

Figure S38. Schematic of engineering of the G-quadruplex-responsive origami tile (only showing the core part of the tile) and the switchable catalysis of the Mg^{2+}-ion-dependent DNAzyme in the nanocavity. The tile includes the K^+-ion-driven unlocking system. Protruding tethers T_5/T_7 and T_6/T_8 are designed on the opposite sides of the origami tile. The duplex E_{2a}/B_{2a}/B_{2b} and the strand E_{2b} are hybridized with the tethers T_5/T_7 and T_6/T_8, respectively. Prior to the unlocking of the tile, E_{2a}/B_{2a}/B_{2b} are unblocked by the strand displacement process, using appropriate anti-blockers (B_{2a}'/B_{2b}'). The deblocked strand E_{2a} and strand E_{2b} correspond to the Mg^{2+}-ion-dependent DNAzyme subunits. The K^+ ions induced unlocking of the origami tile, in the presence of the helper hairpins H_1 and H_2 leads to the formation of the nanocavity. The strands E_{2a} and E_{2b} assemble into formation of the active Mg^{2+}-ion-dependent DNAzyme that cleave the Cy5/BHQ2-modified substrate S_2 to produce the Cy5-modified fragment in the confined nanocavity. Treatment of the catalytic system with the blockers B_{2a} and B_{2b} separates the Mg^{2+}-ion-dependent DNAzyme subunits and the

subsequent treatment of the tiles with crown ether (CE), in the presence of the respective counter-helper units ($H_{1a}'/H_{1b}'/H_{2a}'/H_{2b}'$), leads to the closure of the nanocavity. The scheme represents the mechanistic path for the cyclic switching of the activity of the Mg^{2+}-ion-dependent DNAzyme in the confined nanocavity associated with the origami tile. The reversible switching of the fluorescence of Cy5 provides the readout signal for the "ON"/"OFF" switching of the catalytic functions of the system.

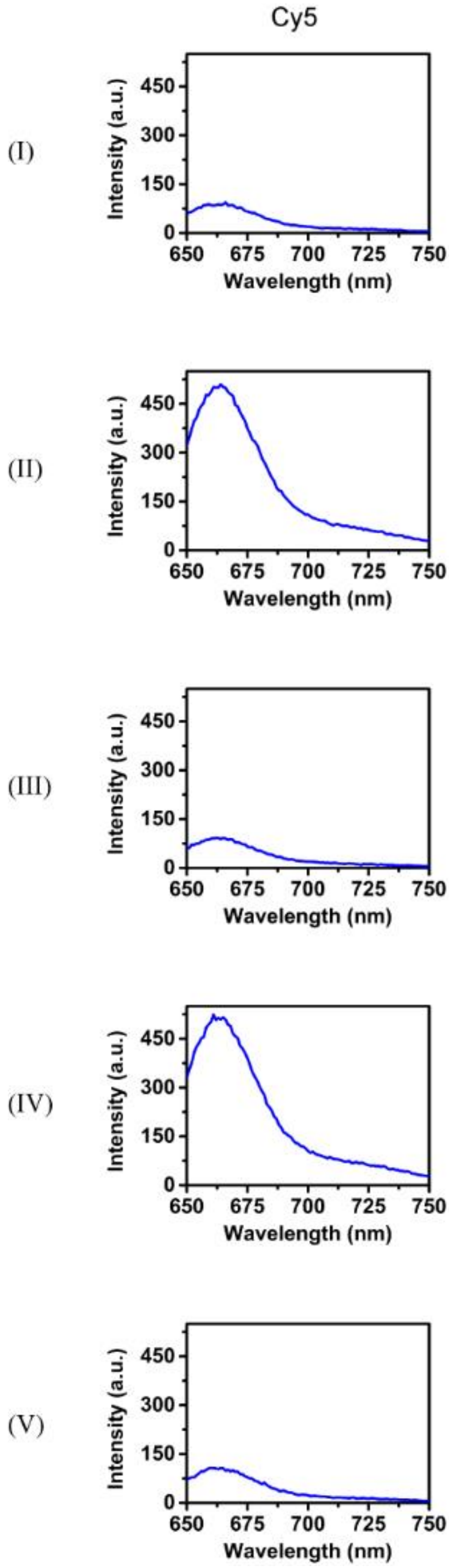

Figure S39. Fluorescence spectra corresponding to the cyclic activation and deactivation of the Mg^{2+}-ion-dependent DNAzyme in the G-quadruplex-responsive origami tiles. Panel I corresponds to the fluorescence response of the locked G-quadruplex-responsive origami tile. Panel II corresponds to the activated DNAzyme in the nanocavities as a result of the K^+ ions, H_1/H_2-stimulated unlocking of the cavities.

Panel III to Panel V represent the fluorescence spectra of the system upon the cyclic closure-opening and reclosure of the nanocavities, The closed G-quadruplex-responsive origami tiles are generated by treatment of the open origami tiles with the counter strands H_{1a}' and H_{1b}', H_{2a}' and H_{2b}', and crown ether (CE). The re-opening of the G-quadruplex-responsive tiles involved the treatment of the closed state with K^+ ions and the hairpins H_1/H_2.

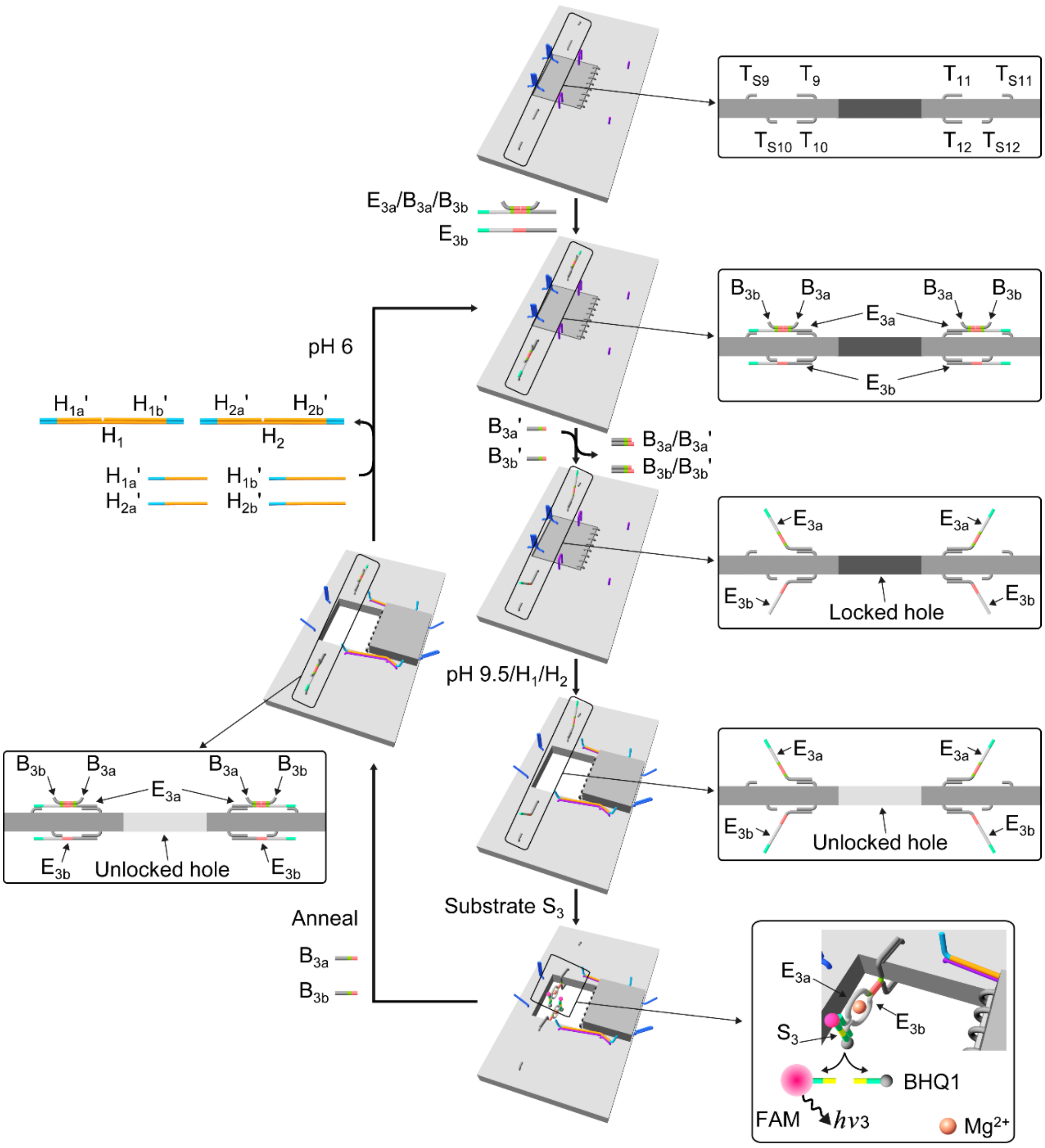

Figure S40. Schematic of engineering of the pH-responsive origami tile (only showing the core part of the tile) and the switchable catalysis of the Mg^{2+}-ion-dependent DNAzyme in the nanocavity. The tile includes the pH-driven unlocking system. Protruding tethers T_9/T_{11} and T_{10}/T_{12} are designed on the opposite sides of the origami tile. The duplex $E_{3a}/B_{3a}/B_{3b}$ and the strand E_{3b} are hybridized with the tethers T_9/T_{11} and T_{10}/T_{12}, respectively. Prior to the unlocking of the tile, $E_{3a}/B_{3a}/B_{3b}$ are unblocked by the strand displacement process, using appropriate anti-blockers (B_{3a}'/B_{3b}'). The deblocked strands E_{3a} and E_{3b} correspond to the Mg^{2+}-ion-dependent DNAzyme subunits. The pH-induced unlocking of the origami tiles, in the presence of the helper hairpins H_1 and H_2, leads to the formation of the nanocavities. The strands E_{3a} and E_{3b} assemble and form the active Mg^{2+}-ion-dependent DNAzyme that cleaves the FAM/BHQ1-modified substrate S_3 to produce the FAM-modified fragment in the confined nanocavity. Treatment of the catalytic system with the blockers B_{3a} and B_{3b} separates the Mg^{2+}-ion-dependent DNAzyme subunits, and the subsequent treatment of the tiles at pH = 6, and

in the presence of the respective counter-helper units (H_{1a}'/H_{1b}'/H_{2a}'/H_{2b}'), results in the closure of the nanocavities. The scheme represents the mechanistic path for the cyclic switching of the activity of the Mg^{2+}-ion-dependent DNAzyme in the confined nanocavity associated with the origami tile. The reversible switching of the fluorescence of FAM provides the readout signal for the “ON”/“OFF” switching of the catalytic functions of the system.

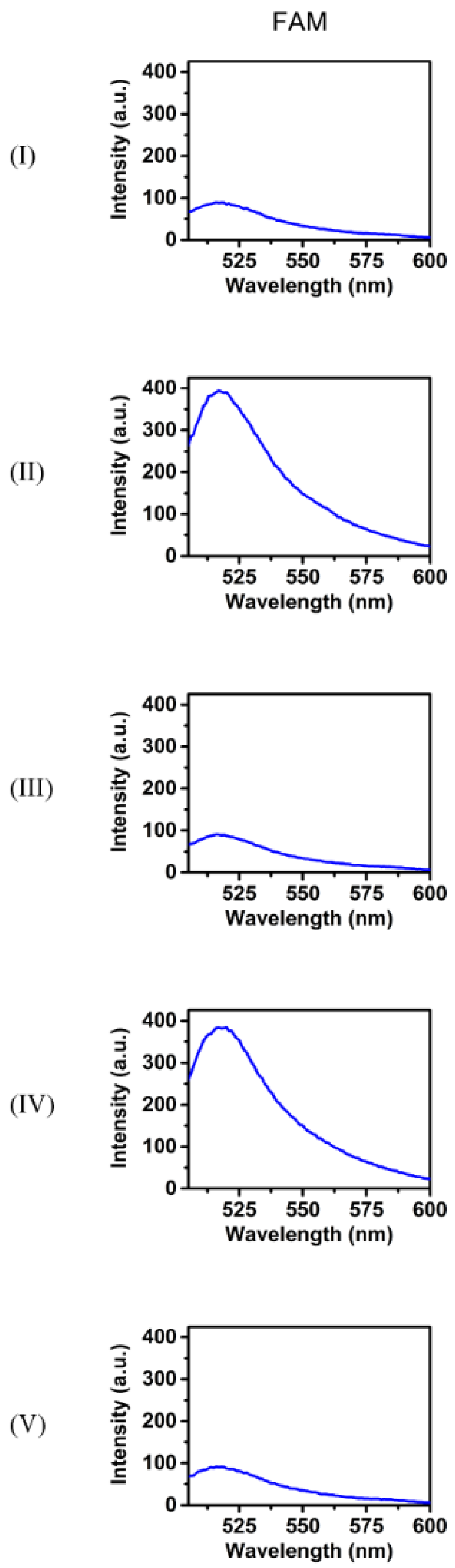

Figure S41. Fluorescence spectra corresponding to the cyclic activation and deactivation of the Mg^{2+}-ion-dependent DNAzyme in the pH-responsive origami tiles. Panel I corresponds to the fluorescence response of the locked pH-responsive origami tile. Panel II corresponds to the activated DNAzyme in the nanocavities as a result of the pH = 9.5, H_1/H_2-stimulated unlocking of the cavities. Panel III to Panel V represent

the fluorescence spectra of the system upon the cyclic closure-opening and reclosure of the nanocavities, The closed pH-responsive origami tiles are generated by treatment of the open origami tiles with the counter strands H_{1a}' and H_{1b}', H_{2a}' and H_{2b}', at pH = 6. The re-opening of the pH-responsive tiles involved the treatment of the closed state with K^+ ions and the hairpins H_1/H_2.

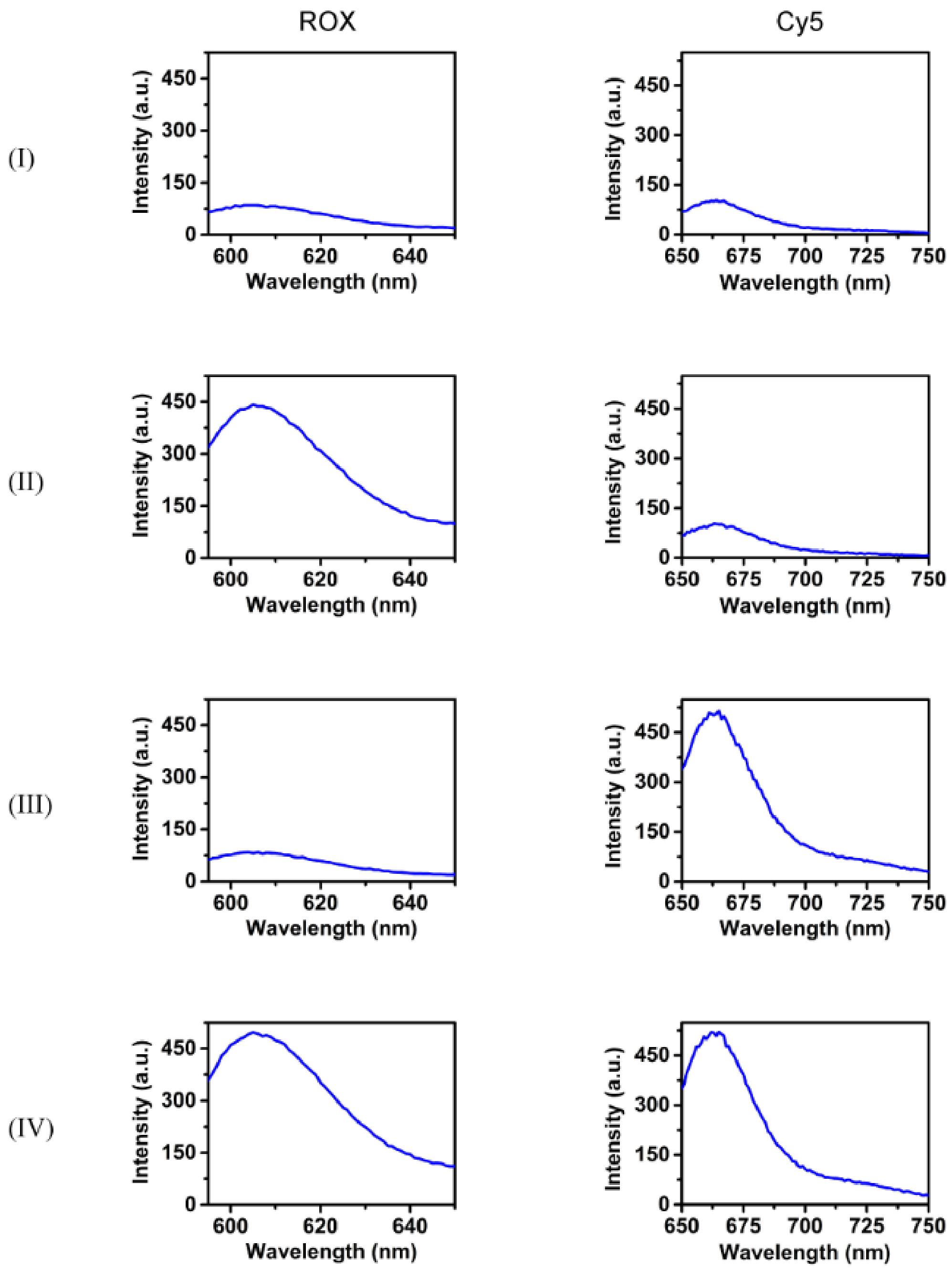

Figure S42. Fluorescence spectra corresponding to the programmed activation of the two Mg^{2+}-ion-dependent DNAzymes in the confined nanocavities in the ATP-/K^{+}-ion-responsive origami dimers. The programmed activation of the catalytic functions of the two Mg^{2+}-ion-dependent DNAzymes were performed by unlocking the nanocavities in the ATP-/K^{+}-ion-responsive origami dimer D_1 by treatment with the K^{+} ions and/or ATP and the help hairpins H_1/H_2. Panel I — Fluorescence spectra of ROX (left) and Cy5 (right) generated by the locked origami dimers (shown in panel I of Fig. 8). Panel II — Fluorescence spectra of ROX (left) and Cy5 (right) generated upon selective unlocking of the dimer on the left with ATP (cf. panel II in Fig. 8). The fluorescence spectrum of ROX shows the enhanced intensity over the background signal generated in the confined cavity. Panel III — Fluorescence spectra of ROX (left) and Cy5 (right) upon subjecting the dimer to K^{+} ions (cf. process shown in panel III in Fig. 8). No

fluorescence change of ROX above the background signal is observed, while the fluorescence of Cy5 is enhanced as compared to the background fluorescence signal. Panel IV — Fluorescence spectra of ROX (left) and Cy5 (right) generated upon subjecting the dimer to the K^+ ions and ATP (cf. process shown in panel IV of Fig. 8). The fluorescence of ROX and the fluorescence of Cy5 are intensified as compared to the background signals, consistent with the activation of the two DNAzymes in the nanocavities.

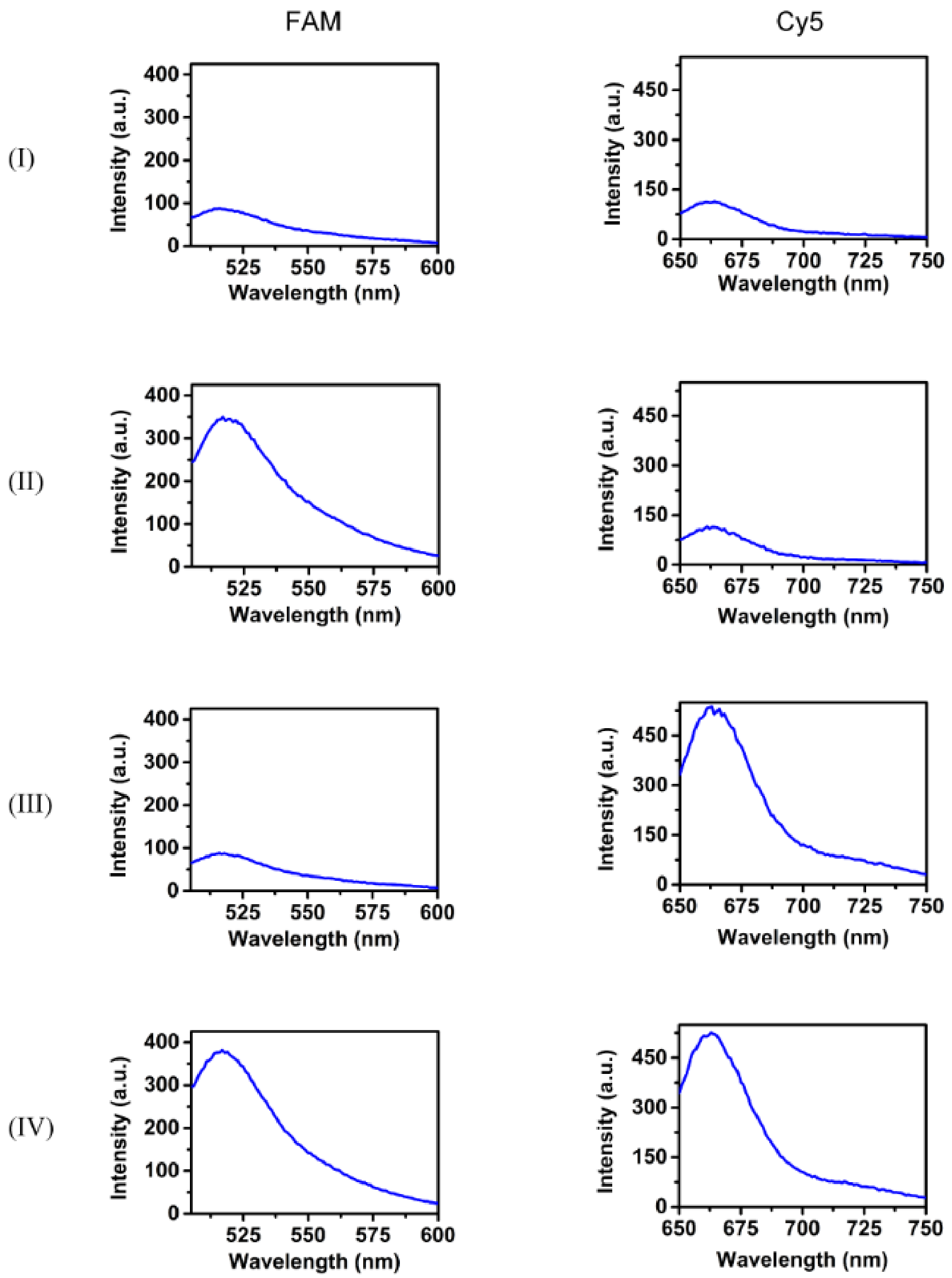

Figure S43. Fluorescence spectra corresponding to the programmed activation of the two Mg^{2+}-ion-dependent DNAzymes in the confined nanocavities of the pH-/K^{+}-ion-responsive origami dimers. The programmed activation of the catalytic functions of the two Mg^{2+}-ion-dependent DNAzymes were performed by unlocking the nanocavities in the pH-/K^{+}-ion-responsive origami dimer D_2 by treatment with the K^{+} ions and/or pH = 9.5 buffer and the help hairpins H_1/H_2. Panel I — Fluorescence spectra of FAM (left) and Cy5 (right) generated by the locked origami dimers D_2 (shown in panel I of Fig. 9). Panel II — Fluorescence spectra of FAM (left) and Cy5 (right) generated upon selective unlocking of the dimer D_2 on the left at pH = 9.5 (cf. panel II in Fig. 9). The fluorescence spectrum of FAM shows the enhanced intensity over the background signal generated in the confined cavity. Panel III — Fluorescence spectra of FAM (left) and Cy5 (right) upon subjecting the dimer D_2 to K^{+} ions (cf. process shown in panel III in Fig. 9). No

fluorescence change of FAM above the background signal is observed, while the fluorescence of Cy5 is enhanced as compared to the background fluorescence signal. Panel IV — Fluorescence spectra of FAM (left) and Cy5 (right) generated upon subjecting the dimer D_2 to the K^+ ions at pH = 9.5 (cf. process shown in panel IV of Fig. 9). The fluorescence of FAM and the fluorescence of Cy5 are intensified as compared to the background signals, consistent with the activation of the two DNAzymes in the nanocavities.

DNA Sequences

Staple sequences for the rectangle origami:

1	CAAGCCCAATAGGAAC CCATGTACAAACAGTT
2	AATGCCCCGTAACAGT GCCCGTATCTCCCTCA
3	TGCCTTGACTGCCTAT TTCGGAACAGGGATAG
4	GAGCCGCCCCACCACC GGAACCGCGACGGAAA
5	AACCAGAGACCCTCAG AACCGCCAGGGGTCAG
6	TTATTCATAGGGAAGG TAAATATTCATTCAGT
7	CATAACCCGAGGCATA GTAAGAGCTTTTTAAG
8	ATTGAGGGTAAAGGTG AATTATCAATCACCGG
9	AAAAGTAATATCTTAC CGAAGCCCTTCCAGAG
10	GCAATAGCGCAGATAG CCGAACAATTCAACCG
11	CCTAATTTACGCTAAC GAGCGTCTAATCAATA
12	TCTTACCAGCCAGTTA CAAAATAAATGAAATA
13	ATCGGCTGCGAGCATG TAGAAACCTATCATAT
14	CTAATTTATCTTTCCT TATCATTCATCCTGAA
15	GCGTTATAGAAAAAGC CTGTTTAGAAGGCCGG
16	GCTCATTTTCGCATTA AATTTTTGAGCTTAGA
17	AATTACTACAAATTCT TACCAGTAATCCCATC
18	TTAAGACGTTGAAAAC ATAGCGATAACAGTAC
19	TAGAATCCCTGAGAAG AGTCAATAGGAATCAT
20	CTTTTACACAGATGAA TATACAGTAAACAATT
21	TTTAACGTTCGGGAGA AACAATAATTTTCCCT
22	CGACAACTAAGTATTA GACTTTACAATACCGA
23	GGATTTAGCGTATTAA ATCCTTTGTTTTCAGG
24	ACGAACCAAAACATCG CCATTAAATGGTGGTT
25	GAACGTGGCGAGAAAG GAAGGGAACAAACTAT
26	TAGCCCTACCAGCAGA AGATAAAAACATTTGA
27	CGGCCTTGCTGGTAAT ATCCAGAACGAACTGA
28	CTCAGAGCCACCACCC TCATTTTCCTATTATT
29	CTGAAACAGGTAATAA GTTTTAACCCCTCAGA
30	AGTGTACTTGAAAGTA TTAAGAGGCCGCCACC
31	GCCACCACTCTTTTCA TAATCAAACCGTCACC
32	GTTTGCCACCTCAGAG CCGCCACCGATACAGG
33	GACTTGAGAGACAAAA GGGCGACAAGTTACCA
34	AGCGCCAACCATTTGG GAATTAGATTATTAGC
35	GAAGGAAAATAAGAGC AAGAAACAACAGCCAT
36	GCCCAATACCGAGGAA ACGCAATAGGTTTACC
37	ATTATTTAACCCAGCT ACAATTTTCAAGAACG
38	TATTTTGCTCCCAATC CAAATAAGTGAGTTAA
39	GGTATTAAGAACAAGA AAAATAATTAAAGCCA
40	TAAGTCCTACCAAGTA CCGCACTCTTAGTTGC

41	ACGCTCAAAATAAGAA TAAACACCGTGAATTT
42	AGGCGTTACAGTAGGG CTTAATTGACAATAGA
43	ATCAAAATCGTCGCTA TTAATTAACGGATTCG
44	CTGTAAATCATAGGTC TGAGAGACGATAAATA
45	CCTGATTGAAAGAAAT TGCGTAGACCCGAACG
46	ACAGAAATCTTTGAAT ACCAAGTTCCTTGCTT
47	TTATTAATGCCGTCAA TAGATAATCAGAGGTG
48	AGATTAGATTTAAAAG TTTGAGTACACGTAAA
49	AGGCGGTCATTAGTCT TTAATGCGCAATATTA
50	GAATGGCTAGTATTAA CACCGCCTCAACTAAT
51	CCGCCAGCCATTGCAA CAGGAAAAATATTTTT
52	CCCTCAGAACCGCCAC CCTCAGAACTGAGACT
53	CCTCAAGAATACATGG CTTTTGATAGAACCAC
54	TAAGCGTCGAAGGATT AGGATTAGTACCGCCA
55	CACCAGAGTTCGGTCA TAGCCCCCGCCAGCAA
56	TCGGCATTCCGCCGCC AGCATTGACGTTCCAG
57	AATCACCAAATAGAAA ATTCATATATAACGGA
58	TCACAATCGTAGCACC ATTACCATCGTTTTCA
59	ATACCCAAGATAACCC ACAAGAATAAACGATT
60	ATCAGAGAAAGAACTG GCATGATTTTATTTTG
61	TTTTGTTTAAGCCTTA AATCAAGAATCGAGAA
62	AGGTTTTGAACGTCAA AAATGAAAGCGCTAAT
63	CAAGCAAGACGCGCCT GTTTATCAAGAATCGC
64	AATGCAGACCGTTTTT ATTTTCATCTTGCGGG
65	CATATTTAGAAATACC GACCGTGTTACCTTTT
66	AATGGTTTACAACGCC AACATGTAGTTCAGCT
67	TAACCTCCATATGTGA GTGAATAAACAAAATC
68	AAATCAATGGCTTAGG TTGGGTTACTAAATTT
69	GCGCAGAGATATCAAA ATTATTTGACATTATC
70	AACCTACCGCGAATTA TTCATTTCCAGTACAT
71	ATTTTGCGTCTTTAGG AGCACTAAGCAACAGT
72	CTAAAATAGAACAAAG AAACCACCAGGGTTAG
73	GCCACGCTATACGTGG CACAGACAACGCTCAT
74	GCGTAAGAGAGAGCCA GCAGCAAAAAGGTTAT
75	GGAAATACCTACATTT TGACGCTCACCTGAAA
76	TATCACCGTACTCAGG AGGTTTAGCGGGGTTT
77	TGCTCAGTCAGTCTCT GAATTTACCAGGAGGT
78	GGAAAGCGACCAGGCG GATAAGTGAATAGGTG
79	TGAGGCAGGCGTCAGA CTGTAGCGTAGCAAGG
80	TGCCTTTAGTCAGACG ATTGGCCTGCCAGAAT
81	CCGGAAACACACCACG GAATAAGTAAGACTCC
82	ACGCAAAGGTCACCAA TGAAACCAATCAAGTT
83	TTATTACGGTCAGAGG GTAATTGAATAGCAGC

84	TGAACAAACAGTATGT TAGCAAACTAAAAGAA
85	CTTTACAGTTAGCGAA CCTCCCGACGTAGGAA
86	GAGGCGTTAGAGAATA ACATAAAAGAACACCC
87	TCATTACCCGACAATA AACAACATATTTAGGC
88	CCAGACGAGCGCCCAA TAGCAAGCAAGAACGC
89	AGAGGCATAATTTCAT CTTCTGACTATAACTA
90	TTTTAGTTTTTCGAGC CAGTAATAAATTCTGT
91	TATGTAAACCTTTTTT AATGGAAAAATTACCT
92	TTGAATTATGCTGATG CAAATCCACAAATATA
93	GAGCAAAAACTTCTGA ATAATGGAAGAAGGAG
94	TGGATTATGAAGATGA TGAAACAAAATTTCAT
95	CGGAATTATTGAAAGG AATTGAGGTGAAAAAT
96	ATCAACAGTCATCATA TTCCTGATTGATTGTT
97	CTAAAGCAAGATAGAA CCCTTCTGAATCGTCT
98	GCCAACAGTCACCTTG CTGAACCTGTTGGCAA
99	GAAATGGATTATTTAC ATTGGCAGACATTCTG
100	TTTT TATAAGTA TAGCCCGGCCGTCGAGAGGGTTGA
101	TTTT ATAAATCC TCATTAAATGATATTCACAAACAA
102	TTTT AATCAGTA GCGACAGATCGATAGCAGCACCGT
103	TTTT TAAAGGTG GCAACATAGTAGAAAATACATACA
104	TTTT GACGGGAG AATTAACTACAGGGAAGCGCATTA
105	TTTT GCTTATCC GGTATTCTAAATCAGATATAGAAG
106	TTTT CGACAAAA GGTAAAGTAGAGAATATAAAGTAC
107	TTTT CGCGAGAA AACTTTTTATCGCAAGACAAAGAA
108	TTTT ATTAATTA CATTTAACACATCAAGAAAACAAA
109	TTTT TTCATCAA TATAATCCTATCAGATGATGGCAA
110	TTTT AATCAATA TCTGGTCACAAATATCAAACCCTC
111	TTTT ACCAGTAA TAAAAGGGATTCACCA GTCACACG TTTT
112	CCGAAATCCGAAAATC CTGTTTGAAGCCGGAA
113	CCAGCAGGGGCAAAAT CCCTTATAAAGCCGGC
114	GCATAAAGTTCCACAC AACATACGAAGCGCCA
115	GCTCACAATGTAAAGC CTGGGGTGGGTTTGCC
116	TTCGCCATTGCCGGAA ACCAGGCATTAAATCA
117	GCTTCTGGTCAGGCTG CGCAACTGTGTTATCC
118	GTTAAAATTTTAACCA ATAGGAACCCGGCACC
119	AGACAGTCATTCAAAA GGGTGAGAAGCTATAT
120	AGGTAAAGAAATCACC ATCAATATAATATTTT
121	TTTCATTTGGTCAATA ACCTGTTTATATCGCG
122	TCGCAAATGGGGCGCG AGCTGAAATAATGTGT
123	TTTTAATTGCCCGAAA GACTTCAAAACACTAT
124	AAGAGGAACGAGCTTC AAAGCGAAGATACATT
125	GGAATTACTCGTTTAC CAGACGACAAAAGATT
126	GAATAAGGACGTAACA AAGCTGCTCTAAAACA

127	CCAAATCACTTGCCCT GACGAGAACGCCAAAA
128	CTCATCTTGAGGCAAA AGAATACAGTGAATTT
129	AAACGAAATGACCCCC AGCGATTATTCATTAC
130	CTTAAACATCAGCTTG CTTTCGAGCGTAACAC
131	TCGGTTTAGCTTGATA CCGATAGTCCAACCTA
132	TGAGTTTCGTCACCAG TACAAACTTAATTGTA
133	CCCCGATTTAGAGCTT GACGGGGAAATCAAAA
134	GAATAGCCGCAAGCGG TCCACGCTCCTAATGA
135	GAGTTGCACGAGATAG GGTTGAGTAAGGGAGC
136	GTGAGCTAGTTTCCTG TGTGAAATTTGGGAAG
137	TCATAGCTACTCACAT TAATTGCGCCCTGAGA
138	GGCGATCGCACTCCAG CCAGCTTTGCCATCAA
139	GAAGATCGGTGCGGGC CTCTTCGCAATCATGG
140	AAATAATTTTAAATTG TAAACGTTGATATTCA
141	GCAAATATCGCGTCTG GCCTTCCTGGCCTCAG
142	ACCGTTCTAAATGCAA TGCCTGAGAGGTGGCA
143	TATATTTTAGCTGATA AATTAATGTTGTATAA
144	TCAATTCTTTTAGTTT GACCATTACCAGACCG
145	CGAGTAGAACTAATAG TAGTAGCAAACCCTCA
146	GAAGCAAAAAAGCGGA TTGCATCAGATAAAAA
147	TCAGAAGCCTCCAACA GGTCAGGATCTGCGAA
148	CCAAAATATAATGCAG ATACATAAACACCAGA
149	CATTCAACGCGAGAGG CTTTTGCATATTATAG
150	ACGAGTAGTGACAAGA ACCGGATATACCAAGC
151	AGTAATCTTAAATTGG GCTTGAGAGAATACCA
152	GCGAAACATGCCACTA CGAAGGCATGCGCCGA
153	ATACGTAAAAGTACAA CGGAGATTTCATCAAG
154	CAATGACACTCCAAAA GGAGCCTTACAACGCC
155	AAAAAAGGACAACCAT CGCCCACGCGGGTAAA
156	TGTAGCATTCCACAGA CAGCCCTCATCTCCAA
157	GTAAAGCACTAAATCG GAACCCTAGTTGTTCC
158	AGTTTGGAGCCCTTCA CCGCCTGGTTGCGCTC
159	AGCTGATTACAAGAGT CCACTATTGAGGTGCC
160	ACTGCCCGCCGAGCTC GAATTCGTTATTACGC
161	CCCGGGTACTTTCCAG TCGGGAAACGGGCAAC
162	CAGCTGGCGGACGACG ACAGTATCGTAGCCAG
163	GTTTGAGGGAAAGGGG GATGTGCTAGAGGATC
164	CTTTCATCCCCAAAAA CAGGAAGACCGGAGAG
165	AGAAAAGCAACATTAA ATGTGAGCATCTGCCA
166	GGTAGCTAGGATAAAA ATTTTTAGTTAACATC
167	CAACGCAATTTTTGAG AGATCTACTGATAATC
168	CAATAAATACAGTTGA TTCCCAATTTAGAGAG
169	TCCATATACATACAGG CAAGGCAACTTTATTT

170	TACCTTTAAGGTCTTT ACCCTGACAAAGAAGT
171	CAAAAATCATTGCTCC TTTTGATAAGTTTCAT
172	TTTGCCAGATCAGTTG AGATTTAGTGGTTTAA
173	AAAGATTCAGGGGGTA ATAGTAAACCATAAAT
174	TTTCAACTATAGGCTG GCTGACCTTGTATCAT
175	CCAGGCGCTTAATCAT TGTGAATTACAGGTAG
176	CGCCTGATGGAAGTTT CCATTAAACATAACCG
177	TTTCATGAAAATTGTG TCGAAATCTGTACAGA
178	ATATATTCTTTTTTCA CGTTGAAAATAGTTAG
179	AATAATAAGGTCGCTG AGGCTTGCAAAGACTT
180	CGTAACGATCTAAAGT TTTGTCGTGAATTGCG
181	ACCCAAATCAAGTTTT TTGGGGTCAAAGAACG
182	TGGACTCCCTTTTCAC CAGTGAGACCTGTCGT
183	TGGTTTTTAACGTCAA AGGGCGAAGAACCATC
184	GCCAGCTGCCTGCAGG TCGACTCTGCAAGGCG
185	CTTGCATGCATTAATG AATCGGCCCGCCAGGG
186	ATTAAGTTCGCATCGT AACCGTGCGAGTAACA
187	TAGATGGGGGGTAACG CCAGGGTTGTGCCAAG
188	ACCCGTCGTCATATGT ACCCCGGTAAAGGCTA
189	CATGTCAAGATTCTCC GTGGGAACCGTTGGTG
190	TCAGGTCACTTTTGCG GGAGAAGCAGAATTAG
191	CTGTAATATTGCCTGA GAGTCTGGAAAACTAG
192	CAAAATTAAAGTACGG TGTCTGGAAGAGGTCA
193	TGCAACTAAGCAATAA AGCCTCAGTTATGACC
194	TTTTTGCGCAGAAAAC GAGAATGAATGTTTAG
195	AAACAGTTGATGGCTT AGAGCTTATTTAAATA
196	ACTGGATAACGGAACA ACATTATTACCTTATG
197	ACGAACTAGCGTCCAA TACTGCGGAATGCTTT
198	CGATTTTAGAGGACAG ATGAACGGCGCGACCT
199	CTTTGAAAAGAACTGG CTCATTATTTAATAAA
200	GCTCCATGAGAGGCTT TGAGGACTAGGGAGTT
201	ACGGCTACTTACTTAG CCGGAACGCTGACCAA
202	AAAGGCCGAAAGGAAC AACTAAAGCTTTCCAG
203	GAGAATAGCTTTTGCG GGATCGTCGGGTAGCA
204	ACGTTAGTAAATGAAT TTTCTGTAAGCGGAGT
205	TTTT CGATGGCC CACTACGTAAACCGTC TATCAGGG
206	TTTT CGGTTTGC GTATTGGGAACGCGCG GGGAGAGG
207	TTTT TGTAAAAC GACGGCCATTCCCAGT CACGACGT
208	TTTT GTAATGGG ATAGGTCAAAACGGCG GATTGACC
209	TTTT GATGAACG GTAATCGTAGCAAACA AGAGAATC
210	TTTT GGTTGTAC CAAAAACAAGCATAAA GCTAAATC
211	TTTT CTGTAGCT CAACATGTATTGCTGA ATATAATG
212	TTTT CATTGAAT CCCCCTCAAATCGTCA TAAATATT

213	TTTT GGAAGAAA AATCTACGACCAGTCA GGACGTTG
214	TTTT TCATAAGG GAACCGAAAGGCGCAG ACGGTCAA
215	TTTT GACAGCAT CGGAACGAACCCTCAG CAGCGAAA
216	TTTT AACTTTCA ACAGTTTCTGGGATTT TGCTAAAC TTTT
Loop1	AACATCACTTGCCTGAGTAGAAGAACT
Loop2	TGTAGCAATACTTCTTTGATTAGTAAT
Loop3	AGTCTGTCCATCACGCAAATTAACCGT
Loop4	ATAATCAGTGAGGCCACCGAGTAAAAG
Loop5	ACGCCAGAATCCTGAGAAGTGTTTTT
Loop6	TTAAAGGGATTTTAGACAGGAACGGT
Loop7	AGAGCGGGAGCTAAACAGGAGGCCGA
Loop8	TATAACGTGCTTTCCTCGTTAGAATC
Loop9	GTACTATGGTTGCTTTGACGAGCACG
Loop10	GCGCTTAATGCGCCGCTACAGGGCGC

DNA sequences for the nano-cavities and the catalysis:

L(1)	GGGGTTAGGGGTTAGGGGTTAGGGGAGACTTTTTTTTTAAGAA AAGTAATATCTTACCGAAGCCCTTCCAGAG
L(2)	GGGGTTAGGGGTTAGGGGTTAGGGGGTGATTTAAGGCCGGAG ACAGTCATTCAAAAGGGTGAGAAGCTATAT
L'(1)	CATAACCCGAGGCATAGTAAGAGCTTTTGTCTCTTCTAAC
L'(2)	GCGTTATAGAAAAAGCCTGTTTAGTTTTTCACCTTCTAAC
H_a	AATAGTGAATTTATCAAATTTTAAATTCAACTTTTTTACCATTTTA ATTTTAACCTTTTCCTTTTAATAAGAATAAACACC
H_b	ACAAAGTTACCAGAAGGATTTTCATCCATTCATTTTCATTACTTT AATTCCTTAATTCTTTTCTTAGACAAAAGGGCGACA
A_1	TTGGAAAAGGTTTTTTCTTCTGACTATAACTA
A_2	CCGGAAACACACCACGTTTTTGAATGGATGTT
H_1	CAGCACACCTAGTTAACCTTTTCCTTTAAAAAGGAAAAGGTTA AAATTAAAATGGTAAAAAAGTTGAATTTAAAACGAGAGGTCAT G
H_2	GTCAGGTCACAGAAGAAAAGAATTAAGGAATTAAAGTAATGAA AATGAATGGATGAAAAAATTCATCCATTCATTCCTACAGGTCAG
H_{1a}'	CATGACCTCTCGTTTTAAATTCAACTTTTTTACCATTTTA
H_{1b}'	ATTTTAACCTTTTCCTTTTTAAAGGAAAAGGTTAACTAGGTGTG CTG
H_{2a}'	ATTACTTTAATTCCTTAATTCTTTTCTTCTGTGACCTGAC
H_{2b}'	CTGACCTGTAGGAATGAATGGATGAATTTTTTCATCCATTCATTT TC
$H_{a\text{-}F}$	AATAGTGAATTTATCAAATTTTAAATTCAACTTTTTTACCATTTTA ATTTTAACCTTTTCCTTTT[Cy3]AATAAGAATAAACACC
$A_{1\text{-}F}$	Cy5-TTGGAAAAGGTTTTTTCTTCTGACTATAACTA

H_{1-F}	GAAGAAAAAACCTTTTCCTTTAAAAAGGAAAAGGTTAAAATTA AAATGGTAAAAAAGTTGAATTTAAAA
M(1)	CATAACCCGAGGCATAGTAATTTTTTACCTGGGGGAGTATTGCG GAGGAAGGTCCACTGTC
M(2)	GCGTTATAGAAAAAGCCTGTAATTTTACCTGGGGGAGTATTGCG GAGGAAGGTCCACTGTC
M'(1)	ACTCCCCCAGGTTTTTAATAAGAAAAGTAATATCTTACCGAAGC CCTTCCAGAG
M'(2)	ACTCCCCCAGGTTTTTTTCCGGAGACAGTCATTCAAAA
C-ATP_a	CCTCAGACACGACAGTGGACCTTCCTCCGC
C-ATP_a'	GCGGAGGAAGGTCCACTGTCGTGTCTGAGG
N(1)	CATAACCCGAGGCATAGTAAGAGCTTTTTTCTTTTCTTTTCTTTT CTTTTTTAAGAAAAGAAAAGAAAAGAA
N(2)	GCGTTATAGAAAAAGCCTGTTTAGTTTTTTCTTTTCTTTTCTTTT CTTTTTTAAGAAAAGAAAAGAAAAGAA
N'(1)	TTCTTTTCTTTTCTTTTCTTTTTTTTTTTAAGAAAAGTAATATCTT ACCGAAGCCCTTCCAGAG
N'(2)	TTCTTTTCTTTTCTTTTCTTTTTTAAGGCCGGAGACAGTCATTCA AAAGGGTGAGAAGCTATAT
T_1	TTAAGACGTTGAAAACTTTTTGTAATGTGTCTCGTTCTG
T_2	GTTGTAGAGCACTGTGGCTTTTTAGAATCCCTGAGAAGAGTC
T_3	TTCAACCGATTGAGGGTAAAGGTGTTTTTGTAATGTGTCTCGTT CTG
T_4	GTTGTAGAGCACTGTGGCTTTTTTATTCATAGGGAAGGTAAATAT TCATTCAGT
E_{1a}	CTGCTCAGCGATTAACACCTGTGATGCAGAGACCTGGAATTTTT TCAGAACGAGACACATTAC
E_{1b}	GCCACAGTGCTCTACAACTTTTTCAGGTCTCTGCATCACGTTAC ACCCATGT TCGTCA
S_1	ROX-TGACGATrAGGAGCAG-BHQ2
T_5	TTAAGACGTTGAAAACTTTTTGAGCTGTATCACACGTAC
T_6	GTAGTCCAACCTGTCTACTTTTTAGAATCCCTGAGAAGAGTC
T_7	TTCAACCGATTGAGGGTAAAGGTGTTTTTGAGCTGTATCACACG TAC
T_8	GTAGTCCAACCTGTCTACTTTTTATTCATAGGGAAGGTAAATATT CATTCAGT
E_{2a}	CTGCTCAGCGATTAACGATGGTCGTCTACAGACTGCCAGATTTT TGTACGTGTGATACAGCTC
E_{2b}	GTAGACAGGTTGGACTACTTTTTGCAGTCTGTAGACGACGTTAC ACCCATGTTACTCT
S_2	Cy5-AGAGTATrAGGAGCAG-BHQ2
T_9	TTAAGACGTTGAAAACTTTTTCATGATGGACACTCGATC
T_{10}	GACTGGTCTGCTCATTACTTTTTAGAATCCCTGAGAAGAGTC

T_{11}	TTCAACCGATTGAGGGTAAAGGTGTTTTTCATGATGGACACTCGATC
T_{12}	GACTGGTCTGCTCATTACTTTTTATTCATAGGGAAGGTAAATATTCATTCAGT
E_{3a}	GATATCAGCGATTAACGGTCCAACCTGTAGTTGCTCCTTCTTTTTGATCGAGTGTCCATCATG
E_{3b}	GTAATGAGCAGACCAGTCTTTTTGAGCAACTACAGGTTGGTTACACCCATGTTACTCT
S_3	FAM-AGAGTATrAGGATATC-BHQ1
T_{S1}	CTGAGCAGTTTCTGTTTGAAGCCGGAA
T_{S2}	GGATTTAGCGTATTAAATCCTTTGTTTTCAGGTTTGTAACGTG
T_{S3}	CTGAGCAGTTTTCATTTTCCTATTATT
T_{S4}	AATGCCCCGTAACAGTGCCCGTATCTCCCTCATTTGTAACGTG
B_{1a}	CATCCGTCATTCCAGGTCTC
B_{1b}	TGCATCACAGGTAGCCCCTT
B_{1a}'	CCTGGAATGACGGATG
B_{1b}'	AAGGGGCTACCTGTGA
T_{S5}	CTGAGCAGTTTCTGTTTGAAGCCGGAA
T_{S6}	GGATTTAGCGTATTAAATCCTTTGTTTTCAGGTTTGTAACGTC
T_{S7}	CTGAGCAGTTTTCATTTTCCTATTATT
T_{S8}	AATGCCCCGTAACAGTGCCCGTATCTCCCTCATTTGTAACGTC
B_{2a}	TACCCTTCTCTGGCAGTCTG
B_{2b}	TAGACGACCATCAGTGCTCC
B_{2a}'	CTGCCAGAGAAGGGTA
B_{2b}'	GGAGCACTGATGGTCG
T_{S9}	GCTGATATCTTTCTGTTTGAAGCCGGAA
T_{S10}	GGATTTAGCGTATTAAATCCTTTGTTTTCAGGTTTGTAACCAAC
T_{S11}	GCTGATATCTTTTCATTTTCCTATTATT
T_{S12}	AATGCCCCGTAACAGTGCCCGTATCTCCCTCATTTGTAACCAAC
B_{3a}	GTCCGTAGGAAGGAGCAACT
B_{3b}	ACAGGTTGGACCTATCGTTC
B_{3a}'	GCTCCTTCCTACGGAC
B_{3b}'	GAACGATAGGTCCAAC
N8	TTCAACCGATTGAGGGTAAAGGTGAATTATCAATCACCGG
N10	GCAATAGCGCAGATAGCCGA
N17	GGAATCATAATTACTACAAATTCTTACCAGTAATCCCATC
N19	TAGAATCCCTGAGAAGAGTC
N31	GCCACCACTCTTTTCATAATCAAACCGTCACCGACTTGAG
N34	GGTTTACCAGCGCCAACCATTTGGGAATTAGATTATTAGC
N35	ATAAGAGCAAGAAACAACAGCCAT
N36	GCCCAATACCGAGGAA
N38	TATTTTGCTCCCAATC
N39	GGTATTAAGAACAAGAAAAATAATTAAAGCCAACGCTCAA

N40	TAAGTCCTACCAAGTA
N42	AGGCGTTACAGTAGGG
N43	CGTCGCTATTAATTAACGGATTCG
N44	CTGTAAATCATAGGTCTGAGAGAC
N57	AATCACCAAATAGAAAATTCATAT
N59	ATACCCAAGATAACCC
N61	TTTTGTTTAAGCCTTA
N63	CAAGCAAGACGCGCCT
N65	CATATTTAGAAATACC
N67	TACCTTTTTAACCTCCATATGTGAGTGAATAAACAAAATC
N81	GAATAAGTAAGACTCC
N89	AGAGGCATAATTTTCAT
N121	TTTCATTTGGTCAATA
N123	TTTTAATTGCCCGAAA
N28	CTCAGAGCCACCACCC
N-8	AATTATCAATCACCGG
N18	ATAGCGATAACAGTAC
N112	CCGAAATCCGAAAATC
L216	GTAGACAGTGTGTTTAACTTTCAACAGTTTCTGGGATTTTGCTAAACTTTGACGAACTGACC
L214	GTAGACAGTGTGTTTTCATAAGGGAACCGAAAGGCGCAGACGGTCAATTTGACGAACTGACC
L212	GTAGACAGTGTGTTTCATTGAATCCCCCTCAAATCGTCATAAATATTTTTGACGAACTGACC
L210	CATCGTGTAGTCTTTGGTTGTACCAAAAACAAGCATAAAGCTAAATCTTTCTGAACATGAGC
L208	CATCGTGTAGTCTTTGTAATGGGATAGGTCAAAACGGCGGATTGACCTTTCTGAACATGAGC
L206	CATCGTGTAGTCTTTCGGTTTGCGTATTGGGAACGCGCGGGGAGAGGTTTCTGAACATGAGC
L100	TATAAGTATAGCCCGGCCGTCGAGAGGGTTGATTTGGTCAGTTCGTC
L101	CACACTGTCTACTTTATAAATCCTCATTAAATGATATTCACAAACAA
L102	AATCAGTAGCGACAGATCGATAGCAGCACCGTTTTGGTCAGTTCGTC
L103	CACACTGTCTACTTTTAAAGGTGGCAACATAGTAGAAAATACATACA
L104	GACGGGAGAATTAACTACAGGGAAGCGCATTATTTGGTCAGTTCGTC
L105	CACACTGTCTACTTTGCTTATCCGGTATTCTAAATCAGATATAGAAG
L106	CGACAAAAGGTAAAGTAGAGAATATAAAGTACTTTGCTCATGTT

	CAG
L107	GACTACACGATGTTTCGCGAGAAAACTTTTTATCGCAAGACAA AGAA
L108	ATTAATTACATTTAACACATCAAGAAAACAAATTTGCTCATGTTC AG
L109	GACTACACGATGTTTTTCATCAATATAATCCTATCAGATGATGGC AA
L110	AATCAATATCTGGTCACAAATATCAAACCCTCTTTGCTCATGTTC AG
L111	GACTACACGATGTTTACCAGTAATAAAAGGGATTCACCAGTCAC ACG
MH160	ACTGCCCGCCGAGCTCATGAATCCTTTTGGATTCATCAAGTGCT TTTTAGCACTTGGAATTCGTTATTACGC
MH162	CAGCTGGCGGACGACGATGAATCCTTTTGGATTCATCAAGTGCT TTTTAGCACTTGACAGTATCGTAGCCAG
MH163	GTTTGAGGGAAAGGGGATGAATCCTTTTGGATTCATCAAGTGCT TTTTAGCACTTGGATGTGCTAGAGGATC
MH165	AGAAAAGCAACATTAAATGAATCCTTTTGGATTCATCAAGTGCT TTTTAGCACTTGATGTGAGCATCTGCCA
